# *Nicotiana benthamiana* as a Source of Cowpea Mosaic Virus‐Derived Particles That Specifically Package Designer RNAs

**DOI:** 10.1111/pbi.70294

**Published:** 2025-08-06

**Authors:** H. Peyret, S. N. Shah, Y. Meshcheriakova, J.‐W. Jung, K. Saunders, G. P. Lomonossoff

**Affiliations:** ^1^ Department of Biochemistry and Metabolism John Innes Centre Norwich UK; ^2^ University of Nottingham Sutton Bonnington Campus School of Biosciences, Division of Plant and Crop Sciences Loughborough UK; ^3^ Department of Life Science Jeonbuk National University Jeon‐buk‐do Republic of Korea

**Keywords:** cargo RNA, cowpea mosaic virus, encapsidation, *Nicotiana benthamiana*, RNA delivery, RNA protection, untranslated regions, virus‐like particles

## Abstract

The coupling of replication and encapsidation in the bipartite plant virus, cowpea mosaic virus (CPMV), has been used to specifically incorporate designer RNA molecules into viral particles via transient expression in *Nicotiana benthamiana*. This is achieved by placing the desired RNA sequence between the 5′ and 3′ UTRs of CPMV RNA‐2 so that the cargo RNA can be recognised by the RNA‐1‐encoded replicase. Replication and encapsidation of cargo RNA are achieved by co‐infiltrating with CPMV RNA‐1 to provide replication functions and a non‐replicating construct that encodes the precursor of the viral coat proteins, VP60. Encapsidation within CPMV particles stabilises the RNA such that it can be stored for prolonged periods at +4°C. Investigation of the length and sequence of the RNA that can be efficiently incorporated showed that a wide range of different RNA molecules are tolerated, provided that the length does not significantly exceed that of RNA‐1 (6.0 kb). In contrast to previously published conclusions, we show here that a self‐replicating version of RNA‐1 is not required for the replication and packaging of cargo RNA constructs. Use of a non‐replicating version of RNA‐1 not only eliminates the packaging of RNA‐1 but also increases the yield of particles containing the desired cargo RNA. Such particles have the potential to be used as delivery systems for bespoke RNAs to target cells.

## 1 Introduction

The recent COVID‐19 pandemic has led to an upsurge of interest in methods for delivering bespoke RNA molecules to cells. For example, RNAs that can be translated can be used to induce a specific immune response in target cells, leading to the concept of RNA‐based vaccines (Brandi et al. 2024; Yaremenko et al. 2025). One of the major challenges for the deployment of RNA‐based immunotherapies is the need to stabilise the labile RNA molecules so that they survive long enough to be delivered to cells. This has led to the development of a number of strategies to encapsulate the RNAs in nanoparticles, including virus‐like particles (VLPs), so that they can be delivered effectively (Vosoughi et al. 2025). Among the VLPs that have been used for this purpose are ones derived from plant viruses, such as tobacco mosaic virus (TMV; Zhou et al. 2015) and cowpea chlorotic mottle virus (CCMV; Azizgolshani et al. 2013).

Our previous work has shown that designer RNA molecules can be specifically encapsidated into particles of cowpea mosaic virus (CPMV; genus *Comovirus*), a bipartite plant‐infecting virus within the order *Picornavirales*, by making use of the tight linkage between RNA replication and encapsidation (Kruse et al. 2019). Encapsidation of RNA within CPMV particles protects it from degradation and this property has been used to generate positive controls for RT‐qPCR diagnostic reactions (King et al. 2007; Madi et al. 2015; Papamatthaiou et al. 2023; Peyret et al. 2022). The specific encapsidation of the target RNA is currently achieved through the use of a three‐component system. The sequence to be encapsidated is linked to the 5′ and 3′ UTRs of CPMV RNA‐2. This enables RNA derived from the construct to be replicated by the replication functions supplied by RNA‐1. The third component is a non‐replicating construct designed to provide a source of the viral coat proteins since these sequences are not present on the replicating RNA‐2‐based construct harbouring the target sequence (Peyret and Lomonossoff 2022). When all three components are expressed in *Nicotiana benthamiana* via *Agrobacterium*‐mediated transient expression, particles are produced that contain either RNA‐1, the target RNA, or are devoid of RNA (empty, or eVLPs). Recent research has shown that it is possible to deliver GFP mRNA encapsidated within CPMV particles to mammalian cells and that this RNA is released and can be translated within the cells (Roberts et al. 2024). This suggests that CPMV VLPs may be an effective way to stabilise RNA molecules intended for immunotherapy. The use of CPMV is attractive for such an application because the particles are very stable, easy to purify, and have been shown to be well‐tolerated within mammals (Affonso de Oliveira et al. 2022; Rae et al. 2005; Singh et al. 2007).

Despite the promise of CPMV as an RNA delivery system, to date the largest heterologous RNA that has been encapsidated has been the 1.5 kb mRNA for GFP. Though useful for the production of controls for RT‐PCR‐based diagnostic reactions or the expression of small marker genes, such as GFP, the encapsidation of significantly longer sequences is likely to be necessary for the approach to be generally useful for immunotherapy. Likewise, the quantity of particles containing the target sequence will need to be greater than has been obtained previously. We have therefore investigated the length and sequence of target cargo RNA that can be successfully incorporated into CPMV particles, and we show here that it is possible to eliminate the encapsidation of RNA‐1 by removing its ability to self‐replicate. By maximising the translatability of the non‐replicating RNA‐1, it is also possible to increase the level of target RNA encapsidation.

## 2 Results

### 2.1 RNAs of Several kb Can Be Stabilised by Encapsidation

**FIGURE 1:**
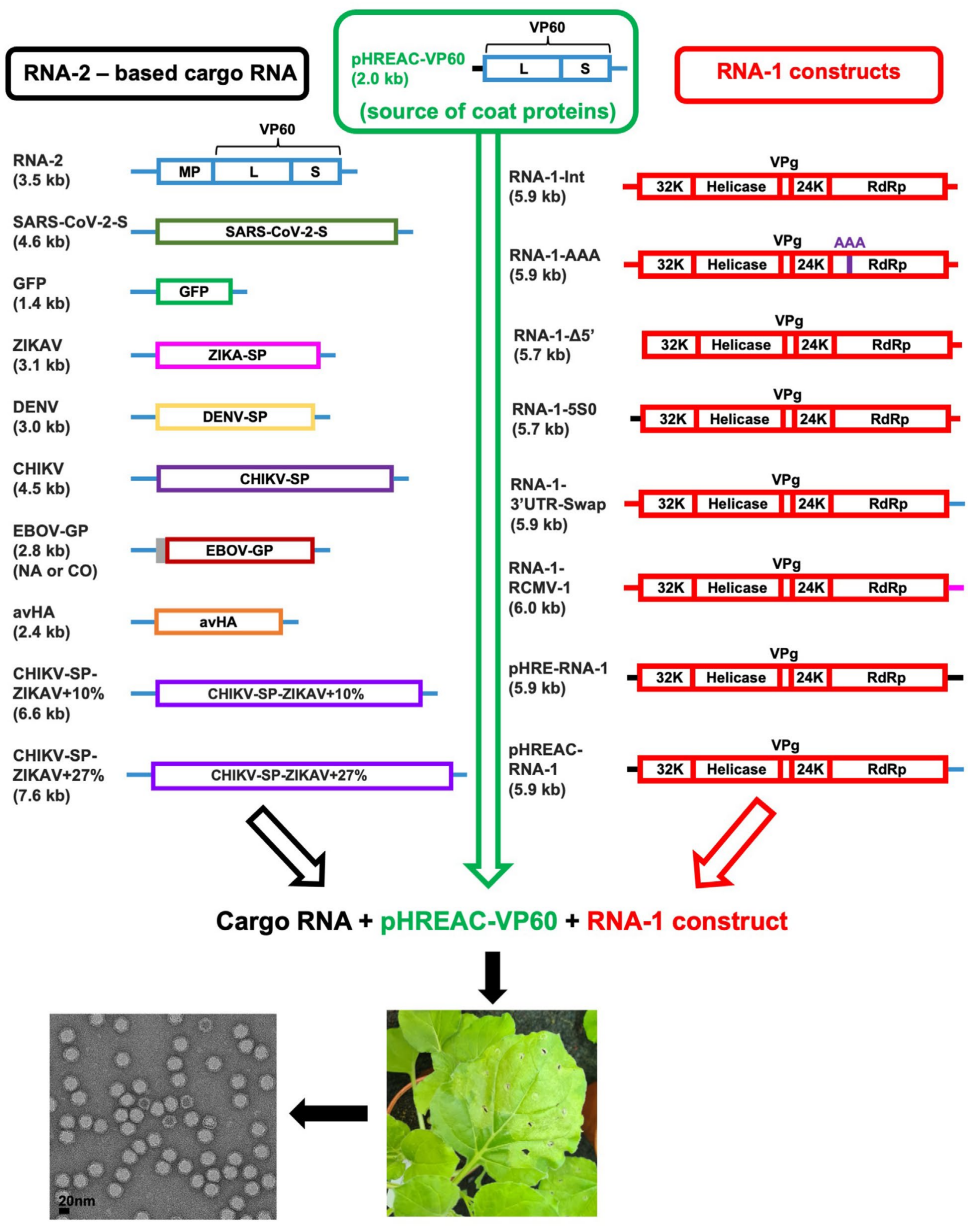
Workflow for the production of virus‐like particles (VLPs) containing cargo RNAs. Diagrams of the constructs used in this study are shown in the upper part of the figure. In each case, *N. benthamiana* leaves are co‐infiltrated with one RNA‐2‐based derivative (left‐hand side) and one RNA‐1 (shown in red; right‐hand side) construct in the presence pHREAC‐VP60 (green, middle) that encodes the precursor of the viral coat proteins, L and S. UTRs (shown as horizontal lines at the start and end of each constructs) are colour coded: Blue for RNA‐2 UTRs, red for RNA‐1 UTRs, black for synthetic UTRs, and pink for RCMV UTRs. The infiltrated *N. benthamiana* leaves (bottom middle) were harvested 6–7 days after infiltration and VLPs were purified from the leaves. The bottom left‐hand panel shows typical VLPs, negatively stained with 2% (w/v) uranyl acetate, with the characteristic appearance of CPMV particles.

For initial experiments to examine whether RNAs in excess of 1.5 kb can be efficiently encapsidated in CPMV particles, the sequence encoding a codon‐optimised version of the entire spike (S) protein of SARS‐CoV‐2 isolate Wuhan‐Hu‐1 (3.9 kb) (Jung et al. 2022) was cloned into pEAQ‐AgeI to give pEAQ‐S. Including the sequence of the 5′ and 3′ UTRs, this plasmid was anticipated to produce an RNA (SARS‐CoV‐2‐S, see Figure 1 and Supporting Information Part 1) of approximately 4.6 kb, which is intermediate in size between CPMV RNA‐1 (6.0 kb) and RNA‐2 (3.5 kb).

**FIGURE 2:**
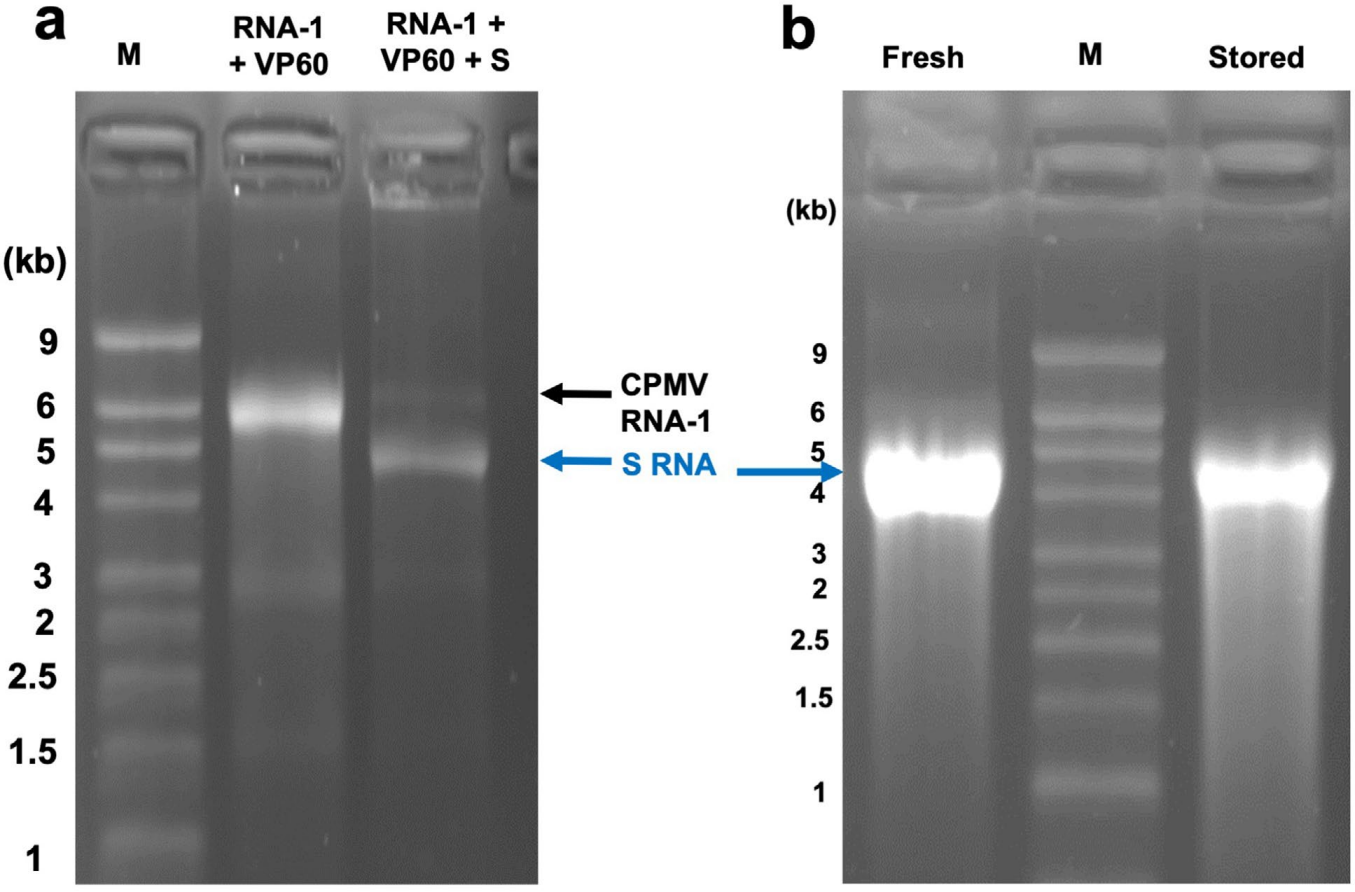
Analysis of RNA encapsidated within VLPs by denaturing agarose gel electrophoresis. (a) RNA extracted from equal amounts of total VLPs produced from co‐expression of CPMV RNA‐1 and VP60 alone (left) or in combination with pEAQ‐S (right). Bands representing packaged CPMV RNA‐1 and SARS‐CoV‐2‐based RNA (S RNA) are indicated by black and blue arrows, respectively. (b) RNA extracted from RNA1 + VP60 + S VLPs, partially separated by CsCl density gradient centrifugation, either immediately after VLP purification (fresh) or after storage of the VLPs at 4°C for 5 months (stored). The position of S RNA is indicated by the blue arrow. Lane M contains RNA size markers.

The capacity of SARS‐CoV‐2‐S RNA to be packaged in *N. benthamiana* was tested by co‐expression of wild‐type CPMV RNA‐1 supplied from the plasmid pEAQ‐RNA‐1‐Int (Kruse et al. 2019) and the CPMV capsid protein precursor VP60 supplied from the plasmid pHREAC‐VP60 (Peyret et al. 2022) with or without pEAQ‐S. Analysis of the RNA content of the resulting particles showed either a single RNA of approximately 6.0 kb (VP60 + RNA‐1) or two bands of approximately 6.0 and 4.6 kb (SARS‐CoV‐2‐S + VP60 + RNA‐1; Figure 2a). This is consistent with self‐replicating RNA‐1 being packaged in both cases and, where present, SARS‐CoV‐2‐S‐encoding RNA also being replicated and packaged. The identity of the 4.6kb product was confirmed by RT‐PCR and northern blot analysis using a probe specific for the S protein. The particles could be further purified by centrifugation through CsCl gradients, which also resulted in partial separation of the two types of particles (Figure 2b). Overall, SARS‐CoV‐2‐S appeared to be packaged more efficiently than RNA‐1, as judged by the intensity of ethidium bromide staining.

To examine whether the encapsidated SARS‐CoV‐2‐S RNA is fully protected from degradation, RNA was extracted from CsCl gradient fractions of VLPs enriched in SARS‐CoV‐2‐S either immediately after fractionation followed by storage at −20°C or after incubation of the VLPs at +4°C for 5 months, in the absence of any preservative or protease inhibitors, prior to RNA extraction. Comparison of the RNAs showed that minimal degradation had occurred on prolonged incubation at 4°C, validating the idea that encapsidation within VLPs stabilises RNA at least as long as 4.6 kb (Figure 2b).

**FIGURE 3:**
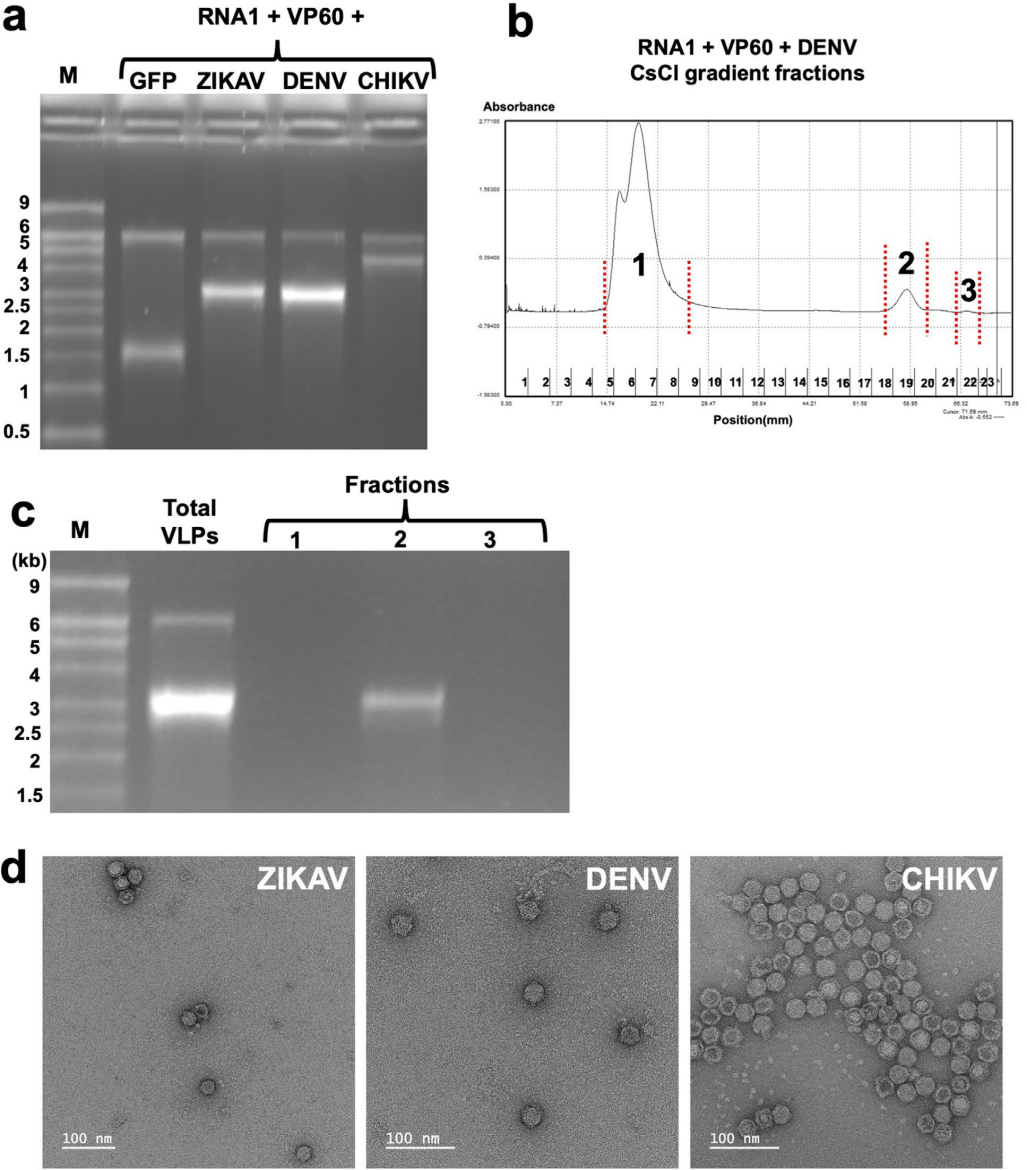
Encapsidation of RNAs of varying lengths and sequences within CPMV VLPs. (a) RNA extracted from equal amounts of total VLPs produced from co‐expression of CPMV RNA‐1 and VP60 alongside an RNA encoding either GFP or a viral antigen, as labelled. (b) Separation of VLPs produced by infiltration with RNA‐1, VP60 and DENV construct on a CsCl density gradient. Fractionation was monitored by absorbance at 280 nm and fractions representing the three peaks (1–3) were pooled. (c) Analysis of the RNA content of the pooled fractions from the density gradient by denaturing agarose gel electrophoresis and staining with ethidium bromide. RNA extracted from the VLP preparation before CsCl centrifugation (total VLPs) was analysed as a control. Lane M contains RNA size markers. (d) Negatively stained images of particles containing cargo RNA as indicated after purification of CsCl gradients and storage for 2 years.

To determine whether SARS‐CoV‐2‐S is an exceptional case, RNAs encoding the structural proteins (SP) from dengue virus (DENV), Zika virus (ZIKAV) and Chikungunya virus (CHIKV) were inserted into pEAQ‐AgeI to give pEAQ‐DENV1‐SP, pEAQ‐ZIKAV‐SP, and pEAQ‐CHIKV‐SP, respectively (see Figure 1 for diagrams and Supporting Information Part 1 for sequences). Each plasmid was agroinfiltrated into *N. benthamiana* leaves in the presence of pHREAC‐VP60 and pEAQ‐RNA1‐Int. In addition, pEAQ‐GFP was similarly co‐infiltrated as a control. Analysis of the RNA contained within equal quantities of purified particles in each case revealed the presence of two RNA molecules, one of 6 kb, corresponding to CPMV RNA‐1 and a second that corresponded to the expected size of the custom RNAs (4.5, 3.0, 3.1 and 1.4 kb for CHIKV, DENV, ZIKAV and GFP respectively—Figure 3a). These results indicate that RNAs with a variety of lengths and sequences can be efficiently encapsidated within CPMV particles. Attempts to separate the RNA‐1 containing particles from those containing the modified RNA‐2 by centrifugation through CsCl gradients were only partially successful, with a reasonable separation being obtained with the DENV construct (Figure 3b,c), which differs from RNA‐1 by 3 kb. Particles enriched for those containing the cargo RNA by CsCl gradient centrifugation retained the typical morphology of CPMV particles when stored for two years at 4°C (Figure 3d).

### 2.2 Codon‐Optimisation Is Not Required for Efficient RNA Encapsidation

**FIGURE 4:**
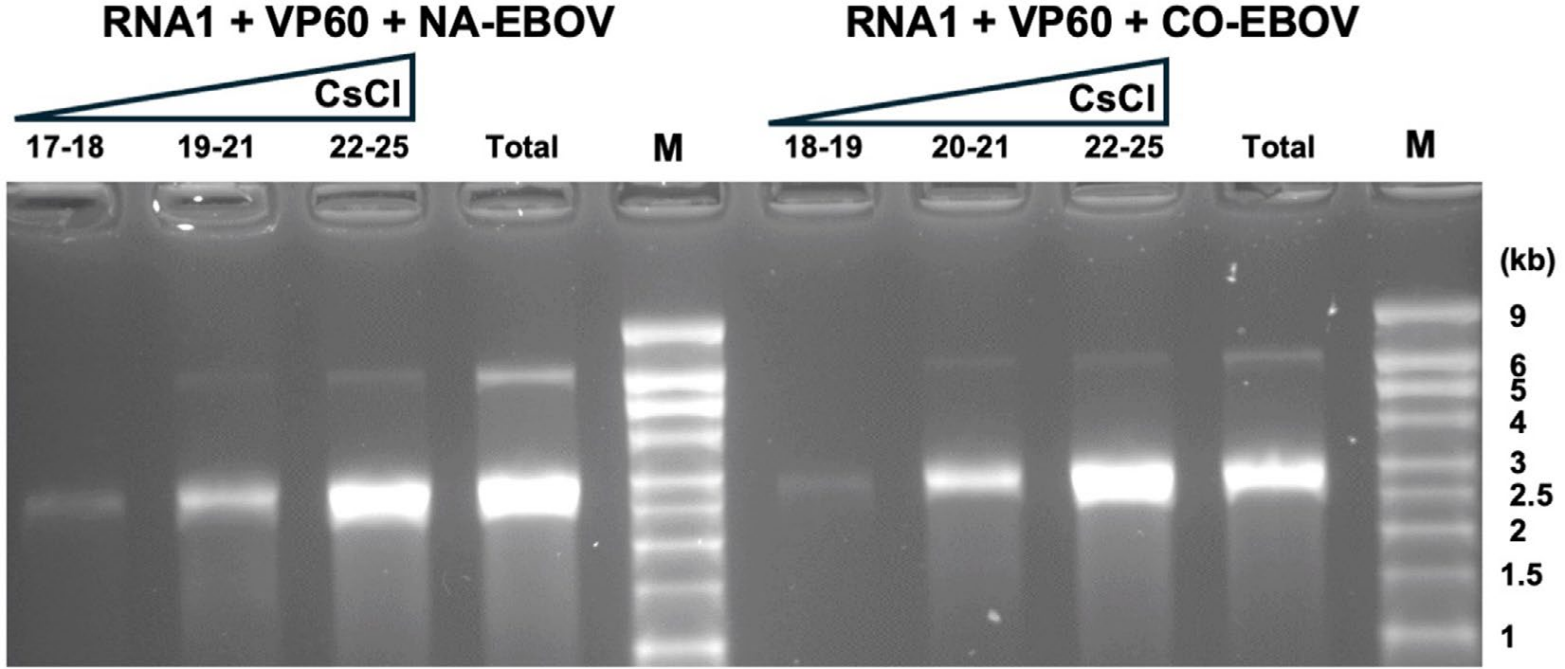
Effect of codon optimisation of packaging of cargo RNA. RNA was extracted from equal amounts of either VLPs before CsCl gradient centrifugation (lanes marked total) or VLPs from different CsCl gradient pooled fractions. The VLPs were produced by co‐expression of CPMV RNA‐1 and VP60 alongside an RNA encoding either native (NA; left‐hand side) or plant codon‐optimised (CO; right‐hand side) sequence encoding ebolavirus GP. The RNA was analysed by electrophoresis on denaturing agarose gels, followed by staining with ethidium bromide. Lane M contains RNA size markers.

The RNA sequences examined above were all codon‐optimised for *N. benthamiana*. However, although this may be optimal for replication and encapsidation in plants, it may not be so if the particles are used to deliver RNA designed to be translated within mammalian cells (Roberts et al. 2024). To investigate how the encapsidation of mammalian viral sequences with native codon usage compares with that of plant codon‐optimised sequences, two constructs were created which both contain the coding sequence from the full‐length Ebola virus (EBOV) glycoprotein (GP) with either a wild‐type codon usage or codon‐optimised for *N. benthamiana*. As these constructs were specifically designed for translation in a mammalian cell context, the coding sequences were preceded by a 48bp sequence from the human α‐globin 5ʹUTR and a mammalian Kozak consensus sequence (see Supporting Information Part 1 for sequences). The two plasmids, containing the native (pMOD‐EAQ‐EBOV‐GP‐NA) or codon‐optimised (pMOD‐EAQ‐EBOV‐GP‐CO) versions, were separately infiltrated into *N. benthamiana* leaves in the presence of pHREAC‐VP60 and pEAQ‐RNA‐1‐Int. Analysis of the RNA extracted from equal amounts (6.0 mg) of total purified particles revealed the presence of both RNA‐1 (6 kb) and additional RNA of approximately 2.8 kb, consistent with the size of the RNA containing the sequence of EBOV GP (Figure 4). The total amount of RNA obtained was 1.7 and 1.2 μg for pMOD‐EAQ‐EBOV‐GP‐NA and pMOD‐EAQ‐EBOV‐GP‐CO, respectively. This shows that RNA containing the native EBOV GP sequence was encapsidated at least as efficiently as the codon‐optimised version. The custom RNA‐containing particles could be partially separated from RNA‐1 by centrifugation through CsCl gradients (Figure 4).

### 2.3 A Non‐Replicating Version of RNA‐1 Can Support RNA‐2 Replication

Previous work had suggested that the presence of a replication‐competent version of CPMV RNA‐1 is necessary not only for its own replication but also for the replication of RNA‐2 (Kruse et al. 2019; Liu et al. 2004). As a result, experiments requiring a replicating RNA, such as the encapsidation experiments described above, have relied on supplying a version of RNA‐1 capable of replicating itself. This inevitably results in the encapsidation of RNA‐1, as well as cargo RNA flanked by UTRs of RNA‐2, into virus‐derived particles.

The conclusion that replicating RNA‐1 is required for RNA‐2 replication was based on the phenotype of an RNA‐1 mutant, RNA‐1‐32E (Kruse et al. 2019; Liu et al. 2004), which was constructed to lack the entire 5ʹUTR while retaining a functional ORF. However, resequencing the entire RNA‐1 region of the plasmid pBinP32E (the expression vector used to express RNA‐1‐32E in plants) revealed that, in addition to lacking the 5ʹUTR, the ORF had a mutation that changed the amino acid at position 325 on the polyprotein from alanine to aspartic acid (A325D), as well as two silent mutations. Since this change is immediately upstream of the Q/S cleavage site used to separate the 32 kDa protease cofactor from the 58 kDa helicase (Wellink et al. 1986), it may prevent correct processing of the RNA‐1 polyprotein, thereby abolishing its ability to form a functional replication complex. Thus, it is probable that it is this, rather than the lack of the 5ʹUTR, that prevents RNA‐1‐32E from supporting RNA‐2 replication.

**FIGURE 5:**
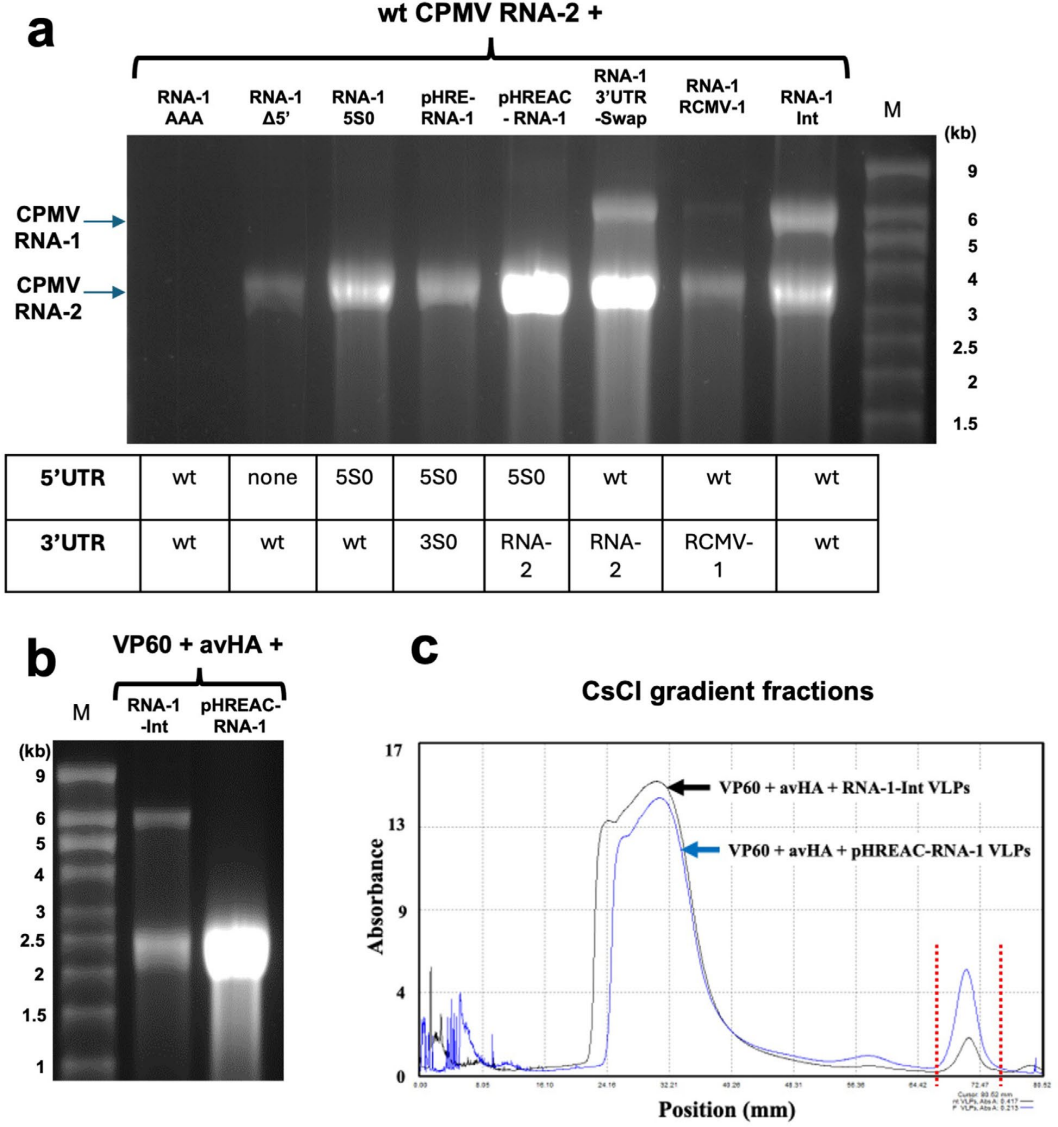
Effect of using different RNA‐1 constructs on the amount of RNA‐2 that is encapsidated. (a) Denaturing agarose gel of RNA extracted from equal amounts of total purified particles produced by co‐expressing wt RNA‐2 and a version of CPMV RNA‐1 as indicated. The UTR combination for each version of RNA‐1 used is shown below the corresponding lane in the gel. (b) Effect of using a replicating (RNA‐1‐Int) or non‐replicating (pHREAC‐RNA‐1) version of RNA‐1 on the encapsidation of the avHA construct. RNA was extracted from equal amounts of VLPs and analysed by denaturing agarose gel electrophoresis. Lane M contains RNA size markers (c) CsCl density gradient A280 profiles of VLPs produced by replicating (black line) and non‐replicating (blue line) of RNA‐1 as described in (b). The two profiles have been slightly offset to allow them to be easily distinguished. The peak between the dotted red lines represents the region of the gradient containing the encapsidated avHA RNA.

To assess whether it is possible to achieve RNA‐2 replication in the absence of RNA‐1 replication, modified pEAQ‐RNA‐1‐Int‐based constructs, which do not have the A325D mutation, were created which either lack the 5′ UTR or have the 5′ and 3′UTRs replaced by other comovirus or synthetic UTRs (see Figure 1 for diagrams and the Supporting Information Part 2 for complete sequences). Each of these modified RNA‐1 constructs was co‐inoculated with pEAQ‐RNA‐2 (encoding wild‐type (wt) CPMV RNA‐2) into *N. benthamiana*. As controls, pEAQ‐RNA‐1‐Int or pEAQ‐RNA‐1‐Int‐AAA, which encodes an inactive replicase (Kruse et al. 2019), were also co‐inoculated with pEAQ‐RNA‐2. Analysis of RNA extracted from equal amounts of purified particles showed that, as expected, pEAQ‐RNA‐1‐Int supported the encapsidation of both RNA‐1 and RNA‐2 while pEAQ‐RNA‐1‐Int‐AAA supported the encapsidation of neither (Figure 5a). In cases where the RNA‐1 construct had the wt 5ʹUTR from RNA‐1 and a 3′UTR from either CPMV RNA‐2 (RNA‐1‐3′UTR‐Swap) or RCMV RNA‐1 (RNA‐1‐RCMV‐1), the modified RNA‐1 was able to replicate both itself and CPMV RNA‐2, though the construct with the RCMV RNA‐1 3′UTR appeared to direct about half as much RNA packaging as the constructs which contained the CPMV 3′UTR. By contrast, when the RNA‐1 5′UTR was either deleted (RNA‐1‐Δ5′) or replaced by the synthetic 5S0 5′ UTR (RNA‐1‐5S0; Peyret et al. 2019), RNA‐1 was no longer capable of self‐replication but could still support the replication of RNA‐2 (Figure 5a). This was also the case when both the 5′ and 3′ UTRs of RNA‐1 were replaced with synthetic UTRs (pHRE‐RNA‐1, containing 5S0 and 3S0 synthetic UTRs described in Peyret et al. 2019). The amount of RNA‐2 encapsidated varied but was highest when the RNA‐1 coding region was placed between the 5S0 5ʹUTR and the RNA‐2 3′UTR (expressed by the plasmid pHREAC‐RNA‐1). Indeed, the total amount of packaged RNA was found to be similar between equal amounts of VLPs produced using pEAQ‐RNA‐1‐Int versus those produced using pHREAC‐RNA‐1, but VLPs produced with the latter did not package RNA‐1, so the amount of RNA‐2 packaged was clearly far higher than in VLPs produced with wt RNA‐1. This combination of UTRs (synthetic 5S0 5′UTR and wt RNA‐2 3′UTR) is the same as that present in the pHREAC overexpression vector, which was specifically designed for efficient translation (Peyret et al. 2019), and which was found to direct more efficient translation than the pHRE vector, also used here. These results strongly suggest that the translatability of RNA‐1 (i.e., presumably the amount of RNA‐1‐encoded replicase complex) governs the level of RNA‐2 replication/encapsidation.

### 2.4 Encapsidation of a Modified RNA‐2 in the Absence of RNA‐1 Replication

To test whether pHREAC‐RNA‐1 can support the replication and encapsidation of an RNA‐2‐derived cargo RNA, a plasmid carrying the sequence of a plant codon‐optimised version of avian influenza virus haemagglutinin (HA) flanked by the CPMV RNA‐2 UTRs (pEAQ‐avHA) was constructed. The RNA produced by this construct is anticipated to be 2.4 kb. This construct was used to inoculate *N. benthamiana* in the presence of pHREAC‐VP60 and either pEAQ‐RNA‐1‐Int or pHREAC‐RNA‐1. RNA extracted from equivalent amounts of purified particles was analysed by denaturing agarose gel electrophoresis. In the case of the VLPs produced by inoculation with pEAQ‐RNA‐1‐Int, two bands could be resolved: one of 6 kb, equivalent to RNA‐1, and the other of the size expected to be produced by the pEAQ‐avHA construct (2.4 kb; Figure 5b). By contrast, when pHREAC‐RNA‐1 was used, only a single band of 2.4 kb was detected, confirming that pHREAC‐RNA‐1 is efficient at directing the replication of the cargo RNA construct but cannot replicate itself. Quantitation of the RNA isolated from equivalent amounts of VLP indicated that those produced by pHREAC‐RNA‐1 contained approximately 4.3 times as much RNA as those produced by pEAQ‐RNA‐1‐Int (Figure 5b). Analysis of the composition of the VLP preparations by CsCl density gradients confirmed that pHREAC‐RNA‐1 produced a far higher proportion of particles migrating towards the bottom of the gradient (consisting of a mixture of avHA RNA and RNA‐1 when pEAQ‐RNA‐1 was used and just avHA when pHREAC‐RNA‐1 was used) than when pEAQ‐RNA‐1‐Int was used to drive replication (Figure 5c).

### 2.5 Determining the Maximum Length of RNA That Can Be Incorporated Into CPMV Particles

**FIGURE 6:**
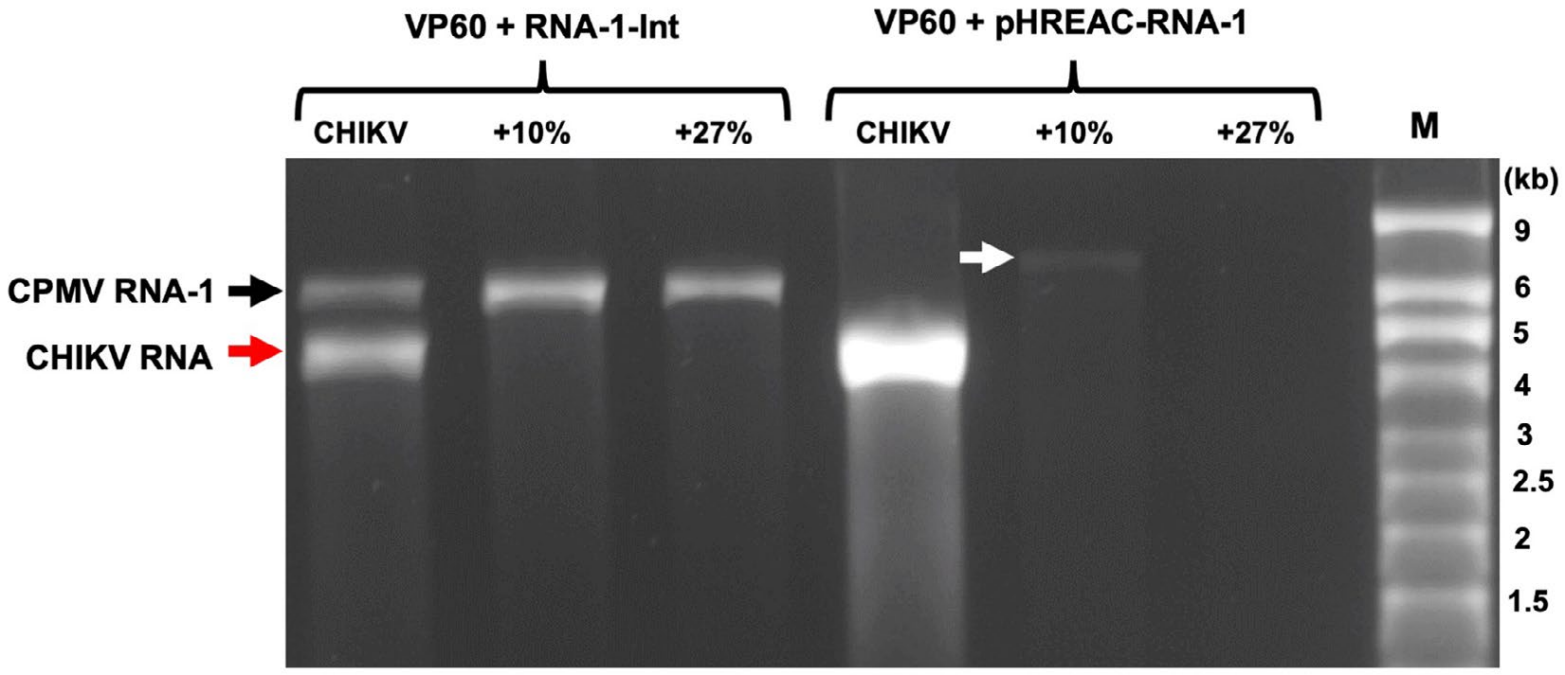
Analysis of the encapsidation of cargo RNAs greater in length than CPMV RNA‐1. The replication of the RNAs was achieved by co‐infiltration with either a replicating (RNA‐1‐Int) or non‐replicating (pHREAC‐RNA‐1) version of RNA‐1. RNA was extracted from equal amounts of VLPs and analysed by denaturing agarose gel electrophoresis followed by ethidium bromide staining. The position of CPMV RNA‐1 is indicated by a black arrow while CHIKV RNA is indicated by a red arrow. The position of cargo RNA 10% longer than CPMV RNA‐1 (only visible when pHREAC RNA‐1 was used) is indicated by the white arrow. Lane M contains RNA size markers.

From the data presented above, it is clear that custom RNA molecules of a variety of sizes and sequences (all flanked by the UTRs of CPMV RNA‐2) can be successfully incorporated into CPMV particles. In some cases (e.g., pEAQ‐*HT*‐S and pEAQ‐CHIKV‐SP), the cargo RNA is actually larger than wt CPMV RNA‐2 (3.5 kb). However, in no case did the size of the cargo RNA construct exceed that of RNA‐1 (6 kb). To determine whether it is possible to package RNA molecules larger than RNA‐1, two constructs based on pEAQ‐CHIKV‐SP were designed that contained additional sequence from ZIKAV‐SP downstream of the CHIKV‐SP ORF (Figure 1). These sequences increased the size of the RNAs from 4.5 kb (pEAQ‐CHIKV‐SP) to 6.6 kb (pEAQ‐CHIKV‐SP‐ZIKAV+10%) and 7.6 kb (pEAQ‐CHIKV‐SP‐ZIKAV+27%), which represents an increase in size over RNA‐1 by 10% and 27%, respectively. To investigate whether these larger RNAs can still be packaged, pEAQ‐CHIKV‐SP, pEAQ‐CHIKV‐SP‐ZIKAV+10% and pEAQ‐CHIKV‐SP‐ZIKAV+27% were inoculated into *N. benthamiana* leaves in the presence of pHREAC‐VP60 and either pEAQ‐RNA‐1‐Int or pHREAC‐RNA‐1. Denaturing agarose gel analysis of RNA isolated from the equivalent mass of VLPs showed the presence of RNA‐1 in all the samples co‐inoculated with pEAQ‐RNA‐1‐Int and a clear band of 4.5 kb in the sample inoculated with pEAQ‐CHIKV‐SP; however, no obvious band larger than RNA‐1 could be seen in the samples inoculated with pEAQ‐CHIKV‐SP‐ZIKAV+10% or pEAQ‐CHIKV‐SP‐ZIKAV+27% (Figure 6). This suggests that RNAs bigger than RNA‐1 are inefficiently packaged, if at all, though it is possible that bands representing larger RNAs may be masked by the more abundant RNA‐1. As expected, there was no RNA‐1 present in the samples produced by inoculation with pHREAC‐RNA‐1. A band of 4.5 kb could be observed in the sample produced by co‐inoculation with pEAQ‐CHIKV‐SP and pHREAC‐RNA‐1; this band had a considerably greater intensity than that found in the sample produced by co‐inoculation of pEAQ‐CHIKV‐SP and pEAQ‐RNA‐1‐Int, consistent with the previous data obtained with pEAQ‐avHA (Figure 5b). In the case of VLPs produced by co‐inoculation with pEAQ‐CHIKV‐SP‐ZIKAV+10% and pHREAC‐RNA‐1, a distinct but faint band representing an RNA of 6.6 kb could be seen, together with some faster migrating material. When the size of RNA‐2 was increased further (pEAQ‐CHIKV‐SP‐ZIKAV+27%), no distinct band of 7.6 kb could be discerned; though, again, faster migrating RNA of variable size could be seen (Figure 6). These results suggest that it is possible to incorporate custom RNA molecules somewhat larger than RNA‐1, but the efficiency of the process is severely compromised.

## 3 Discussion

The results presented here show that RNAs of varying size and sequence can be successfully incorporated into CPMV particles, even if the size of the RNA exceeds that of wt RNA‐2. This means that RNAs containing substantial open reading frames can be incorporated for potential delivery to cells. To this end, we have focussed on the incorporation of RNAs encoding the structural proteins from a variety of enveloped viruses for which there is an ongoing need to develop new vaccines. Since the encapsidated RNAs are protected from degradation during storage for prolonged periods at +4°C, the technology reported here could form the basis of a novel delivery system for RNA vaccines that do not require storage at ultra‐low temperatures. The results with EBOV also demonstrate that codon optimisation for plants is not required for encapsidation, an important consideration if the encapsidated RNA is to be efficiently translated when delivered to mammalian cells.

A disadvantage of the method originally employed to package custom RNA constructs is that it relied on co‐infiltration with a replication‐competent version of RNA‐1. This was believed to be necessary as previous data has shown that a mutant RNA‐1 lacking the 5ʹUTR (RNA‐1‐32E) could support neither its own replication nor the replication of RNA‐2 despite it containing an apparently intact open reading frame (Kruse et al. 2019; Liu et al. 2004). However, the finding that it is the A325D mutation in the RNA‐1‐32E polyprotein, rather than the lack of the RNA‐1 5ʹUTR that is responsible for the inability of RNA‐1‐32E to support RNA‐2 replication, has enabled us to develop a version of RNA‐1 (pHREAC‐RNA‐1) that cannot self‐replicate but can replicate constructs flanked by the UTRs of RNA‐2. The efficiency of this RNA‐1 at replicating cargo RNA appears to depend on the efficiency with which it is translated, indicating that the greater the level of the replication‐associated proteins, the greater amount of cargo RNA replication that can be achieved. This is clearly advantageous in terms of the amount of cargo RNA‐containing particles that can be obtained, with an approximately fivefold increase in the amount of VLPs containing avHA RNA being obtained when pHREAC‐RNA‐1, rather than pEAQ‐RNA‐1‐Int, is used.

As well as allowing greater levels of VLPs containing custom RNA molecules to be obtained, using a non‐self‐replicating (and therefore non‐packaging) version of RNA‐1 prevents these from being contaminated with RNA‐1‐containing particles, which are difficult to separate physically. The presence of RNA‐1‐containing particles can potentially complicate the interpretation of results when VLPs are delivered to mammalian cells since it could theoretically lead to the further replication of the cargo constructs in the cells to which the VLPs have been delivered. Though there is no evidence that this actually occurs, using a version of RNA‐1 that is not packaged eliminates that possibility. This may have advantages in terms of biosafety over recently developed replicative systems for vaccination such as Kostaive (Hakariya and Ohashi 2024; Hồ et al. 2024). However, despite increasing the levels of VLPs containing custom RNA constructs and eliminating the production of RNA‐1‐containing VLPs, unfractionated VLP preparations still contain substantial amounts of empty VLPs (Figure 3). Though these do not appear to interfere with the delivery of RNA to mammalian cells (Roberts et al. 2024), for certain applications it may be advantageous to remove them. This can be achieved relatively easily by CsCl density gradient centrifugation since empty VLPs are well separated from those containing RNA.

The use of pHREAC‐RNA‐1 has allowed us to assess the ability of CPMV VLPs to encapsidate RNAs longer than RNA‐1 (6 kb). The results suggest that encapsidation efficiency declines dramatically once the size of cargo RNA is increased beyond 6 kb. When the size is increased by 10% to 6.6 kb, some full‐length material can be extracted, but in very low yield; when the size is increased by 27% to 7.6 kb, no full‐length material could be recovered. However, in both cases, smaller RNAs could be isolated. These results suggest that 6 kb, the size of RNA‐1, represents the maximum length of RNA that can be efficiently incorporated into CPMV particles, though this limit can be exceeded by 10% with a dramatic yield penalty. The situation is similar to that found with poliovirus, where the genome size can be increased by up to 15% while retaining infectivity (Andino et al. 1994; Ansardi et al. 1996; Molla et al. 1992). Such a limit makes virological sense, since there would be little evolutionary advantage in maintaining a capsid capable of incorporating larger RNAs.

A particular feature of the CPMV‐based packaging system is that it appears to be very flexible and able to package RNAs of a variety of lengths and sequences. The only common feature of the custom RNA constructs is the preservation of the 5′ and 3′ UTRs that are necessary for replication. However, these are likely to aid translation of the cargo RNA when introduced into heterologous cells rather than having any deleterious effect (Roberts et al. 2024). Though it is possible that these sequences contain signals needed for specific packaging of RNAs into viral particles, previous studies have suggested that it is the act of replication itself that targets the CPMV RNAs for encapsidation rather than the presence of any specific sequences (Kruse et al. 2019; Peyret et al. 2025). The fact that RNAs from differing origins, including GFP (Kruse et al. 2019), a synthetic sequence encompassing primer binding sites from SARS‐CoV‐2 (Peyret et al. 2022) as well as the sequences described here, can all be efficiently packaged argues against the presence of cryptic dispersed packaging signals on the genomic RNAs, as suggested in the case of picornaviruses (Shakeel et al. 2017). However, it is possible that the viral‐derived sequences used in the current study may have an intrinsic ability to be condensed for packaging, which assists in their incorporation into CPMV particles. Nevertheless, the fact that such sequences do not get packaged unless they are replicated (Kruse et al. 2019) demonstrates that putative packaging signals on the UTRs, even if they exist, are not sufficient to direct packaging into CPMV VLPs on their own. Further studies investigating the encapsidation of RNAs of differing (non‐viral) origins and with differing secondary structures, along with further studies on the UTRs of RNA‐2, will be needed to determine whether such packaging signals exist on these UTR sequences.

There are several issues that will need to be addressed before clinical deployment of the CPMV‐based technology described here. For example, though the electrophoresis of RNA extracted from VLPs is an effective way of demonstrating the intactness of the encapsidated designer RNA, it does not provide information as to whether any other RNAs are also encapsidated at low levels. Examination of the levels and identity of such RNAs would require next‐generation sequencing of the totality of the RNAs within the particles using approaches similar to those described by Kotta‐Loizou et al. (2019) and Routh et al. (2012) which, though extremely interesting, are beyond the scope of the current study. Likewise, the current method of separating RNA‐containing from empty VLPs involves centrifugation through CsCl gradients, a process that may be difficult to scale up. If removal of empty VLPs is desired, it may be necessary to develop alternative methods, such as relying on differential stability of the empty and RNA‐containing particles (Da Poian et al. 2002), to achieve this.

## 4 Materials and Methods

### 4.1 Plasmids

Diagrams of all constructs used in this study are shown in Figure 1, and complete sequences of the inserts within the various plasmids are available in the Supporting Information.

All replication‐competent RNA‐2‐derived constructs designed for custom RNA packaging were based on pEAQ‐GFP (Kruse et al. 2019) and a derivative thereof into which an AgeI site had been introduced at the end of the 5′ UTR (pEAQ‐AgeI). This allowed cloning of the sequences of interest (shown in Supporting Information Part 1) to be designed as AgeI‐XhoI restriction fragments and inserted between the wild‐type 5′ and 3′ UTRs of CPMV RNA‐2, which are themselves flanked by a 35S promoter and *Nos* terminator (Figure 1). For RNA sequences intended for protein expression, care was taken to ensure that the start codon of the coding sequence was always positioned in‐frame with the upstream AUG at position 161 in the CPMV RNA‐2 5′ UTR (Cañizares et al. 2006; Kruse et al. 2019).

Sequences encoding the Spike (S) proteins of SARS‐CoV‐2 (isolate Wuhan‐Hu‐1, GenBank: OK413878), the structural proteins of Dengue virus (DENV, GenBank: KM204119.1), Zika virus (ZIKAV; GenBank KU321639) and Chikungunya virus (CHIKV; GenBank: DQ443544.2) and the haemagglutinin gene of Influenza A virus (avHA) (A/chicken/Egypt/F71/2022 (H5N1), GenBank: OQ380623.1), all codon‐optimised for expression in *N. benthamiana* , were inserted into pEAQ‐AgeI to give pEAQ‐S, pEAQ‐DENV1‐SP, pEAQ‐ZIKAV‐SP, pEAQ‐CHIKV‐SP and pEAQ‐avHA, respectively. Two versions of the sequence encoding the full‐length Ebolavirus (EBOV) glycoprotein (GP, GenBank: AAD14585.1), with either wild‐type codon usage or codon‐optimised for *N. benthamiana*, were also synthesised and inserted into pEAQ‐AgeI to give pMOD‐EAQ‐EBOV‐GP‐NA and pMOD‐EAQ‐EBOV‐GP‐CO, respectively. All the synthetic sequences described above were synthesised by GeneArt (ThermoFisher Scientific) and the cloned construct sequences were verified by DNA sequencing (Eurofins).

To increase the length of the sequences within the pEAQ plasmids beyond that of RNA‐1, hybrid genes were constructed by fusing the full‐length of insert of pEAQ‐CHIKV‐SP to portions of pEAQ‐ZIKAV‐SP via overlap‐extension PCR to create pEAQ‐CHIKV‐SP‐ZIKAV+10% and pEAQ‐CHIKV‐SP‐ZIKAV+27%.

Plasmids containing versions of CPMV RNA‐1 with differing 5′ and 3′UTRs were all based on pEAQ‐RNA1‐Int (Kruse et al. 2019) which encodes wild‐type RNA‐1 (though introns are present in the DNA construct). Most RNA‐1 derivatives were made by overlap‐extension PCR on the PacI/AscI restriction fragment of pEAQ‐RNA‐1‐Int (which contains the entire expression cassette for RNA‐1), and the modified PacI/AscI restriction fragments were used to replace the original PacI/AscI fragment in pEAQ‐RNA‐1‐int. The two exceptions were pHRE‐RNA‐1 and pHREAC‐RNA‐1; in these cases, the RNA‐1 ORF from pEAQ‐RNA‐1‐Int was amplified by PCR and inserted, respectively, into the BsaI restriction sites of vectors pHRE or pHREAC (Peyret et al. 2019) so as to place the coding region of RNA‐1 between the UTRs already present on these plasmids (see Figure 1 for diagrams and complete sequences in and Supporting Information Part 2).

### 4.2 Production and Purification of CPMV Particles

For expression in plants, all plasmids were transformed into *Agrobacterium tumefaciens* strain LBA4404 and used to agroinfiltrate *N. benthamiana* leaves as previously described (Kruse et al. 2019; Peyret et al. 2022; Peyret and Lomonossoff 2022). To encapsidate replicating RNA, the plasmids encoding the target sequences within pEAQ‐AgeI were co‐infiltrated with pHREAC‐VP60 (Peyret et al. 2022) to supply the CPMV capsid protein precursor and either pEAQ‐RNA‐1‐Int (Kruse et al. 2019) or plasmids containing modified versions of CPMV RNA‐1 (Figure 1).

CPMV particles were extracted and purified 6–7 days post‐infiltration based on a previously published protocol (Peyret and Lomonossoff 2022). Briefly, infiltrated leaves were blended in extraction buffer, the extracts filtered through Miracloth and clarified by centrifugation at 13 000 × ***g*** for 20 min. VLPs were precipitated overnight at 4°C with stirring by adding PEG 6000 to 4% (w/v) and NaCl to 0.2 M (final concentrations), and collected by centrifugation at 13 000 × ***g*** for 20 min. The pelleted particles were resuspended in 10 mM sodium phosphate (pH 7.2) and either pelleted by ultracentrifugation (2 h 30 min at 118 000 × ***g***) to obtain total particles, or mixed with solid CsCl to a final concentration of 41% (w/v) and centrifuged overnight (15–24 h) at ~280 000 × ***g*** in a swing‐out Sorvall TH641 ultracentrifuge rotor so as to obtain a self‐forming density gradient in which the particles are separated according to their RNA content. In some cases, total VLPs, first obtained by pelleting, were subsequently subjected to CsCl gradient ultracentrifugation.

Pelleted total VLPs were resuspended in a small volume of 10 mM sodium phosphate (pH 7.2) while CsCl gradients were fractionated on a BioComp piston fractionator and selected fractions were buffer‐exchanged against 10 mM sodium phosphate (pH 7.2). Purified particles were then quantified using a bicinchoninic acid (BCA) protein quantification assay kit (Pierce) with a bovine serum albumin (BSA) standard curve as per manufacturer's instructions. In all cases, the quantification was verified by analysing equal protein amounts of each sample by SDS‐PAGE (NuPAGE, Life Technologies) and staining with Instant Blue stain (Expedeon). Purified virions/VLPs were stored at 4°C–8°C. If long‐term storage was anticipated, sodium azide was added to a final concentration of 3 mM to avoid microbial contamination.

### 4.3 Analysis of Encapsidated RNA

RNA extraction was carried out on equal amounts of particles as determined by protein quantification described above. Typically, 0.5–1 mg of total VLPs were used for RNA extraction from preparations of total particles (obtained by pelleting) whereas only 0.05 mg of VLPs were used for RNA extractions of CsCl‐fractionated particles. RNA was extracted from purified particles as described in Kruse et al. (2019): briefly, particles were first treated with micrococcal nuclease to digest any non‐packaged nucleic acid, then three successive rounds of phenol:chloroform extractions were performed on each sample, followed by a chloroform‐only extraction. The final aqueous phase was used for RNA precipitation overnight at −20°C using lithium chloride at a final concentration of 2 M. RNA precipitates were centrifuged at top speed in a microcentrifuge (~16 000 × ***g***) for 30–40 min at 8°C, supernatant removed, pellets washed in ice‐cold 70% (v/v) ethanol, centrifuged again for 20 min at 8°C, then supernatant was removed again and pellets air‐dried on the bench for 10 min before resuspending in a small volume (typically 10 μL) of DEPC‐treated nuclease‐free water. Purified RNA was then quantified by measuring the A260 using a nanodrop spectrophotometer (Thermo‐Fisher).

Purified RNA was analysed by electrophoresis on formaldehyde‐containing denaturing agarose gels with each sample prepared in the presence of ethidium bromide, including the RNA ladder (Millennium RNA marker ladder, Ambion). For each gel, the amount of RNA loaded per sample corresponds to that extracted from the same quantity of particles (as determined by protein quantification), such that the quantity of RNA seen from each sample reflects the relative packaging efficiency.

## Author Contributions

H.P., G.P.L.: conceptualisation. H.P., S.N.S., Y.M., J.‐W.J., K.S.: methodology. All authors: validation, writing – review and editing, and formal analysis. H.P., S.N.S., Y.M., J.‐W.J., K.S.: investigation. G.P.L., H.P.: resources. H.P., S.N.S., Y.M., J.‐W.J., K.S.: data curation. H.P., G.P.L.: writing – original draft preparation. G.P.L.: supervision, project administration. G.P.L., H.P., J.‐W.J.: funding acquisition. All authors have read and agreed to the published version of the manuscript.

## Disclosure

A patent entitled “VLP‐scaffold based binding proteins” (W02025061726_A1) related to the work described in this manuscript has been filed.

## Conflicts of Interest

The authors declare no conflicts of interest.

## Supporting information


**Data S1:** pbi70294‐sup‐0001‐supinfo.docx.

## Data Availability

The data that supports the findings of this study are available in the Supporting Information of this article.
