# Supplementary material for: Nicotiana benthamiana as a Source of Cowpea Mosaic Virus‐Derived Particles That Specifically Package Designer RNAs
Source: Plant Biotechnol J. 2025 Aug 6;24(1):159–70. doi: 10.1111/pbi.70294 (PMC12854886; doi:10.1111/pbi.70294)
Supplement: Supplementary file 1 — Data S1: pbi70294‐sup‐0001‐supinfo.docx. [file PBI-24-159-s001.docx]

# Supplementary Information

**Supplementary Part 1**: Sequences of the CPMV RNA-2 - based cargo RNA molecules packaged inside CPMV VLPs described in this manuscript and shown in Figure 1. The 5’ and 3’ UTR sequences of CPMV RNA-2 are highlighted in yellow, cargo sequences of interest are highlighted in blue (protein coding sequence of CPMV RNA-2 is not highlighted). Age1 and Xho1 restriction sites are in bold and underlined. Human α-globin 5’UTR highlighted in pink. Note the RNA sequences indicated here are based on sequencing of their respective expression plasmids.

**>CPMV RNA-2 (encoded by plasmid pEAQ-RNA-2)**

uauuaaaaucuuaauagguuuugauaaaagcgaacguggggaaacccgaaccaaaccuucuucuaaauucucucucaucucucuuaaagcaaacuucucucuugucuuucuugcaugagcgaucuucaacguugucagaucgugcuucggcaccaguacaauguuuucuuucacugaagcgaaaucaaagaucucuuuguggacacguagugcggcgccauuaaauaacguguacuuguccuauucuugucgguguggucuugggaaaagaaagcuugcuggaggcugcuguucagccccauacauuacuuguuacgauucugcugacuuucggcgggugcaauaucucuacuucugcuugacgagguauuguugccuguacuucuuucuucuucuucuugcugauugguucuauaagaaaucuaguauuuucuuugaaacagaguuuucccgugguuuucgaacuuggagaaagauuguuaagcuucuguauauucugcccaaauuugaaauggaaagcauuaugagccgugguauuccuucaggaauuuuggaggaaaaagcuauucaguucaaacgugccaaagaagggaauaaacccuugaaggaugagauucccaagccugaggauauguaugugucucacacuucuaaauggaaugugcucagaaaaaugagccaaaagacuguggaucuuuccaaagcagcugcugggaugggauucaucaauaagcauaugcuuacgggcaacaucuuggcacaaccaacaacagucuuggauauucccgucacaaaggauaaaacacuugcgauggccagugauuuuauucguaaggagaaucucaagacuucugccauucacauuggagcaauugagauuauuauccagagcuuugcuuccccugaaagugauuugaugggaggcuuuuugcuuguggauucuuuacacacugauacagcuaaugcuauucguagcauuuuuguugcuccaaugcggggaggaagaccagucagaguggugaccuucccaaauacacuggcaccuguaucaugugaucugaacaauagauucaagcucauuugcucauugccaaacugugauauuguccaggguagccaaguagcagaagugaguguaaauguugcaggaugugcuacuuccauagagaaaucucacaccccuucccaauuguauacagaggaauuugaaaaggagggugcuguuguuguagaauacuuaggcagacagaccuauugugcucagccuagcaauuuacccacagaagaaaaacuucggucccuuaaguuugacuuucauguugaacaaccaaguguccugaaguuauccaauuccugcaaugcgcacuuugucaagggagaaaguuugaaauacucuauuucuggcaaagaagcagaaaaccaugcaguucaugcuacuguggucucucgagaaggggcuucugcggcacccaagcaauaugauccuauuuugggacgggugcuggauccacgaaaugggaauguggcuuuuccacaaauggagcaaaacuuguuugcccuuucuuuggaugauacaagcucaguucgugguucuuugcuugacacaaaauucgcacaaacucgaguuuuguuguccaaggcuauggcugguggugauguguuauuggaugaguaucucuaugauguggucaauggacaagauuuuagagcuacugucgcuuuuuugcgcacccauguuauaacaggcaaaauaaaggugacagcuaccaccaacauuucugacaacucggguuguuguuugauguuggccauaaauaguggugugagggguaaguauaguacugauguuuauacuaucugcucucaagacuccaugacguggaacccagggugcaaaaagaacuucucguucacauuuaauccaaacccuuguggggauucuuggucugcugagaugauaagucgaagcagaguuaggaugacaguuauuuguguuucgggauggaccuuaucuccuaccacagaugugauugccaagcuagacuggucaauugucaaugagaaaugugagcccaccauuuaccacuuggcugauugucagaauugguuaccccuuaaucguuggaugggaaaauugacuuuuccccagggugugacaagugagguucgaaggaugccucuuucuauaggaggcggugcuggugcgacucaagcuuucuuggccaauaugcccaauucauggauaucaauguggagauauuuuagaggugaacuucacuuugaaguuacuaaaaugagcucuccauauauuaaagccacuguuacauuucucauagcuuuugguaaucuuagugaugccuuugguuuuuaugagaguuuuccucauagaauuguucaauuugcugagguugaggaaaaauguacuuugguuuucucccaacaagaguuugucacugcuuggucaacacaaguaaaccccagaaccacacuugaagcagaugguugucccuaccuauaugcaauuauucaugauaguacaacagguacaaucuccggagauuuuaaucuuggggucaagcuuguuggcauuaaggauuuuugugguauagguucuaauccggguauugaugguucccgcuugcuuggagcuauagcacaaggaccuguuugugcugaagccucagauguguauagcccauguaugauagcuagcacuccuccugcuccauuuucagacguuacagcaguaacuuuugacuuaaucaacggcaaaauaacuccuguuggugaugacaauuggaauacgcacauuuauaauccuccaauuaugaaugucuugcguacugcugcuuggaaaucuggaacuauucauguucaacuuaauguuaggggugcuggugucaaaagagcagauugggauggucaagucuuuguuuaccugcgccaguccaugaacccugaaaguuaugaugcgcggacauuugugaucucacaaccugguucugccauguugaacuucucuuuugauaucauagggccgaauagcggauuugaauuugccgaaagcccaugggccaaucagaccaccugguaucuugaauguguugcuaccaaucccagacaaauacagcaauuugaggucaacaugcgcuucgauccuaauuucaggguugccggcaauauccugaugcccccauuuccacugucaacggaaacuccaccguuauuaaaguuuagguuucgggauauugaacgcuccaagcguaguguuaugguuggacacacugcuacugcugcuuaacucugguuucauuaaauuuucuuuaguuugaauuuacuguuauucggugugcauuucuauguuuggugagcgguuuucugugcucagaguguguuuauuuuauguaauuuaauuucuuugugagcuccuguuuagcaggucgucccuucagcaaggacacaaaaagauuuuaauuuuauu

**>GFP (encoded by plasmid pEAQ-GFP)** Blunt-end restriction site Nru1 site underlined

uauuaaaaucuuaauagguuuugauaaaagcgaacguggggaaacccgaaccaaaccuucuucuaaacucucucucaucucucuuaaagcaaacuucucucuugucuuucuugcaugagcgaucuucaacguugucagaucgugcuucggcaccaguacaauguuuucuuucacugaagcgaaaucaaagaucucuuuguggacacguagugcggcgccauuaaauaacguguacuuguccuauucuugucgguguggucuugggaaaagaaagcuugcuggaggcugcuguucagccccauacauuacuuguuacgauucugcugacuuucggcgggugcaauaucucuacuucugcuugacgagguauuguugccuguacuucuuucuucuucuucuugcugauugguucuauaagaaaucuaguauuuucuuugaaacagaguuuucccgugguuuucgaacuuggagaaagauuguuaagcuucuguauauucugcccaaauucgcgaugacuagcaaaggagaagaacuuuucacuggaguugucccaauucuuguugaauuagauggugauguuaaugggcacaaauuuucugucaguggagagggugaaggugaugcaacauacggaaaacuuacccuuaaauuuauuugcacuacuggaaaacuaccuguuccauggccaacacuugucacuacuuucucuuaugguguucaaugcuuuucaagauacccagaucauaugaaacggcaugacuuuuucaagagugccaugcccgaagguuauguacaggaaagaacuauauuuuucaaggaugacgggaacuacaagacacgugcugaagucaaguuugaaggugauacccuuguuaauagaaucgaguuaaaagguauugauuuuaaagaagauggaaacauucuuggacacaaauuggaauacaacuauaacucacacaauguauacaucauggcagacaaacaaaagaauggaaucaaaguuaacuucaaaauuagacacaacauugaagauggaagcguucaacuagcagaccauuaucaacaaaauacuccaauuggcgauggcccuguccuuuuaccagacaaccauuaccuguccacacaaucugcccuuucgaaagaucccaacgaaaagagagaccacaugguccuucuugaguuuguaacagcugcugggauuacacauggcauggaugaacuauacaaauaa**cucgag**gccuuuaacucugguuucauuaaauuuucuuuaguuugaauuuacuguuauucggugugcauuucuauguuuggugagcgguuuucugugcucagaguguguuuauuuuauguaauuuaauuucuuugugagcuccuguuuagcaggucgucccuucagcaaggacacaaaaagauuuuaauuuuauuaaaaaaaaaaaaaaaaaa

**>SARS-CoV-2-S (encoded by plasmid pEAQ-S)**

uauuaaaaucuuaauagguuuugauaaaagcgaacguggggaaacccgaaccaaaccuucuucuaaacucucucucaucucucuuaaagcaaacuucucucuugucuuucuugcaugagcgaucuucaacguugucagaucgugcuucggcaccaguacaauguuuucuuucacugaagcgaaaucaaagaucucuuuguggacacguagugcggcgccauuaaauaacguguacuuguccuauucuugucgguguggucuugggaaaagaaagcuugcuggaggcugcuguucagccccauacauuacuuguuacgauucugcugacuuucggcgggugcaauaucucuacuucugcuugacgagguauuguugccuguacuucuuucuucuucuucuugcugauugguucuauaagaaaucuaguauuuucuuugaaacagaguuuucccgugguuuucgaacuuggagaaagauuguuaagcuucuguauauucugcccaaauucg**accggu**AUGUUCGUGUUCCUUGUGCUUCUUCCGCUGGUGUCAUCUCAGUGCGUGAACCUUACUACUAGGACCCAACUUCCUCCGGCCUACACCAAUUCUUUCACUAGGGGAGUGUACUACCCGGACAAGGUGUUCAGGUCAUCUGUGCUUCAUUCUACCCAGGACCUGUUCCUGCCGUUCUUCUCUAAUGUGACCUGGUUCCACGCUAUCCAUGUGUCUGGUACUAAUGGCACCAAGCGGUUCGAUAACCCUGUGCUGCCUUUCAACGAUGGCGUGUACUUUGCUAGCACCGAGAAGUCCAACAUCAUCCGUGGUUGGAUCUUCGGCACCACCUUGGAUUCUAAGACCCAGUCUCUUCUGAUCGUGAACAACGCUACCAACGUGGUGAUUAAGGUGUGCGAGUUCCAGUUCUGCAACGACCCUUUCCUUGGCGUCUACUACCACAAGAACAACAAGAGCUGGAUGGAAAGCGAGUUCCGGGUGUACUCUUCUGCUAACAAUUGCACCUUCGAGUACGUGAGCCAGCCUUUCUUGAUGGAUCUUGAAGGUAAGCAGGGCAACUUCAAGAACCUGCGGGAAUUCGUGUUCAAGAACAUCGACGGCUACUUUAAGAUCUACAGCAAGCACACCCCGAUCAACCUUGUGAGAGAUCUUCCUCAGGGCUUCUCUGCUCUUGAGCCUCUUGUGGAUCUGCCUAUCGGUAUCAACAUUACCCGGUUCCAGACCUUGCUUGCUCUGCACAGGUCUUAUCUUACCCCUGGCGAUUCUUCUUCUGGUUGGACUGCUGGUGCAGCUGCUUACUACGUUGGUUAUCUUCAGCCUAGGACCUUCCUGCUGAAGUACAACGAGAACGGCACCAUUACCGAUGCUGUGGAUUGUGCUUUGGACCCGCUUUCUGAGACUAAGUGCACCCUGAAGUCUUUCACCGUUGAGAAGGGAAUCUACCAGACCAGCAACUUUAGGGUGCAGCCUACCGAGUCUAUUGUGCGGUUCCCUAACAUCACCAACUUGUGCCCUUUCGGCGAGGUGUUCAAUGCUACUAGGUUCGCUUCUGUGUACGCCUGGAACCGUAAGCGGAUUUCUAACUGCGUGGCCGAUUACAGCGUGCUGUACAACUCUGCUAGCUUCAGCACCUUCAAGUGCUACGGUGUGUCUCCUACCAAGCUGAACGAUCUCUGCUUCACCAACGUGUACGCUGACUCUUUCGUGAUCAGGGGUGAUGAGGUUAGGCAGAUUGCUCCUGGUCAGACCGGAAAGAUCGCUGACUACAACUACAAGCUGCCGGAUGAUUUCACCGGAUGCGUGAUCGCUUGGAACAGCAACAACCUGGAUUCAAAGGUUGGCGGCAAUUACAACUACCUCUACCGGCUGUUCCGGAAGUCUAACCUUAAGCCUUUCGAGCGGGAUAUCUCCACCGAGAUCUAUCAGGCUGGUUCUACUCCUUGCAACGGUGUUGAGGGUUUCAACUGCUACUUCCCGCUUCAGUCAUACGGUUUCCAGCCUACUAAUGGUGUGGGCUACCAGCCUUACAGAGUGGUGGUUUUGUCUUUCGAGCUUCUGCAUGCUCCUGCUACUGUUUGCGGUCCUAAGAAGUCUACCAACCUGGUCAAGAACAAGUGCGUCAACUUCAAUUUCAACGGCCUGACCGGAACUGGUGUGCUGACUGAAUCUAACAAGAAGUUCCUGCCUUUCCAGCAGUUCGGCAGGGAUAUUGCUGAUACCACUGAUGCUGUUCGGGACCCUCAGACCUUGGAGAUUCUUGAUAUUACCCCGUGCAGCUUCGGUGGCGUGUCAGUUAUUACUCCUGGCACCAACACCUCUAACCAGGUGGCAGUUCUUUACCAGGACGUGAACUGUACUGAGGUGCCAGUGGCUAUUCACGCUGAUCAGUUGACUCCUACUUGGCGGGUUUACAGCACCGGAUCUAAUGUGUUCCAGACUAGAGCUGGUUGCCUGAUCGGUGCUGAGCAUGUGAACAAUAGCUACGAGUGCGAUAUCCCUAUCGGCGCUGGUAUUUGCGCUUCUUACCAGACUCAGACCAACUCUCCUAGAAGGGCUAGAUCUGUGGCCAGCCAGUCUAUUAUCGCUUACACCAUGUCUCUGGGCGCCGAGAAUUCUGUGGCUUACUCCAACAACUCUAUCGCUAUCCCGACCAACUUCACCAUCUCUGUGACUACCGAGAUCCUGCCUGUGUCUAUGACUAAGACCUCAGUGGACUGCACCAUGUACAUCUGCGGUGAUUCUACCGAGUGCUCUAACCUGCUUCUGCAGUACGGUUCUUUCUGCACCCAGCUUAACAGGGCUCUUACCGGAAUUGCUGUUGAGCAGGACAAGAACACCCAAGAGGUUUUCGCUCAGGUCAAGCAGAUCUAUAAGACCCCUCCGAUCAAGGAUUUCGGCGGCUUUAACUUCAGCCAGAUUCUGCCUGAUCCGAGCAAGCCGUCUAAGCGGUCUUUCAUUGAGGACCUGCUGUUCAACAAGGUGACCCUUGCUGAUGCUGGCUUCAUUAAGCAGUACGGCGAUUGCCUUGGUGAUAUCGCUGCUAGGGAUCUGAUCUGCGCUCAGAAGUUCAACGGUCUUACUGUGCUUCCUCCUCUGCUGACCGAUGAGAUGAUUGCCCAGUACACCUCUGCUCUGCUUGCUGGAACUAUUACCUCUGGAUGGACUUUCGGAGCUGGUGCUGCACUUCAGAUUCCUUUCGCUAUGCAGAUGGCCUACAGGUUCAAUGGUAUUGGCGUGACCCAGAACGUCCUGUACGAGAACCAGAAGCUUAUCGCCAACCAGUUCAACAGCGCUAUCGGCAAGAUCCAGGACUCCCUUUCUUCUACCGCUUCUGCUUUGGGUAAGCUGCAGGAUGUGGUGAAUCAGAACGCUCAGGCUCUUAACACCCUCGUGAAGCAGCUGUCAUCUAACUUCGGCGCUAUCAGCUCCGUGCUCAACGAUAUUCUUAGCCGGCUGGAUAAGGUUGAGGCUGAGGUUCAGAUUGACAGGCUUAUUACCGGCAGGCUUCAGAGCCUUCAGACUUACGUUACCCAGCAGCUUAUUCGGGCUGCUGAGAUUAGGGCUUCAGCUAAUCUUGCUGCCACCAAGAUGAGCGAGUGUGUGCUUGGUCAAAGCAAGAGGGUUGACUUCUGCGGUAAGGGUUACCACCUGAUGUCUUUCCCUCAAUCUGCUCCUCAUGGCGUGGUGUUCCUUCAUGUUACUUACGUGCCAGCUCAAGAGAAGAAUUUCACCACCGCUCCAGCUAUCUGCCACGAUGGUAAGGCUCAUUUUCCUCGUGAGGGCGUGUUCGUGUCUAAUGGUACUCAUUGGUUCGUCACCCAGCGGAACUUUUACGAGCCUCAGAUUAUCACCACCGACAACACCUUCGUGAGCGGCAAUUGUGAUGUUGUGAUCGGCAUUGUGAACAACACCGUGUACGAUCCACUUCAGCCUGAGCUGGACAGCUUCAAAGAGGAACUGGACAAGUACUUCAAAAACCACACCUCUCCAGAUGUGGACCUGGGUGAUAUCUCUGGGAUCAAUGCUAGCGUGGUGAACAUCCAGAAAGAAAUCGACCGGCUUAACGAGGUGGCAAAGAACCUUAACGAGAGCCUGAUUGACCUGCAAGAGCUUGGUAAGUACGAGCAGUACAUCAAGUGGCCGUGGUACAUUUGGCUGGGUUUCAUUGCUGGCCUGAUCGCUAUUGUGAUGGUGACCAUUAUGCUGUGCUGCAUGACCUCUUGCUGCUCUUGUCUUAAGGGAUGCUGCUCAUGUGGGAGCUGCUGCAAGUUUGAUGAGGAUGAUUCUGAGCCUGUGCUGAAGGGUGUGAAGUUGCAUUACACCUAG**CUCGAG**gccuuuaacucugguuucauuaaauuuucuuuaguuugaauuuacuguuauucggugugcauuucuauguuuggugagcgguuuucugugcucagaguguguuuauuuuauguaauuuaauuucuuugugagcuccuguuuagcaggucgucccuucagcaaggacacaaaaagauuuuaauuuuauuaaaaaaaaaaaaaaaaaa

**>DENV (encoded by plasmid pEAQ-DENV1-SP)**

uauuaaaaucuuaauagguuuugauaaaagcgaacguggggaaacccgaaccaaaccuucuucuaaacucucucucaucucucuuaaagcaaacuucucucuugucuuucuugcaugagcgaucuucaacguugucagaucgugcuucggcaccaguacaauguuuucuuucacugaagcgaaaucaaagaucucuuuguggacacguagugcggcgccauuaaauaacguguacuuguccuauucuugucgguguggucuugggaaaagaaagcuugcuggaggcugcuguucagccccauacauuacuuguuacgauucugcugacuuucggcgggugcaauaucucuacuucugcuugacgagguauuguugccuguacuucuuucuucuucuucuugcugauugguucuauaagaaaucuaguauuuucuuugaaacagaguuuucccgugguuuucgaacuuggagaaagauuguuaagcuucuguauauucugcccaaauucg**accggu**caaugaacaaccagaggaagaaaaccggcaggccgucuuucaacaugcugaagagagcuaggaaccggguuucaaccguuucucagcuugccaagagguucucuaagggucugcuuucuggucaggguccuaugaagcuugugauggccuuuaucgccuuccugcgguuccuugcuauuccuccuacugcugguauucuugcuagguggggcagcuucaagaagaacggcgcuauuaaggugcugcgggguuucaagaaagaaaucucuaacaugcuguccaucaugaaccggcggaagagaucugugacuaugcuucuuaugcugcugccuaccgcucuugcuuuccaucuuacuacuagagguggcgagccucacaugaucgugucuaagcaagagaggggcaagagccuucuguucaagacuucugcuggugugaacaugugcacccugaucgcuauggaucuuggugagcuuugcgaggacaccaugacuuacaagugcccuaggauuacugaggcugagccugaugauguugacugcuggugcaaugcuacugauaccuggguuaccuacggaaccuguucucagacuggugagcacagaagggacaagcguucuguugcucuugcuccucauguuggucugggucuugaaacuagagccgagacuuggaugucaucugaaggcgcuuggaagcagauccagaagguugaaacuugggcucuuaggcacccugguuuuacugugaccgcucuuuuccuugcucacgcuauuggcaccucuaucacccagaaggggaucaucuucauccugcuuaugcuggugaccccgagcauggcuaugagauguguugguaucggcaaccgggauuucguugagggacuuucuggugcuacuuggguugacguugugcuugagcaugguucuugcgugaccaccauggcuaaggauaagccgaccuuggauaucgagcugcucaagacugaggugaccaauccugcugugcuuaggaaguugugcaucgaggccaagaucucaaacaccaccacugauucuaggugcccuacucagggugaagcuacccuuguugaagaacaggacgcuaacuucgugugccguaggacuuucguugauagaggaugggguaacgguugcggucuuuucgguaaggguucucugauuaccugcgccaaguucaagugcgucacaaagcuugaggguaagaucgugcaguacgagaaccugaaguacagcgugaucgugacugugcacacuggugaucagcaucaggugggaaaugagacuacugagcacgguacuaccgcuacuauuacuccucaggcuccuaccucugagauccagcuuacugauuacggugcucugacccuggauugcucuccuagaacuggucuggacuucaacgagauggugcugcugaccaugaaggaaaagucuuggcugguucacaagcagugguuccuugaucuuccuuugccuuggaccucuggugcuucuacuccucaagagacauggaacaggcaggaucuucuggugaccuucaagaccgcucacgccaagaagcaagagguugucguucuugguagucaagagggugcuaugcacaccgcucuuacaggugcuacugagauucagacuucuggcaccaccaccauuuucgcuggucaucuuaagugccggcugaagauggauaagcuuacccugaagggcaugagcuacgugaugugcacugguuccuucaaguuggagaaagagguggcagagacacagcaugguacugugcuuguucaggugaaguacgaggguacugaugcuccuugcaagaucccauucucuacccaggaugagaagggugugacucagaacgguaggcugauuacugcuaacccuaucgugaccgacaaagagaagccggucaauaucgaagcugagccuccauucggugagagcuacauugugguuggugcuggugagaaggcacugaagcuuagcugguucaagaagggcagcuccaucgguaagaugcuugaggcuacagcuagaggcgcuagaaggauggcuauucuuggugauaccgcuugggacuucgguucuauuggugguguguucaccucugugggaaagcuugugcaccagauuuucggaacugcuuacggcguguuguucagcgguguuagcuggacuaugaagaucggcauugguauccugcugaccuggcuuggucuuaacucuaggucuaccagccugagcaugaccugcauugcuguugguauggugacccuuuaccuggguguuauggugcaggcuuag**cucgag**gccuuuaacucugguuucauuaaauuuucuuuaguuugaauuuacuguuauucggugugcauuucuauguuuggugagcgguuuucugugcucagaguguguuuauuuuauguaauuuaauuucuuugugagcuccuguuuagcaggucgucccuucagcaaggacacaaaaagauuuuaauuuuauuaaaaaaaaaaaaaaaaaa

**>ZikaV (encoded by plasmid pEAQ-ZIKAV-SP)**

uauuaaaaucuuaauagguuuugauaaaagcgaacguggggaaacccgaaccaaaccuucuucuaaacucucucucaucucucuuaaagcaaacuucucucuugucuuucuugcaugagcgaucuucaacguugucagaucgugcuucggcaccaguacaauguuuucuuucacugaagcgaaaucaaagaucucuuuguggacacguagugcggcgccauuaaauaacguguacuuguccuauucuugucgguguggucuugggaaaagaaagcuugcuggaggcugcuguucagccccauacauuacuuguuacgauucugcugacuuucggcgggugcaauaucucuacuucugcuugacgagguauuguugccuguacuucuuucuucuucuucuugcugauugguucuauaagaaaucuaguauuuucuuugaaacagaguuuucccgugguuuucgaacuuggagaaagauuguuaagcuucuguauauucugcccaaauuc**accggu**augaagaacccuaagaaaaagagcggugguuucaggaucgugaacaugcuuaagagggguguggcuaggguuucaccuuucgguggucuuaagagacuuccugcuggucugcuucuuggucacgguccuauuaggauggugcuugcuauucuggcuuuccugagguucaccgcuaucaagccuucucugggucuuaucaacaggugggguagcguggguaagaaagaagcuauggaaaucaucaagaaguucaagaaagaucuggcugcuaugcugaggaucaucaacgcuaggaaagaaaagaagagaaggggagcugauaccuccguugguauuguggguuugcuucugacuaccgcuauggcugcugagguuacaagaagggguagcgcuuacuacauguaccuggauaggaacgaugcuggugaggcuaucucuuucccuaccacccuugguaugaacaagugcuacauccagauuauggaucugggucacaugugugaugcuaccaugucuuacgagugcccuaugcuugaugaggguguugagccugaugauguggauugcuggugcaauaccacuucuaccugggugguguacgguacuugccaccauaagaagggugaggcaagaaggucuagaagggcuguuacucugccuagccacucuacuaggaagcuucagacuaggucacagaccuggcuugagucuagagaguacaccaagcaccuuaucaggguugagaacuggaucuuuaggaacccugguuucgcucuugcugcugcugcaauugcuuggcuucuuggaucuagcaccagccagaaggugaucuaccugguuaugauccugcugaucgcuccugcuuacagcaucagaugcaucggugugagcaacagggauuucguugagggaaugucuggugguacuuggguggacauugugcuugagcauggugguugcguuaccgugauggcucaagauaagccuaccguggauauugagcuugugaccaccaccguguccaauauggcugaggugaggucuuauugcuacgaggcuucuaucagcgauauggcuagcgauucuaggugcccuacucagggugaagcuuaccuugauaagcaguccgauacccaguacgugugcaagaggacucuuguugauagggguugggguaacgguugcggucuuuuugguaaggguucucuugugaccugcgcuaaguucgcuugcagcaagaagaugaccggaaagucuauccagccugagaaccuugaguacaggaucaugcuuagcgugcacgguucucagcacucugguaugauugugaacgauaccggacacgagacugaugagaauagggcuaagguugagaucaccccuaacucuccaagagcugaggcuacacuuggugguuucgguagccuuggucuugauugcgaaccuaggaccggacuggauuucagcgaucuuuacuaccugaccaugaacaacaagcacugguuggugcacaaagagugguuccacgauauuccuuugccuuggcaugcuggugcugauacugguacuccucacuggaacaacaaagaggcucugguugaguucaaggaugcucacgcuaagaggcagacuguuguggugcuuggaucucaagagggugcugugcauacugcauuagcuggugcucuugaggcugagauggauggugcaaaggguaggcuuucaucuggucaccuuaagugcaggcuuaagauggauaagcuuaggcugaagggugugagcuacucucugugcacugcugcuuuuaccuucaccaagaucccugcugagacucuucaugguacugugaccguugagguucaguacgcugguacugaugguccauguaagguuccagcucagauggcuguggauaugcagacucuuacuccuguggguaggcugauuacugcuaacccugugauuaccgagagcaccgagaacagcaagaugaugcuugagcuggauccuccauucggugauagcuacauugugaucggugugggugagaagaagaucacucaucacuggcacagguccgguucuaccauugguaaggcuuucgaggcuacugugagaggugcuaagaggauggcuguucuuggugauaccgcuugggauuucgguucuguuggaggugcucuuaacagccuggguaaggguauucaccagaucuucggugcugcuuucaagagccuuuucgguggaaugagcugguucucccagauucuuauuggaacccugcugauguggcugggucugaauaccaagaacgguagcaucucucugaugugccuugcucuugguggugugcugauuuuccuuagcaccgcugugucugcugauuag**cucgag**gccuuuaacucugguuucauuaaauuuucuuuaguuugaauuuacuguuauucggugugcauuucuauguuuggugagcgguuuucugugcucagaguguguuuauuuuauguaauuuaauuucuuugugagcuccuguuuagcaggucgucccuucagcaaggacacaaaaagauuuuaauuuuauuaaaaaaaaaaaaaaaaaa

**>CHIKV (encoded by plasmid** **pEAQ-CHIKV-SP)**

uauuaaaaucuuaauagguuuugauaaaagcgaacguggggaaacccgaaccaaaccuucuucuaaacucucucucaucucucuuaaagcaaacuucucucuugucuuucuugcaugagcgaucuucaacguugucagaucgugcuucggcaccaguacaauguuuucuuucacugaagcgaaaucaaagaucucuuuguggacacguagugcggcgccauuaaauaacguguacuuguccuauucuugucgguguggucuugggaaaagaaagcuugcuggaggcugcuguucagccccauacauuacuuguuacgauucugcugacuuucggcgggugcaauaucucuacuucugcuugacgagguauuguugccuguacuucuuucuucuucuucuugcugauugguucuauaagaaaucuaguauuuucuuugaaacagaguuuucccgugguuuucgaacuuggagaaagauuguuaagcuucuguauauucugcccaaauucg**accggu**caauggaauucaucccgacucagaccuucuacaacaggcguuaucaaccuaggccuuggacuccuaggccaaccauucaaguuauuaggccuagaccuaggccacagagacaagcuggucaacuugcucagcuuaucagcgcugugaacaagcuuacuaugagggcuguuccucagcagaagccuaggcguaaucggaagaacaagaagcagaagcaaaagcagcaggccccucagaacaauaccaaccagaagaagcagccgccuaagaagaagccugcucaaaagaagaagaaacccggccguagagaacggaugugcaugaagauugagaacgacugcaucuucgaggugaagcacgaagguaaggugacagguuacgcuugccuugugggugacaagguuaugaagccagcucacgugaagggcaccaucgauaaugcugaucuugcuaagcuggccuucaagcgguccucuaaguaugaucuugagugcgcucagaucccggugcacaugaagucugaugcuucuaaguucacccacgagaagccugagggcuacuacaauuggcaucauggugcugugcaguacagcggugguagguucacuauuccuacuggugcugguaagccuggugauucugguaggccuaucuucgauaacaagggcagagugguggcuaucguucuugguggugcuaaugagggugcuagaaccgcucuuucuguggugacuuggaacaaggacaucgugaccaagauuaccccugagggugcagaggaauggucccuugcuauuccugugaugugccugcuugccaacacuacuuucccuugcucucaaccuccuugcacuccuugcugcuaugagaaagagccugaagagacucugcggaugcuugaggauaaugugaugaggccugguuacuaccagcugcuucaggcuagucuuacuugcucuccucauaggcagcggaggucuaccaaggacaauuucaacguguacaaggccaccaggccuuacuuggcucauuguccugauuguggugagggacacucuugccauucuccaguugcucuugagcggauuaggaacgaggcuaccgauggcacccuuaagauucaggugagccuucagaucggcaucaagaccgaugauucucacgauuggaccaagcugcgguacauggauaaucacaugccugcugaugcugagagagcuggucuuuucguuaggacuucugcuccgugcacuauuaccggaacuaugggccacuucauucuggcuagauguccuaagggugagacucugacugugggcuucacugacucuaggaagaucucucacucuugcacucacccguuccaucaugauccuccagugaucgguagagagaaguuccauucuaggccucagcacgguaaagaguugccuugcucuacuuacgugcagucuacugcugcuaccaccgaagagauugagguucacaugccaccugauacaccagacaggacccuuaugucucagcagucugguaacgugaagaucaccgugaauggucagaccgugagguacaaguguaacugcggugguucuaacgagggacuuaccacuaccgacaaggugaucaacaacugcaagguggaccagugccaugcugcugugacuaaucauaagaaguggcaguacaacagcccgcuggugccuagaaaugcugagcuuggugauaggaagggcaagauucacaucccuuuuccgcuugcuaacgugaccugcagaguuccuaaggcuaggaauccuacugugaccuacggcaagaaccaggugaucaugcuucuguacccugaucacccuaccuugcugagcuauaggaacaugggcgaagaaccgaacuaccaagaggaaugggugaugcacaagaaagaaguggugcugacuguuccuaccgagggucuugaaguuaccugggguaacaacgagccguacaaguauuggccucagcugucuacuaacggaacugcucauggucacccucacgagaucauccuguacuacuacgagcuguacccuaccaugacugugguuguugugucuguggcuaccuucauccugcugucuaugguuggaauggcugcugguaugugcaugugcgcuagaaggcguugcauuaccccuuacgaacuuacuccuggugcuaccgugccuuuccuucugucucugauuugcugcaucaggaccgcuaaggcugcuaccuaucaagaggcugcuaucuaccuuuggaacgagcagcagccuuuguucuggcugcaagcucuuauuccucuggcugcucuuaucguccugugcaacugccuuagacuucugccuuguugcugcaagacccuggcuuucuuggcugugaugucugugggugcucauaccguuucugcuuacgagcaugugaccgugauuccuaauacugugggcgugccauacaagacccuugugaauaggccuggcuacagcccuauggugcuugagauggaacuuuugagcgugacccuugagccuacacugagccuugauuacauuaccugcgaguacaagacugugaucccgucaccauacguuaaguguugcgguacugcugaguguaaggacaagaaccugccugacuacucuugcaagguguucacugguguguacccuuucaugugggguggugccuauugcuucugugacgcugagaauacccagcugucugaggcucauguugagaagagugagucaugcaagaccgaguucgcuucagcuuacagggcucauacugcuuccgcuagcgcuaaguugagagugcuuuaccagggcaacaacauuaccgugaccgcuuacgcuaauggcgaucaugcuguuaccgugaaggacgccaaguucauugugggcccuaugucuagugcuuggaccccuuuugacaacaagaucguggucuacaagggcgacgucuacaacauggauuacccuccauuuggugcuggaaggccaggacaguuuggugauauucagucuaggaccccugagucaaaggacguguacgcuaauacccaguuggugcuucaaaggccugcuguggguacuguucaugugccuuauucucaggcuccgagcgguuucaaguacuggcuuaaagaaaggggcgcuucucuucagcauaccgcuccauucgguugccagauugcuacuaauccugugagggcagugaauugcgcugugggcaauaugccuaucagcaucgauauucccgaggccgcuuucaccagaguuguggaugcuccuucucugaccgauaugucuugcgaaguuccugcuugcacccacuccucugauuuuggugguguggccaucaucaaguacgccgcuucuaagaaaggcaagugcgcugugcauagcaugacuaacgcugugaccauucgugaggcugagauugaaguugagggcaacucucagcugcagaucucuuucucuaccgcucuugcuucagcugaguucagaguucaggugugcucuacucagguucacugugcugcugaaugccauccuccuaaggaucacaucgugaacuacccugcuucucacacuacccuuggugugcaggauaucucugcuaccgcuaugucaugggugcagaagauuacuggcggaguuggucuuguuguggcuguugcugcucugauucuuaucguggugcucugcgugagcuucucuaggcauuag**cucgag**gccuuuaacucugguuucauuaaauuuucuuuaguuugaauuuacuguuauucggugugcauuucuauguuuggugagcgguuuucugugcucagaguguguuuauuuuauguaauuuaauuucuuugugagcuccuguuuagcaggucgucccuucagcaaggacacaaaaagauuuuaauuuuauuaaaaaaaaaaaaaaaaaa

**>EBOV-GP-NA (encoded by plasmid pMOD-EAQ-EBOV-GP-NA)**

uauuaaaaucuuaauagguuuugauaaaagcgaacguggggaaacccgaaccaaaccuucuucuaaacucucucucaucucucuuaaagcaaacuucucucuugucuuucuugcaugagcgaucuucaacguugucagaucgugcuucggcaccaguacaauguuuucuuucacugaagcgaaaucaaagaucucuuuguggacacguagugcggcgccauuaaauaacguguacuuguccuauucuugucgguguggucuugggaaaagaaagcuugcuggaggcugcuguucagccccauacauuacuuguuacgauucugcugacuuucggcgggugcaauaucucuacuucugcuugacgagguauuguugccuguacuucuuucuucuucuucuugcugauugguucuauaagaaaucuaguauuuucuuugaaacagaguuuucccgugguuuucgaacuuggagaaagauuguuaagcuucuguauauucugcccaaauuc**aCCGGU**GAGAAUAAACUAGUAUUCUUCUGGUCCCCACAGACUCAGAGAGAACCCGCCACCaugggcguuacaggaauauugcaguuaccucgugaucgauucaagaggacaucauucuuucuuuggguaauuauccuuuuccaaagaacauuuuccaucccacuuggagucauccacaauagcacauuacagguuagugaugucgacaaacuaguuugucgugacaaacugucauccacaaaucaauugagaucaguuggacugaaucucgaagggaauggaguggcaacugacgugccaucugcaacuaaaagauggggcuucagguccggugucccaccaaagguggucaauuaugaagcuggugaaugggcugaaaacugcuacaaucuugaaaucaaaaaaccugacgggagugagugucuaccagcagcgccagacgggauucggggcuucccccggugccgguaugugcacaaaguaucaggaacgggaccgugugccggagacuuugccuuccauaaagagggugcuuucuuccuguaugaucgacuugcuuccacaguuaucuaccgaggaacgacuuucgcugaaggugucguugcauuucugauacugccccaagcuaagaaggacuucuucagcucacaccccuugagagagccggucaaugcaacggaggacccgucuaguggcuacuauucuaccacaauuagauaucaggcuacGggAuuuggaaccaaugagacagaguacuuguucgagguugacaauuugaccuacguccaacuugaaucaagauucacaccacaguuucugcuccagcugaaugagacaauauauacaagugggaaaaggagcaauaccacgggaaaacuaauuuggaaggucaaccccgaaauugauacaacaaucggggagugggccuucugggaaacuaaaaaaaaccucacuagaaaaauucgcagugaagaguugucuuucacaguuguaucaaacggagccaaaaacaucaguggucagaguccggcgcgaacuucuuccgacccagggaccaacacaacaacugaagaccacaaaaucauggcuucagaaaauuccucugcaaugguucaagugcacagucaaggaagggaagcugcagugucgcaucuaacaacccuugccacaaucuccacgaguccccaaucccucacaaccaaaccagguccggacaacagcacccauaauacacccguguauaaacuugacaucucugaggcaacucaaguugaacaacaucaccgcagaacagacaacgacagcacagccuccgacacucccucugccacgaccgcagccggacccccaaaagcagagaacaccaacacgagcaagagcacugacuuccuggaccccgccaccacaacaaguccccaaaaccacagcgagaccgcuggcaacaacaacacucaucaccaagauaccggagaagagagugccagcagcgggaagcuaggcuuaauuaccaauacuauugcuggagucgcaggacugaucacaggcgggagaagaacucgaagagaagcaauugucaaugcucaacccaaaugcaacccuaauuuacauuacuggacuacucaggaugaaggugcugcaaucggacuggccuggauaccauauuucgggccagcagccgagggaauuuacauagaggggcuaaugcacaaucaagaugguuuaaucuguggguugagacagcuggccaacgagacgacucaagcucuucaacuguuccugagagccacaacugagcuacgcaccuuuucaauccucaaccguaaggcaauugauuucuugcugcagcgauggggcggcacaugccacauucugggaccggacugcuguaucgaaccacaugauuggaccaagaacauaacagacaaaauugaucagauuauucaugauuuuguugauaaaacccuuccggaccagggggacaaugacaauugguggacaggauggagacaauggauaccggcagguauuggaguuacaggcguuauaauugcaguuaucgcuuuauucuguauaugcaaauuugucuuuuag**CUCGAG**gccuuuaacucugguuucauuaaauuuucuuuaguuugaauuuacuguuauucggugugcauuucuauguuuggugagcgguuuucugugcucagaguguguuuauuuuauguaauuuaauuucuuugugagcuccuguuuagcaggucgucccuucagcaaggacacaaaaagauuuuaauuuuauuaaaaaaaaaaaaaaaaaa

**>EBOV-GP-CO (encoded by plasmid pMOD-EAQ-EBOV-GP-CO)**

uauuaaaaucuuaauagguuuugauaaaagcgaacguggggaaacccgaaccaaaccuucuucuaaacucucucucaucucucuuaaagcaaacuucucucuugucuuucuugcaugagcgaucuucaacguugucagaucgugcuucggcaccaguacaauguuuucuuucacugaagcgaaaucaaagaucucuuuguggacacguagugcggcgccauuaaauaacguguacuuguccuauucuugucgguguggucuugggaaaagaaagcuugcuggaggcugcuguucagccccauacauuacuuguuacgauucugcugacuuucggcgggugcaauaucucuacuucugcuugacgagguauuguugccuguacuucuuucuucuucuucuugcugauugguucuauaagaaaucuaguauuuucuuugaaacagaguuuucccgugguuuucgaacuuggagaaagauuguuaagcuucuguauauucugcccaaauuc**aCCGGU**GAGAAUAAACUAGUAUUCUUCUGGUCCCCACAGACUCAGAGAGAACCCGCCACCAUGGGUGUUACUGGUAUUCUUCAGCUGCCUCGGGAUCGUUUCAAGCGGACUUCUUUCUUCCUGUGGGUGAUCAUCUUGUUCCAGCGGACCUUCUCUAUCCCUCUUGGUGUGAUUCACAACAGCACCCUCCAGGUGUCAGAUGUGGAUAAGCUUGUGUGCAGGGACAAGCUCAGCUCUACCAACCAGUUGAGAUCUGUGGGUCUGAACCUGGAAGGUAAUGGUGUGGCUACUGAUGUGCCUUCUGCUACUAAGAGGUGGGGCUUCAGAUCUGGUGUUCCUCCUAAGGUGGUGAACUAUGAAGCUGGUGAAUGGGCCGAGAACUGCUACAACCUUGAGAUCAAGAAGCCUGACGGUUCUGAGUGUUUGCCUGCUGCUCCUGAUGGUAUUAGGGGUUUUCCUAGGUGCCGGUACGUUCACAAGGUUUCAGGUACUGGUCCUUGCGCUGGUGAUUUCGCUUUUCACAAAGAGGGCGCAUUCUUCCUCUACGACCGUCUUGCUUCUACCGUGAUCUACAGGGGUACUACUUUCGCUGAGGGUGUUGUGGCUUUCCUUAUUCUGCCUCAGGCCAAGAAGGACUUCUUCUCAUCUCACCCUCUUCGUGAGCCUGUGAAUGCUACUGAGGAUCCUAGCAGCGGUUACUACUCUACCACCAUCAGGUAUCAGGCUACCGGCUUCGGUACUAACGAGACUGAGUACCUUUUCGAGGUGGACAACCUUACCUACGUGCAGCUUGAGUCUAGGUUUACCCCUCAGUUCCUUCUGCAGCUGAACGAGACUAUCUACACCAGCGGUAAGCGGUCUAACACCACCGGAAAGCUUAUCUGGAAGGUGAACCCUGAGAUCGACACCACUAUUGGUGAGUGGGCUUUCUGGGAGACUAAGAAGAACCUGACUCGGAAGAUCCGGUCCGAGGAACUUUCUUUCACUGUGGUGUCUAACGGCGCCAAGAAUAUCUCUGGUCAGUCUCCUGCUCGGACCUCUUCUGAUCCUGGUACUAAUACCACUACCGAGGACCACAAGAUCAUGGCCUCUGAGAACUCUAGCGCUAUGGUUCAGGUUCACUCUCAGGGUAGAGAGGCUGCUGUUUCUCACCUUACUACCCUGGCUACCAUCUCUACCUCUCCACAGUCUCUUACCACUAAGCCUGGGCCUGAUAACAGCACUCAUAACACCCCUGUGUACAAGCUGGACAUCUCUGAGGCUACUCAGGUUGAGCAGCAUCAUCGGAGGACCGAUAAUGACUCUACCGCUUCUGAUACCCCAAGCGCUACUACUGCUGCUGGUCCUCCAAAGGCUGAGAACACUAACACCAGCAAGUCUACCGACUUCCUGGAUCCUGCUACUACCACUUCUCCUCAGAACCACUCUGAGACUGCUGGCAACAACAAUACCCACCACCAGGAUACUGGUGAAGAGUCUGCUAGCUCUGGUAAGCUUGGUCUGAUCACCAACACUAUCGCUGGUGUUGCUGGUCUUAUUACCGGCGGUAGAAGGACUAGAAGGGAAGCUAUCGUUAACGCUCAGCCUAAGUGCAACCCUAACCUUCACUACUGGACUACCCAGGAUGAGGGUGCAGCUAUUGGUCUUGCUUGGAUCCCUUACUUUGGUCCUGCUGCUGAGGGCAUCUACAUUGAGGGUCUUAUGCACAACCAGGACGGUCUUAUUUGCGGUCUUAGGCAGCUGGCUAAUGAGACUACUCAAGCACUGCAGCUGUUCCUUAGGGCUACUACAGAGCUGAGGACCUUCAGCAUCCUGAACAGAAAGGCCAUCGACUUUCUGCUGCAAAGGUGGGGUGGUACUUGCCACAUUCUUGGUCCUGAUUGCUGCAUCGAGCCUCACGAUUGGACUAAGAACAUCACCGACAAGAUCGACCAGAUCAUCCACGACUUCGUGGACAAGACUCUUCCUGACCAGGGCGAUAAUGAUAACUGGUGGACUGGUUGGAGGCAGUGGAUUCCUGCUGGUAUUGGUGUGACUGGCGUGAUAAUUGCUGUGAUCGCUCUGUUCUGCAUCUGCAAGUUCGUGUUCUAG**CUCGAG**gccuuuaacucugguuucauuaaauuuucuuuaguuugaauuuacuguuauucggugugcauuucuauguuuggugagcgguuuucugugcucagaguguguuuauuuuauguaauuuaauuucuuugugagcuccuguuuagcaggucgucccuucagcaaggacacaaaaagauuuuaauuuuauuaaaaaaaaaaaaaaaaaa

**>avHA (encoded by plasmid pEAQ-avHA)**

uauuaaaaucuuaauagguuuugauaaaagcgaacguggggaaacccgaaccaaaccuucuucuaaacucucucucaucucucuuaaagcaaacuucucucuugucuuucuugcauauggagcgaucuucaacguugucagaucgugcuucggcaccaguacaauguuuucuuucacugaagcgaaaucaaagaucucuuuguggacacguagugcggcgccauuaaauaacguguacuuguccuauucuugucgguguggucuugggaaaagaaagcuugcuggaggcugcuguucagccccauacauuacuuguuacgauucugcugacuuucggcgggugcaauaucucuacuucugcuugacgagguauuguugccuguacuucuuucuucuucuucuugcugauugguucuauaagaaaucuaguauuuucuuugaaacagaguuuucccgugguuuucgaacuuggagaaagauuguuaagcuucuguauauucugcccaaauuc**aCCGGU**AUGGAAAACAUAGUACUUCUUCUUGCAAUAGUUAGCCUUGUUAAAAGUGAUCAGAUUUGCAUUGGUUACCACGCAAACAAUUCGACAGAGCAGGUUGACACGAUAAUGGAAAAGAACGUUACUGUUACACAUGCCCAAGACAUACUGGAAAAAACACACAACGGGAAGCUCUGUGAUCUAAAUGGGGUGAAGCCUCUGAUUUUAAAGGAUUGUAGUGUAGCUGGAUGGCUCCUCGGAAACCCAAUGUGCGACGAAUUCAUCAGAGUGCCGGAAUGGUCCUACAUAGUGGAGCGGGCUAAUCCAGCUAAUGACCUCUGUUACCCAGGGAGCCUCAAUGACUAUGAAGAACUGAAACACCUGUUGAGCAGAAUAAAUCAUUUUGAGAAGAUUCUGAUCAUCCCCAAAAGUUCCUGGCCAAAUCAUGAAACAUCACUAGGGAUGAGCGCAGCUUGUCCUUAUCAGGGAGCGCCCUCCUUUUUCAGAAAUGUGGUGUGGCUUAUCAAAAAGAACGAUGCAUACCCAACAAUAAAGAUAAGCUACAAUAAUACCAAUCGGGAAGAUCUCUUGAUACUGUGGGGGAUUCAUCAUUCCAACAAUGCAGAAGAGCAGACAAAUCUCUAUAAAAACCCAACCACCUACAUUUCGGUUGGAACAUCAACUUUAAACCAGAGGUUGGUACCAAAAAUAGCUACUAGAUCCCAAGUAAACGGGCAACGUGGAAGAAUGGACUUCUUCUGGACAAUUUUAAAACCAGAUGAUGCAAUCCAUUUCGAGAGUAAUGGAAAUUUCAUUGCUCCAGAAUAUGCAUAUAAAAUUGUCAAGAAAGGGGACUCAACAAUUAUGAAAAGUGGAGUGGAAUAUGGCCAUUGCAACACCAAAUGUCAAACCCCAGUAGGAGCGAUAAAUUCUAGUAUGCCAUUCCACAACAUACAUCCUCUCACCAUUGGGGAAUGCCCCAAAUACGUGAAGUCAAACAAGUUGGUCCUUGCGACUGGGCUCAGAAAUAGUCCUCUAAGAGAAAAGAGAAGAAAAAGAGGCCUGUUUGGGGCGAUAGCAGGGUUUAUAGAGGGAGGAUGGCAGGGAAUGGUUGAUGGUUGGUAUGGGUACCAUCAUAGCAAUGAGCAGGGGAGUGGGUACGCUGCAGACAAAGAAUCCACCCAAAAGGCAAUAGAUGGAGUUACCAAUAAGGUCAACUCAAUCAUUGACAAAAUGAACACUCAAUUUGAAGCAGUUGGAAGGGAGUUUAAUAACUUAGAAAGGAGGAUAGAGAAUUUGAACAAAAAAAUGGAAGACGGAUUCCUAGAUGUCUGGACCUAUAAUGCUGAACUUCUAGUUCUCAUGGAAAACGAGAGGACUCUAGAUUUCCAUGAUUCAAAUGUCAAGAACCUUUACGACAAAGUCAGACUACAGCUUAGGGAUAAUGCAAAGGAGCUGGGUAACGGCUGUUUCGAAUUCUAUCACAAAUGCGAUAAUGAAUGUAUGGAAAGUGUGAGAAAUGGGACGUAUGACUACCCUCAGUAUUCAGAAGAAGCAAGAUUAAAAAGAGAAGAAAUAAGCGGAGUGAAAUUAGAAUCAAUAGGAACUUACCAGAUACUGUCAAUUUAUUCAACAGCGGCGAGUUCCCUAGCACUGGCAAUCAUGAUGGCUGGUCUAUCUUUAUGGAUGUGCUCCAAUGGGUCGUUACAGUGCAGAAUUUGCAUUUAGCG**CUCGAG**gccuuuaacucugguuucauuaaauuuucuuuaguuugaauuuacuguuauucggugugcauuucuauguuuggugagcgguuuucugugcucagaguguguuuauuuuauguaauuuaauuucuuugugagcuccuguuuagcaggucgucccuucagcaaggacacaaaaagauuuuaauuuuauuaaaaaaaaaaaaaaaaaa

**> CHIKV-SP-ZIKAV-10% (encoded by plasmid pEAQ-CHIKV-SP-ZIKAV-10%)**

uauuaaaaucuuaauagguuuugauaaaagcgaacguggggaaacccgaaccaaaccuucuucuaaacucucucucaucucucuuaaagcaaacuucucucuugucuuucuugcaugagcgaucuucaacguugucagaucgugcuucggcaccaguacaauguuuucuuucacugaagcgaaaucaaagaucucuuuguggacacguagugcggcgccauuaaauaacguguacuuguccuauucuugucgguguggucuugggaaaagaaagcuugcuggaggcugcuguucagccccauacauuacuuguuacgauucugcugacuuucggcgggugcaauaucucuacuucugcuugacgagguauuguugccuguacuucuuucuucuucuucuugcugauugguucuauaagaaaucuaguauuuucuuugaaacagaguuuucccgugguuuucgaacuuggagaaagauuguuaagcuucuguauauucugcccaaauucg**accggu**caauggaauucaucccgacucagaccuucuacaacaggcguuaucaaccuaggccuuggacuccuaggccaaccauucaaguuauuaggccuagaccuaggccacagagacaagcuggucaacuugcucagcuuaucagcgcugugaacaagcuuacuaugagggcuguuccucagcagaagccuaggcguaaucggaagaacaagaagcagaagcaaaagcagcaggccccucagaacaauaccaaccagaagaagcagccgccuaagaagaagccugcucaaaagaagaagaaacccggccguagagaacggaugugcaugaagauugagaacgacugcaucuucgaggugaagcacgaagguaaggugacagguuacgcuugccuugugggugacaagguuaugaagccagcucacgugaagggcaccaucgauaaugcugaucuugcuaagcuggccuucaagcgguccucuaaguaugaucuugagugcgcucagaucccggugcacaugaagucugaugcuucuaaguucacccacgagaagccugagggcuacuacaauuggcaucauggugcugugcaguacagcggugguagguucacuauuccuacuggugcugguaagccuggugauucugguaggccuaucuucgauaacaagggcagagugguggcuaucguucuugguggugcuaaugagggugcuagaaccgcucuuucuguggugacuuggaacaaggacaucgugaccaagauuaccccugagggugcagaggaauggucccuugcuauuccugugaugugccugcuugccaacacuacuuucccuugcucucaaccuccuugcacuccuugcugcuaugagaaagagccugaagagacucugcggaugcuugaggauaaugugaugaggccugguuacuaccagcugcuucaggcuagucuuacuugcucuccucauaggcagcggaggucuaccaaggacaauuucaacguguacaaggccaccaggccuuacuuggcucauuguccugauuguggugagggacacucuugccauucuccaguugcucuugagcggauuaggaacgaggcuaccgauggcacccuuaagauucaggugagccuucagaucggcaucaagaccgaugauucucacgauuggaccaagcugcgguacauggauaaucacaugccugcugaugcugagagagcuggucuuuucguuaggacuucugcuccgugcacuauuaccggaacuaugggccacuucauucuggcuagauguccuaagggugagacucugacugugggcuucacugacucuaggaagaucucucacucuugcacucacccguuccaucaugauccuccagugaucgguagagagaaguuccauucuaggccucagcacgguaaagaguugccuugcucuacuuacgugcagucuacugcugcuaccaccgaagagauugagguucacaugccaccugauacaccagacaggacccuuaugucucagcagucugguaacgugaagaucaccgugaauggucagaccgugagguacaaguguaacugcggugguucuaacgagggacuuaccacuaccgacaaggugaucaacaacugcaagguggaccagugccaugcugcugugacuaaucauaagaaguggcaguacaacagcccgcuggugccuagaaaugcugagcuuggugauaggaagggcaagauucacaucccuuuuccgcuugcuaacgugaccugcagaguuccuaaggcuaggaauccuacugugaccuacggcaagaaccaggugaucaugcuucuguacccugaucacccuaccuugcugagcuauaggaacaugggcgaagaaccgaacuaccaagaggaaugggugaugcacaagaaagaaguggugcugacuguuccuaccgagggucuugaaguuaccugggguaacaacgagccguacaaguauuggccucagcugucuacuaacggaacugcucauggucacccucacgagaucauccuguacuacuacgagcuguacccuaccaugacugugguuguugugucuguggcuaccuucauccugcugucuaugguuggaauggcugcugguaugugcaugugcgcuagaaggcguugcauuaccccuuacgaacuuacuccuggugcuaccgugccuuuccuucugucucugauuugcugcaucaggaccgcuaaggcugcuaccuaucaagaggcugcuaucuaccuuuggaacgagcagcagccuuuguucuggcugcaagcucuuauuccucuggcugcucuuaucguccugugcaacugccuuagacuucugccuuguugcugcaagacccuggcuuucuuggcugugaugucugugggugcucauaccguuucugcuuacgagcaugugaccgugauuccuaauacugugggcgugccauacaagacccuugugaauaggccuggcuacagcccuauggugcuugagauggaacuuuugagcgugacccuugagccuacacugagccuugauuacauuaccugcgaguacaagacugugaucccgucaccauacguuaaguguugcgguacugcugaguguaaggacaagaaccugccugacuacucuugcaagguguucacugguguguacccuuucaugugggguggugccuauugcuucugugacgcugagaauacccagcugucugaggcucauguugagaagagugagucaugcaagaccgaguucgcuucagcuuacagggcucauacugcuuccgcuagcgcuaaguugagagugcuuuaccagggcaacaacauuaccgugaccgcuuacgcuaauggcgaucaugcuguuaccgugaaggacgccaaguucauugugggcccuaugucuagugcuuggaccccuuuugacaacaagaucguggucuacaagggcgacgucuacaacauggauuacccuccauuuggugcuggaaggccaggacaguuuggugauauucagucuaggaccccugagucaaaggacguguacgcuaauacccaguuggugcuucaaaggccugcuguggguacuguucaugugccuuauucucaggcuccgagcgguuucaaguacuggcuuaaagaaaggggcgcuucucuucagcauaccgcuccauucgguugccagauugcuacuaauccugugagggcagugaauugcgcugugggcaauaugccuaucagcaucgauauucccgaggccgcuuucaccagaguuguggaugcuccuucucugaccgauaugucuugcgaaguuccugcuugcacccacuccucugauuuuggugguguggccaucaucaaguacgccgcuucuaagaaaggcaagugcgcugugcauagcaugacuaacgcugugaccauucgugaggcugagauugaaguugagggcaacucucagcugcagaucucuuucucuaccgcucuugcuucagcugaguucagaguucaggugugcucuacucagguucacugugcugcugaaugccauccuccuaaggaucacaucgugaacuacccugcuucucacacuacccuuggugugcaggauaucucugcuaccgcuaugucaugggugcagaagauuacuggcggaguuggucuuguuguggcuguugcugcucugauucuuaucguggugcucugcgugagcuucucuaggcauuagcUCGAGgauaccuccguugguauuguggguuugcuucugacuaccgcuauggcugcugagguuacaagaagggguagcgcuuacuacauguaccuggauaggaacgaugcuggugaggcuaucucuuucccuaccacccuugguaugaacaagugcuacauccagauuauggaucugggucacaugugugaugcuaccaugucuuacgagugcccuaugcuugaugaggguguugagccugaugauguggauugcuggugcaauaccacuucuaccugggugguguacgguacuugccaccauaagaagggugaggcaagaaggucuagaagggcuguuacucugccuagccacucuacuaggaagcuucagacuaggucacagaccuggcuugagucuagagaguacaccaagcaccuuaucaggguugagaacuggaucuuuaggaacccugguuucgcucuugcugcugcugcaauugcuuggcuucuuggaucuagcaccagccagaaggugaucuaccugguuaugauccugcugaucgcuccugcuuacagcaucagaugcaucggugugagcaacagggauuucguugagggaaugucuggugguacuuggguggacauugugcuugagcauggugguugcguuaccgugauggcucaagauaagccuaccguggauauugagcuugugaccaccaccguguccaauauggcugaggugaggucuuauugcuacgaggcuucuaucagcgauauggcuagcgauucuaggugcccuacucagggugaagcuuaccuugauaagcaguccgauacccaguacgugugcaagaggacucuuguugauagggguugggguaacgguugcggucuuuuugguaaggguucucuugugaccugcgcuaaguucgcuugcagcaagaagaugaccggaaagucuauccagccugagaaccuugaguacaggaucaugcuuagcgugcacgguucucagcacucugguaugauugugaacgauaccggacacgagacugaugagaauagggcuaagguugagaucaccccuaacucuccaagagcugaggcuacacuuggugguuucgguagccuuggucuugauugcgaaccuaggaccggacuggauuucagcgaucuuuacuaccugaccaugaacaacaagcacugguuggugcacaaagagugguuccacgauauuccuuugccuuggcaugcuggugcugauacugguacuccucacuggaacaacaaagaggcucugguugaguucaaggaugcucacgcuaagaggcagacuguuguggugcuuggaucucaagagggugcugugcauacugcauuagcuggugcucuugaggcugagauggauggugcaaaggguaggcuuucaucuggucaccuuaagugcaggcuuaagauggauaagcuuaggcugaagggugugagcuacucucugugcacugcugcuuuuaccuucaccaagaucccugcugagacucuucaugguacugugaccguugagguucaguacgcugguacugaugguccauguaagguuccagcucagauggcuguggauaugcagacucuuacuccuguggguaggcugauuacugcuaacccugugauuaccgagagcaccgagaacagcaagaugaugcuugagcuggauccuccauucggugauagcuacauugugaucggugugggugagaagaagaucacucaucacuggcacagguccgguucuaccauugguaaggcuuucgaggcuacugugagaggugcuaagaggauggcuguucuuggugauaccgcuugggauuucgguucuguuggaggugcucuuaacagccuggguaaggguauucaccagaucuucggugcugcuuucaagagccuuuucgguggaaugagcugguucucccagauucuuauuggaacccugcugauguggcugggucugaauaccaagaacgguagcaucucucugaugugccuugcucuugguggugugcugauuuuccuuagcaccgcugugucugcugauuag**Cucgag**gccuuuaacucugguuucauuaaauuuucuuuaguuugaauuuacuguuauucggugugcauuucuauguuuggugagcgguuuucugugcucagaguguguuuauuuuauguaauuuaauuucuuugugagcuccuguuuagcaggucgucccuucagcaaggacacaaaaagauuuuaauuuuauuaaaaaaaaaaaaaaaaaa

**> CHIKV-SP-ZIKAV-27% (encoded by plasmid pEAQ-CHIKV-SP-ZIKAV-27%)**

uauuaaaaucuuaauagguuuugauaaaagcgaacguggggaaacccgaaccaaaccuucuucuaaacucucucucaucucucuuaaagcaaacuucucucuugucuuucuugcaugagcgaucuucaacguugucagaucgugcuucggcaccaguacaauguuuucuuucacugaagcgaaaucaaagaucucuuuguggacacguagugcggcgccauuaaauaacguguacuuguccuauucuugucgguguggucuugggaaaagaaagcuugcuggaggcugcuguucagccccauacauuacuuguuacgauucugcugacuuucggcgggugcaauaucucuacuucugcuugacgagguauuguugccuguacuucuuucuucuucuucuugcugauugguucuauaagaaaucuaguauuuucuuugaaacagaguuuucccgugguuuucgaacuuggagaaagauuguuaagcuucuguauauucugcccaaauucg**accggu**caauggaauucaucccgacucagaccuucuacaacaggcguuaucaaccuaggccuuggacuccuaggccaaccauucaaguuauuaggccuagaccuaggccacagagacaagcuggucaacuugcucagcuuaucagcgcugugaacaagcuuacuaugagggcuguuccucagcagaagccuaggcguaaucggaagaacaagaagcagaagcaaaagcagcaggccccucagaacaauaccaaccagaagaagcagccgccuaagaagaagccugcucaaaagaagaagaaacccggccguagagaacggaugugcaugaagauugagaacgacugcaucuucgaggugaagcacgaagguaaggugacagguuacgcuugccuugugggugacaagguuaugaagccagcucacgugaagggcaccaucgauaaugcugaucuugcuaagcuggccuucaagcgguccucuaaguaugaucuugagugcgcucagaucccggugcacaugaagucugaugcuucuaaguucacccacgagaagccugagggcuacuacaauuggcaucauggugcugugcaguacagcggugguagguucacuauuccuacuggugcugguaagccuggugauucugguaggccuaucuucgauaacaagggcagagugguggcuaucguucuugguggugcuaaugagggugcuagaaccgcucuuucuguggugacuuggaacaaggacaucgugaccaagauuaccccugagggugcagaggaauggucccuugcuauuccugugaugugccugcuugccaacacuacuuucccuugcucucaaccuccuugcacuccuugcugcuaugagaaagagccugaagagacucugcggaugcuugaggauaaugugaugaggccugguuacuaccagcugcuucaggcuagucuuacuugcucuccucauaggcagcggaggucuaccaaggacaauuucaacguguacaaggccaccaggccuuacuuggcucauuguccugauuguggugagggacacucuugccauucuccaguugcucuugagcggauuaggaacgaggcuaccgauggcacccuuaagauucaggugagccuucagaucggcaucaagaccgaugauucucacgauuggaccaagcugcgguacauggauaaucacaugccugcugaugcugagagagcuggucuuuucguuaggacuucugcuccgugcacuauuaccggaacuaugggccacuucauucuggcuagauguccuaagggugagacucugacugugggcuucacugacucuaggaagaucucucacucuugcacucacccguuccaucaugauccuccagugaucgguagagagaaguuccauucuaggccucagcacgguaaagaguugccuugcucuacuuacgugcagucuacugcugcuaccaccgaagagauugagguucacaugccaccugauacaccagacaggacccuuaugucucagcagucugguaacgugaagaucaccgugaauggucagaccgugagguacaaguguaacugcggugguucuaacgagggacuuaccacuaccgacaaggugaucaacaacugcaagguggaccagugccaugcugcugugacuaaucauaagaaguggcaguacaacagcccgcuggugccuagaaaugcugagcuuggugauaggaagggcaagauucacaucccuuuuccgcuugcuaacgugaccugcagaguuccuaaggcuaggaauccuacugugaccuacggcaagaaccaggugaucaugcuucuguacccugaucacccuaccuugcugagcuauaggaacaugggcgaagaaccgaacuaccaagaggaaugggugaugcacaagaaagaaguggugcugacuguuccuaccgagggucuugaaguuaccugggguaacaacgagccguacaaguauuggccucagcugucuacuaacggaacugcucauggucacccucacgagaucauccuguacuacuacgagcuguacccuaccaugacugugguuguugugucuguggcuaccuucauccugcugucuaugguuggaauggcugcugguaugugcaugugcgcuagaaggcguugcauuaccccuuacgaacuuacuccuggugcuaccgugccuuuccuucugucucugauuugcugcaucaggaccgcuaaggcugcuaccuaucaagaggcugcuaucuaccuuuggaacgagcagcagccuuuguucuggcugcaagcucuuauuccucuggcugcucuuaucguccugugcaacugccuuagacuucugccuuguugcugcaagacccuggcuuucuuggcugugaugucugugggugcucauaccguuucugcuuacgagcaugugaccgugauuccuaauacugugggcgugccauacaagacccuugugaauaggccuggcuacagcccuauggugcuugagauggaacuuuugagcgugacccuugagccuacacugagccuugauuacauuaccugcgaguacaagacugugaucccgucaccauacguuaaguguugcgguacugcugaguguaaggacaagaaccugccugacuacucuugcaagguguucacugguguguacccuuucaugugggguggugccuauugcuucugugacgcugagaauacccagcugucugaggcucauguugagaagagugagucaugcaagaccgaguucgcuucagcuuacagggcucauacugcuuccgcuagcgcuaaguugagagugcuuuaccagggcaacaacauuaccgugaccgcuuacgcuaauggcgaucaugcuguuaccgugaaggacgccaaguucauugugggcccuaugucuagugcuuggaccccuuuugacaacaagaucguggucuacaagggcgacgucuacaacauggauuacccuccauuuggugcuggaaggccaggacaguuuggugauauucagucuaggaccccugagucaaaggacguguacgcuaauacccaguuggugcuucaaaggccugcuguggguacuguucaugugccuuauucucaggcuccgagcgguuucaaguacuggcuuaaagaaaggggcgcuucucuucagcauaccgcuccauucgguugccagauugcuacuaauccugugagggcagugaauugcgcugugggcaauaugccuaucagcaucgauauucccgaggccgcuuucaccagaguuguggaugcuccuucucugaccgauaugucuugcgaaguuccugcuugcacccacuccucugauuuuggugguguggccaucaucaaguacgccgcuucuaagaaaggcaagugcgcugugcauagcaugacuaacgcugugaccauucgugaggcugagauugaaguugagggcaacucucagcugcagaucucuuucucuaccgcucuugcuucagcugaguucagaguucaggugugcucuacucagguucacugugcugcugaaugccauccuccuaaggaucacaucgugaacuacccugcuucucacacuacccuuggugugcaggauaucucugcuaccgcuaugucaugggugcagaagauuacuggcggaguuggucuuguuguggcuguugcugcucugauucuuaucguggugcucugcgugagcuucucuaggcauuagcUCGAGgcuucuguauauucugcccaaauucaccgguaugaagaacccuaagaaaaagagcggugguuucaggaucgugaacaugcuuaagagggguguggcuaggguuucaccuuucgguggucuuaagagacuuccugcuggucugcuucuuggucacgguccuauuaggauggugcuugcuauucuggcuuuccugagguucaccgcuaucaagccuucucugggucuuaucaacaggugggguagcguggguaagaaagaagcuauggaaaucaucaagaaguucaagaaagaucuggcugcuaugcugaggaucaucaacgcuaggaaagaaaagaagagaaggggagcugauaccuccguugguauuguggguuugcuucugacuaccgcuauggcugcugagguuacaagaagggguagcgcuuacuacauguaccuggauaggaacgaugcuggugaggcuaucucuuucccuaccacccuugguaugaacaagugcuacauccagauuauggaucugggucacaugugugaugcuaccaugucuuacgagugcccuaugcuugaugaggguguugagccugaugauguggauugcuggugcaauaccacuucuaccugggugguguacgguacuugccaccauaagaagggugaggcaagaaggucuagaagggcuguuacucugccuagccacucuacuaggaagcuucagacuaggucacagaccuggcuugagucuagagaguacaccaagcaccuuaucaggguugagaacuggaucuuuaggaacccugguuucgcucuugcugcugcugcaauugcuuggcuucuuggaucuagcaccagccagaaggugaucuaccugguuaugauccugcugaucgcuccugcuuacagcaucagaugcaucggugugagcaacagggauuucguugagggaaugucuggugguacuuggguggacauugugcuugagcauggugguugcguuaccgugauggcucaagauaagccuaccguggauauugagcuugugaccaccaccguguccaauauggcugaggugaggucuuauugcuacgaggcuucuaucagcgauauggcuagcgauucuaggugcccuacucagggugaagcuuaccuugauaagcaguccgauacccaguacgugugcaagaggacucuuguugauagggguugggguaacgguugcggucuuuuugguaaggguucucuugugaccugcgcuaaguucgcuugcagcaagaagaugaccggaaagucuauccagccugagaaccuugaguacaggaucaugcuuagcgugcacgguucucagcacucugguaugauugugaacgauaccggacacgagacugaugagaauagggcuaagguugagaucaccccuaacucuccaagagcugaggcuacacuuggugguuucgguagccuuggucuugauugcgaaccuaggaccggacuggauuucagcgaucuuuacuaccugaccaugaacaacaagcacugguuggugcacaaagagugguuccacgauauuccuuugccuuggcaugcuggugcugauacugguacuccucacuggaacaacaaagaggcucugguugaguucaaggaugcucacgcuaagaggcagacuguuguggugcuuggaucucaagagggugcugugcauacugcauuagcuggugcucuugaggcugagauggauggugcaaaggguaggcuuucaucuggucaccuuaagugcaggcuuaagauggauaagcuuaggcugaagggugugagcuacucucugugcacugcugcuuuuaccuucaccaagaucccugcugagacucuucaugguacugugaccguugagguucaguacgcugguacugaugguccauguaagguuccagcucagauggcuguggauaugcagacucuuacuccuguggguaggcugauuacugcuaacccugugauuaccgagagcaccgagaacagcaagaugaugcuugagcuggauccuccauucggugauagcuacauugugaucggugugggugagaagaagaucacucaucacuggcacagguccgguucuaccauugguaaggcuuucgaggcuacugugagaggugcuaagaggauggcuguucuuggugauaccgcuugggauuucgguucuguuggaggugcucuuaacagccuggguaaggguauucaccagaucuucggugcugcuuucaagagccuuuucgguggaaugagcugguucucccagauucuuauuggaacccugcugauguggcugggucugaauaccaagaacgguagcaucucucugaugugccuugcucuugguggugugcugauuuuccuuagcaccgcugugucugcugauuag**Cucgag**gccuuuaacucugguuucauuaaauuuucuuuaguuugaauuuacuguuauucggugugcauuucuauguuuggugagcgguuuucugugcucagaguguguuuauuuuauguaauuuaauuucuuugugagcuccuguuuagcaggucgucccuucagcaaggacacaaaaagauuuuaauuuuauuaaaaaaaaaaaaaaaaaa

**Supplementary Part 2**: RNA sequences of the CPMV RNA-1 - based constructs described in this manuscript and shown in Figure 1. The 5’ and 3’ UTR sequences of CPMV RNA-1 are highlighted in yellow, Start and Stop codons of the RNA-1 – encoded polyprotein are in bold and underlined. Mutation in the RdRp of RNA-1-AAA highlighted in purple, the 5S0 and 3S0 synthetic 5’ and 3’ UTR sequences (Peyret et al., 2019) are highlighted in green. The CPMV RNA-2 3’ UTR sequence is highlighted in blue. The RCMV B-RNA 3’ UTR sequence is highlighted in pink. Note the RNA sequences indicated here are based on sequencing of their respective expression plasmids.

**>CPMV RNA-1 (encoded by plasmid pEAQ-RNA-1-Int)**

UAUUAAAAUCAAUACAGGUUUUGAUAAAAGCGAACGUGGAGAAAUCCAAACCUUUCUUUCUUUCCUCAAUCUCUUCAAUUGCGAACGAAAUCCAAGCUUUGGUUUUGCUGAAACAAAUACACAACGUAUACUGAAUUUGGCAAAUUUCUCUCUCUCUCUCUGUCAUUUUCUUUCUUCUGUCGGGACUUUCUUAGUCUUGACCCAAC**AUG**GGUCUCCCAGAAUAUGAGGCCGAUAGUGAGGCUUUAUUAAGUCAACUCACUAUCGAAUUCACACCCGGCAUGACAGUUUCUUCAUUGUUGGCACAAGUCACCACUAAUGACUUUCACAGUGCCAUUGAGUUUUUUGCUGCAGAAAAAGCAGUAGACAUUGAGGGCGUUCAUUACAAUGCGUAUAUGCAACAAAUUAGGAAAAACCCUAGUUUAUUACGCAUUUCCGUGGUAGCUUAUGCUUUCCACGUUUCAGACAUGGUAGCUGAGACCAUGUCUUAUGAUGUUUAUGAAUUUCUGUAUAAACAUUAUGCCCUUUUCAUCUCUAAUCUGGUGACCAGAACACUCAGAUUUAAAGAGCUUUUGCUGUUCUGUAAGCAGCAAUUUCUGGAGAAAAUGCAAGCUUCAAUAGUCUGGGCUCCGGAACUUGAGCAAUAUCUUCAAGUUGAAGGGGAUGCUGUGGCUCAAGGAGUUUCACAACUGUUAUACAAGAUGGUCACUUGGGUGCCCACUUUUGUCAGAGGAGCAGUAGACUGGAGCGUUGAUGCGAUUUUGGUCAGUUUCAGGAAACAUUUUGAAAAGAUGGUUCAGGAGUAUGUGCCCAUGGCUCAUCGCGUUUGCAGUUGGCUGAGCCAACUAUGGGAUAAGAUCGUGCAAUGGAUCUCACAAGCAAGUGAGACCAUGGGUUGGUUUCUAGAUGGUUGUCGGGAUUUGAUGACUUGGGGAAUUGCCACUCUCGCAACAUGUAGUGCUCUCUCCCUGGUUGAGAAGCUGUUAGUCGCAAUGGGUUUUCUGGUUGAGCCUUUCGGCUUGAGUGGAAUCUUCUUGCGGACGGGAGUUGUUGCGGCAGCUUGUUAUAACUAUGGGACUAAUUCUAAGGGUUUUGCCGAGAUGAUGGCUUUGUUGUCAUUGGCGGCUAACUGUGUCUCUACAGUUAUAGUUGGUGGCUUUUUCCCUGGUGAAAAGGACAAUGCACAGAGUAGUCCUGUUAUCCUCUUAGAAGGAUUGGCUGGGCAGAUGCAAAACUUUUGUGAGACUACACUUGUCAGUGUUGGGAAAACAUGCACUGCCGUCAAUGCUAUCUCAACAUGUUGUGGGAAUCUGAAAGCACUGGCCGGAAGGAUCUUGGGCAUGCUCAGAGAUUUUAUCUGGAAGACUUUGGGCUUUGAGACCAGAUUUCUAGCAGAUGCAUCUUUGCUUUUUGGCGAGGAUGUUGAUGGAUGGCUCAAAGCAAUCAGUGAUCUGCGAGAUCAAUUUAUUGCCAAAUCAUACUGUUCGCAGGAUGAGAUGAUGCAGAUUUUGGUGUUGCUUGAAAAGGGAAGGCAGAUGCGGAAAAGUGGUCUUUCUAAAGGAGGCAUUUCUCCUGCUAUCAUUAAUCUGAUUCUCAAAGGGAUUAAUGAUCUUGAACAAUUGAACCGCAGCUGUUCAGUGCAAGGAGUAAGAGGAGUUAGGAAAAUGCCAUUUACCAUUUUCUUCCAAGGAAAGUCACGCACUGGUAAGAGUUUGCUGAUGAGUCAGGUUACAAAGGAUUUUCAGGAUCACUAUGGAUUGGGUGGAGAAACUGUGUACAGUAGAAAUCCUUGUGAUCAAUAUUGGAGUGGAUAUCGGCGGCAACCUUUUGUGCUGAUGGAUGAUUUUGCCGCCGUUGUUACUGAGCCGUCUGCUGAGGCUCAGAUGAUCAAUCUGAUUUCUAGUGCUCCAUAUCCUUUGAAUAUGGCUGGACUUGAAGAAAAAGGAAUUUGUUUUGAUUCUCAAUUUGUUUUUGUUUCCACCAACUUCUUGGAAGUAUCUCCUGAAGCCAAAGUUAGGGACGAUGAGGCUUUCAAGAACAGGAGACAUGUGAUUGUUCAGGUUUCAAAUGAUCCUGCCAAAGCAUAUGAUGCUGCAAAUUUUGCUAGCAACCAAAUUUACACCAUUUUGGCAUGGAAGGAUGGUCGAUACAACACCGUGUGCGUUAUUGAGGACUAUGAUGAGCUGGUGGCAUAUUUGUUGACUAGGAGUCAACAGCAUGCUGAAGAGCAGGAGAAGAAUCUUGCUAACAUGAUGAAGAGUGCUACAUUUGAAAGUCAUUUCAAAAGUUUAGUUGAAGUCCUUGAGCUCGGUUCUAUGAUAUCUGCUGGUUUUGAUAUCAUUCGGCCAGAAAAACUUCCUAGUGAAGCUAAGGAGAAGAGAGUCCUUUACAGUAUUCCCUACAAUGGGGAGUAUUGUAAUGCACUCAUUGAUGACAAUUACAAUGUUACUUGCUGGUUUGGUGAGUGUGUUGGUAAUCCUGAGCAGCUCUCUAAGUACAGUGAAAAGAUGCUUUUGGGUGCUUAUGAAUUUCUUCUGUGUUCUGAGAGCUUGAAUGUUGUAAUUCAGGCACAUUUGAAGGAAAUGGUUUGCCCUCACCAUUAUGACAAGGAGCUCAAUUUUAUUGGCAAGAUAGGAGAGACCUACUAUCACAAUCAGAUGGUUUCAAAUAUCGGCUCUAUGCAGAAAUGGCAUCGUGCCAUUCUGUUUGGAAUUGGGGUUCUCUUGGGAAAGGAAAAAGAGAAGACAUGGUACCAAGUUCAGGUUGCCAAUGUUAAACAAGCUCUUUACGACAUGUACACUAAGGAGAUUCGUGAUUGGCCCAUGCCGAUCAAAGUCACCUGUGGAAUUGUCUUGGCAGCUAUUGGGGGUAGUGCCUUUUGGAAAGUGUUUCAACAACUAGUGGGAAGCGGAAAUGGUCCAGUAUUGAUGGGUGUGGCUGCUGGAGCAUUCAGUGCUGAGCCUCAAAGUAGAAAGCCCAAUAGGUUUGAUAUGCAGCAAUACAGGUACAACAAUGUUCCUCUCAAGAGAAGAGUUUGGGCAGACGCACAAAUGUCUUUGGAUCAGAGUAGUGUUGCUAUCAUGUCUAAGUGUAGGGCUAAUCUGGUUUUUGGAGGCACUAAUUUGCAAAUAGUCAUGGUACCAGGAAGACGCUUUUUGGCAUGCAAACAUUUCUUCACCCACAUAAAGACCAAAUUGCGUGUGGAAAUAGUUAUGGAUGGAAGAAGGUACUAUCAUCAAUUUGAUCCUGCAAAUAUUUAUGAUAUACCUGAUUCUGAGUUGGUCUUGUACUCCCAUCCUAGCUUGGAAGACGUUUCCCAUUCUUGCUGGGAUCUGUUCUGUUGGGACCCAGACAAAGAAUUGCCUUCAGUAUUUGGAGCGGAUUUCUUGAGUUGUAAAUACAACAAGUUUGGGGGUUUUUAUGAGGCGCAAUAUGCUGAUAUCAAAGUGCGCACAAAGAAAGAAUGCCUUACCAUACAGAGUGGUAAUUAUGUGAACAAGGUGUCUCGCUAUCUUGAGUAUGAAGCUCCUACUAUCCCUGAGGAUUGUGGAUCUCUUGUGAUAGCACACAUUGGUGGGAAGCACAAGAUUGUGGGUGUUCAUGUUGCUGGUAUUCAAGGUAAGAUAGGAUGUGCUUCCUUAUUGCCACCAUUGGAGCCAAUAGCACAAGCGCAAGGUGCUGAGGAAUACUUUGAUUUUCUUCCAGCUGAAGAGAAUGUAUCUUCUGGAGUGGCUAUGGUAGCAGGACUCAAACAAGGAGUUUACAUACCAUUACCCACAAAAACAGCGCUAGUGGAGACCCCCUCCGAGUGGCAUUUGGACACACCAUGUGACAAAGUUCCUAGCAUUUUAGUUCCCACGGAUCCCCGAAUUCCUGCGCAACAUGAAGGAUAUGAUCCUGCUAAGAGUGGGGUUUCCAAGUAUUCCCAGCCUAUGUCUGCUCUGGACCCUGAGUUACUUGGCGAGGUGGCUAAUGAUGUUCUCGAGCUAUGGCAUGACUGCGCUGUAGAUUGGGACGAUUUUGGUGAAGUGUCUCUGGAGGAAGCUUUGAAUGGAUGUGAAGGAGUGGAAUAUAUGGAAAGGAUUCCAUUAGCAACUUCUGAGGGCUUUCCGCACAUUCUUUCUAGAAAUGGGAAAGAAAAGGGGAAAAGACGGUUUGUUCAGGGAGAUGAUUGUGUUGUCUCACUAAUUCCAGGAACUACUGUAGCCAAAGCUUAUGAGGAGUUGGAAGCAAGUGCACACAGAUUUGUUCCCGCUCUUGUUGGGAUUGAAUGUCCAAAAGAUGAGAAGUUGCCUAUGAGAAAGGUUUUUGAUAAGCCUAAGACCAGGUGUUUUACCAUUUUGCCAAUGGAAUAUAAUUUGGUCGUUCGUAGGAAGUUUCUGAAUUUUGUGCGCUUUAUCAUGGCCAAUCGUCACAGACUCAGUUGUCAAGUGGGUAUUAAUCCAUAUUCAAUGGAAUGGAGUCGCUUAGCAGCAAGGAUGAAAGAGAAAGGCAAUGAUGUCUUGUGUUGUGAUUAUAGCUCAUUCGAUGGCUUGCUUUCUAAGCAAGUGAUGGAUGUCAUUGCUAGCAUGAUCAAUGAACUUUGUGGUGGAGAGGAUCAACUCAAAAAUGCAAGGCGAAACUUGUUAAUGGCGUGUUGCUCUAGGUUGGCUAUUUGCAAGAAUACAGUAUGGAGAGUUGAGUGUGGUAUUCCUUCAGGGUUUCCAAUGACAGUGAUUGUGAAUAGCAUUUUUAAUGAGAUUCUCAUUCGCUAUCAUUACAAGAAACUCAUGCGCGAACAACAAGCUCCUGAACUGAUGGUACAGAGUUUUGAUAAACUCAUAGGGCUGGUGACUUAUGGUGAUGAUAAUCUGAUUUCAGUGAAUGCUGUUGUGACACCCUAUUUUGAUGGGAAGAAAUUGAAGCAAUCUUUGGCUCAGGGUGGUGUGACUAUCACUGAUGGUAAGGACAAAACAAGUUUGGAACUUCCUUUUCGCAGAUUGGAAGAAUGUGAUUUUCUCAAGAGAACUUUUGUUCAGAGGAGCAGUACCAUCUGGGACGCUCCAGAGGAUAAGGCAAGUUUGUGGUCGCAGCUUCAUUAUGUUAAUUGCAACAAUUGUGAGAAAGAAGUUGCUUAUUUGACUAAUGUUGUUAAUGUUCUUCGUGAACUUUAUAUGCAUAGUCCUCGGGAAGCCACAGAAUUUAGGAGGAAGGUCUUAAAGAAGGUCAGUUGGAUCACUAGUGGAGAUUUGCCUACUUUGGCACAAUUGCAAGAGUUCUAUGAGUACCAGCGGCAGCAAGGUGGGGCAGACAACAAUGACACUUGUGACUUGUUAACAAGUGUAGACUUGCUAGGUCCUCCUUUGUCUUUUGAGAAAGAAGCGAUGCACGGAUGCAAAGUGUCUGAAGAAAUCGUCACCAAGAAUUUGGCAUAUUACGAUUUCAAAAGGAAAGGUGAGGAUGAAGUGGUAUUUCUGUUCAAUACGCUCUAUCCUCAGAGUUCAUUGCCUGAUGGGUGUCACUCUGUGACCUGGUCUCAGGGUAGUGGAAGGGGAGGUUUGCCCACACAAAGUUGGAUGAGCUAUAAUAUAAGCAGGAAAGAUUCUAAUAUCAACAAGAUUAUUAGAACUGCUGUUUCUUCGAAGAAACGAGUGAUAUUCUGUGCUCGUGAUAAUAUGGUUCCUGUUAACAUUGUAGCUUUGCUCUGUGCUGUUAGAAACAAGCUGAUGCCCACUGCUGUAUCUAAUGCUACACUUGUCAAGGUGAUGGAAAAUGCCAAAGCUUUCAAGUUUUUACCAGAAGAGUUCAAUUUCGCUUUUUCUGAUGUU**UAG**GUAAAUAAUGCUUAUGUUUUUGUUUGCUCCUGUUUAGCAGGUCGUUCCUUCAGCAAGAACAACAAAAAUAUGUGUUUUUAUU

**>RNA-1-AAA (encoded by plasmid pEAQ-RNA-1-Int-AAA)**

UAUUAAAAUCAAUACAGGUUUUGAUAAAAGCGAACGUGGAGAAAUCCAAACCUUUCUUUCUUUCCUCAAUCUCUUCAAUUGCGAACGAAAUCCAAGCUUUGGUUUUGCUGAAACAAAUACACAACGUAUACUGAAUUUGGCAAAUUUCUCUCUCUCUCUCUGUCAUUUUCUUUCUUCUGUCGGGACUUUCUUAGUCUUGACCCAAC**AUG**GGUCUCCCAGAAUAUGAGGCCGAUAGUGAGGCUUUAUUAAGUCAACUCACUAUCGAAUUCACACCCGGCAUGACAGUUUCUUCAUUGUUGGCACAAGUCACCACUAAUGACUUUCACAGUGCCAUUGAGUUUUUUGCUGCAGAAAAAGCAGUAGACAUUGAGGGCGUUCAUUACAAUGCGUAUAUGCAACAAAUUAGGAAAAACCCUAGUUUAUUACGCAUUUCCGUGGUAGCUUAUGCUUUCCACGUUUCAGACAUGGUAGCUGAGACCAUGUCUUAUGAUGUUUAUGAAUUUCUGUAUAAACAUUAUGCCCUUUUCAUCUCUAAUCUGGUGACCAGAACACUCAGAUUUAAAGAGCUUUUGCUGUUCUGUAAGCAGCAAUUUCUGGAGAAAAUGCAAGCUUCAAUAGUCUGGGCUCCGGAACUUGAGCAAUAUCUUCAAGUUGAAGGGGAUGCUGUGGCUCAAGGAGUUUCACAACUGUUAUACAAGAUGGUCACUUGGGUGCCCACUUUUGUCAGAGGAGCAGUAGACUGGAGCGUUGAUGCGAUUUUGGUCAGUUUCAGGAAACAUUUUGAAAAGAUGGUUCAGGAGUAUGUGCCCAUGGCUCAUCGCGUUUGCAGUUGGCUGAGCCAACUAUGGGAUAAGAUCGUGCAAUGGAUCUCACAAGCAAGUGAGACCAUGGGUUGGUUUCUAGAUGGUUGUCGGGAUUUGAUGACUUGGGGAAUUGCCACUCUCGCAACAUGUAGUGCUCUCUCCCUGGUUGAGAAGCUGUUAGUCGCAAUGGGUUUUCUGGUUGAGCCUUUCGGCUUGAGUGGAAUCUUCUUGCGGACGGGAGUUGUUGCGGCAGCUUGUUAUAACUAUGGGACUAAUUCUAAGGGUUUUGCCGAGAUGAUGGCUUUGUUGUCAUUGGCGGCUAACUGUGUCUCUACAGUUAUAGUUGGUGGCUUUUUCCCUGGUGAAAAGGACAAUGCACAGAGUAGUCCUGUUAUCCUCUUAGAAGGAUUGGCUGGGCAGAUGCAAAACUUUUGUGAGACUACACUUGUCAGUGUUGGGAAAACAUGCACUGCCGUCAAUGCUAUCUCAACAUGUUGUGGGAAUCUGAAAGCACUGGCCGGAAGGAUCUUGGGCAUGCUCAGAGAUUUUAUCUGGAAGACUUUGGGCUUUGAGACCAGAUUUCUAGCAGAUGCAUCUUUGCUUUUUGGCGAGGAUGUUGAUGGAUGGCUCAAAGCAAUCAGUGAUCUGCGAGAUCAAUUUAUUGCCAAAUCAUACUGUUCGCAGGAUGAGAUGAUGCAGAUUUUGGUGUUGCUUGAAAAGGGAAGGCAGAUGCGGAAAAGUGGUCUUUCUAAAGGAGGCAUUUCUCCUGCUAUCAUUAAUCUGAUUCUCAAAGGGAUUAAUGAUCUUGAACAAUUGAACCGCAGCUGUUCAGUGCAAGGAGUAAGAGGAGUUAGGAAAAUGCCAUUUACCAUUUUCUUCCAAGGAAAGUCACGCACUGGUAAGAGUUUGCUGAUGAGUCAGGUUACAAAGGAUUUUCAGGAUCACUAUGGAUUGGGUGGAGAAACUGUGUACAGUAGAAAUCCUUGUGAUCAAUAUUGGAGUGGAUAUCGGCGGCAACCUUUUGUGCUGAUGGAUGAUUUUGCCGCCGUUGUUACUGAGCCGUCUGCUGAGGCUCAGAUGAUCAAUCUGAUUUCUAGUGCUCCAUAUCCUUUGAAUAUGGCUGGACUUGAAGAAAAAGGAAUUUGUUUUGAUUCUCAAUUUGUUUUUGUUUCCACCAACUUCUUGGAAGUAUCUCCUGAAGCCAAAGUUAGGGACGAUGAGGCUUUCAAGAACAGGAGACAUGUGAUUGUUCAGGUUUCAAAUGAUCCUGCCAAAGCAUAUGAUGCUGCAAAUUUUGCUAGCAACCAAAUUUACACCAUUUUGGCAUGGAAGGAUGGUCGAUACAACACCGUGUGCGUUAUUGAGGACUAUGAUGAGCUGGUGGCAUAUUUGUUGACUAGGAGUCAACAGCAUGCUGAAGAGCAGGAGAAGAAUCUUGCUAACAUGAUGAAGAGUGCUACAUUUGAAAGUCAUUUCAAAAGUUUAGUUGAAGUCCUUGAGCUCGGUUCUAUGAUAUCUGCUGGUUUUGAUAUCAUUCGGCCAGAAAAACUUCCUAGUGAAGCUAAGGAGAAGAGAGUCCUUUACAGUAUUCCCUACAAUGGGGAGUAUUGUAAUGCACUCAUUGAUGACAAUUACAAUGUUACUUGCUGGUUUGGUGAGUGUGUUGGUAAUCCUGAGCAGCUCUCUAAGUACAGUGAAAAGAUGCUUUUGGGUGCUUAUGAAUUUCUUCUGUGUUCUGAGAGCUUGAAUGUUGUAAUUCAGGCACAUUUGAAGGAAAUGGUUUGCCCUCACCAUUAUGACAAGGAGCUCAAUUUUAUUGGCAAGAUAGGAGAGACCUACUAUCACAAUCAGAUGGUUUCAAAUAUCGGCUCUAUGCAGAAAUGGCAUCGUGCCAUUCUGUUUGGAAUUGGGGUUCUCUUGGGAAAGGAAAAAGAGAAGACAUGGUACCAAGUUCAGGUUGCCAAUGUUAAACAAGCUCUUUACGACAUGUACACUAAGGAGAUUCGUGAUUGGCCCAUGCCGAUCAAAGUCACCUGUGGAAUUGUCUUGGCAGCUAUUGGGGGUAGUGCCUUUUGGAAAGUGUUUCAACAACUAGUGGGAAGCGGAAAUGGUCCAGUAUUGAUGGGUGUGGCUGCUGGAGCAUUCAGUGCUGAGCCUCAAAGUAGAAAGCCCAAUAGGUUUGAUAUGCAGCAAUACAGGUACAACAAUGUUCCUCUCAAGAGAAGAGUUUGGGCAGACGCACAAAUGUCUUUGGAUCAGAGUAGUGUUGCUAUCAUGUCUAAGUGUAGGGCUAAUCUGGUUUUUGGAGGCACUAAUUUGCAAAUAGUCAUGGUACCAGGAAGACGCUUUUUGGCAUGCAAACAUUUCUUCACCCACAUAAAGACCAAAUUGCGUGUGGAAAUAGUUAUGGAUGGAAGAAGGUACUAUCAUCAAUUUGAUCCUGCAAAUAUUUAUGAUAUACCUGAUUCUGAGUUGGUCUUGUACUCCCAUCCUAGCUUGGAAGACGUUUCCCAUUCUUGCUGGGAUCUGUUCUGUUGGGACCCAGACAAAGAAUUGCCUUCAGUAUUUGGAGCGGAUUUCUUGAGUUGUAAAUACAACAAGUUUGGGGGUUUUUAUGAGGCGCAAUAUGCUGAUAUCAAAGUGCGCACAAAGAAAGAAUGCCUUACCAUACAGAGUGGUAAUUAUGUGAACAAGGUGUCUCGCUAUCUUGAGUAUGAAGCUCCUACUAUCCCUGAGGAUUGUGGAUCUCUUGUGAUAGCACACAUUGGUGGGAAGCACAAGAUUGUGGGUGUUCAUGUUGCUGGUAUUCAAGGUAAGAUAGGAUGUGCUUCCUUAUUGCCACCAUUGGAGCCAAUAGCACAAGCGCAAGGUGCUGAGGAAUACUUUGAUUUUCUUCCAGCUGAAGAGAAUGUAUCUUCUGGAGUGGCUAUGGUAGCAGGACUCAAACAAGGAGUUUACAUACCAUUACCCACAAAAACAGCGCUAGUGGAGACCCCCUCCGAGUGGCAUUUGGACACACCAUGUGACAAAGUUCCUAGCAUUUUAGUUCCCACGGAUCCCCGAAUUCCUGCGCAACAUGAAGGAUAUGAUCCUGCUAAGAGUGGGGUUUCCAAGUAUUCCCAGCCUAUGUCUGCUCUGGACCCUGAGUUACUUGGCGAGGUGGCUAAUGAUGUUCUCGAGCUAUGGCAUGACUGCGCUGUAGAUUGGGACGAUUUUGGUGAAGUGUCUCUGGAGGAAGCUUUGAAUGGAUGUGAAGGAGUGGAAUAUAUGGAAAGGAUUCCAUUAGCAACUUCUGAGGGCUUUCCGCACAUUCUUUCUAGAAAUGGGAAAGAAAAGGGGAAAAGACGGUUUGUUCAGGCAGCUGCUUGUGUUGUCUCACUAAUUCCAGGAACUACUGUAGCCAAAGCUUAUGAGGAGUUGGAAGCAAGUGCACACAGAUUUGUUCCCGCUCUUGUUGGGAUUGAAUGUCCAAAAGAUGAGAAGUUGCCUAUGAGAAAGGUUUUUGAUAAGCCUAAGACCAGGUGUUUUACCAUUUUGCCAAUGGAAUAUAAUUUGGUCGUUCGUAGGAAGUUUCUGAAUUUUGUGCGCUUUAUCAUGGCCAAUCGUCACAGACUCAGUUGUCAAGUGGGUAUUAAUCCAUAUUCAAUGGAAUGGAGUCGCUUAGCAGCAAGGAUGAAAGAGAAAGGCAAUGAUGUCUUGUGUUGUGAUUAUAGCUCAUUCGAUGGCUUGCUUUCUAAGCAAGUGAUGGAUGUCAUUGCUAGCAUGAUCAAUGAACUUUGUGGUGGAGAGGAUCAACUCAAAAAUGCAAGGCGAAACUUGUUAAUGGCGUGUUGCUCUAGGUUGGCUAUUUGCAAGAAUACAGUAUGGAGAGUUGAGUGUGGUAUUCCUUCAGGGUUUCCAAUGACAGUGAUUGUGAAUAGCAUUUUUAAUGAGAUUCUCAUUCGCUAUCAUUACAAGAAACUCAUGCGCGAACAACAAGCUCCUGAACUGAUGGUACAGAGUUUUGAUAAACUCAUAGGGCUGGUGACUUAUGGUGAUGAUAAUCUGAUUUCAGUGAAUGCUGUUGUGACACCCUAUUUUGAUGGGAAGAAAUUGAAGCAAUCUUUGGCUCAGGGUGGUGUGACUAUCACUGAUGGUAAGGACAAAACAAGUUUGGAACUUCCUUUUCGCAGAUUGGAAGAAUGUGAUUUUCUCAAGAGAACUUUUGUUCAGAGGAGCAGUACCAUCUGGGACGCUCCAGAGGAUAAGGCAAGUUUGUGGUCGCAGCUUCAUUAUGUUAAUUGCAACAAUUGUGAGAAAGAAGUUGCUUAUUUGACUAAUGUUGUUAAUGUUCUUCGUGAACUUUAUAUGCAUAGUCCUCGGGAAGCCACAGAAUUUAGGAGGAAGGUCUUAAAGAAGGUCAGUUGGAUCACUAGUGGAGAUUUGCCUACUUUGGCACAAUUGCAAGAGUUCUAUGAGUACCAGCGGCAGCAAGGUGGGGCAGACAACAAUGACACUUGUGACUUGUUAACAAGUGUAGACUUGCUAGGUCCUCCUUUGUCUUUUGAGAAAGAAGCGAUGCACGGAUGCAAAGUGUCUGAAGAAAUCGUCACCAAGAAUUUGGCAUAUUACGAUUUCAAAAGGAAAGGUGAGGAUGAAGUGGUAUUUCUGUUCAAUACGCUCUAUCCUCAGAGUUCAUUGCCUGAUGGGUGUCACUCUGUGACCUGGUCUCAGGGUAGUGGAAGGGGAGGUUUGCCCACACAAAGUUGGAUGAGCUAUAAUAUAAGCAGGAAAGAUUCUAAUAUCAACAAGAUUAUUAGAACUGCUGUUUCUUCGAAGAAACGAGUGAUAUUCUGUGCUCGUGAUAAUAUGGUUCCUGUUAACAUUGUAGCUUUGCUCUGUGCUGUUAGAAACAAGCUGAUGCCCACUGCUGUAUCUAAUGCUACACUUGUCAAGGUGAUGGAAAAUGCCAAAGCUUUCAAGUUUUUACCAGAAGAGUUCAAUUUCGCUUUUUCUGAUGUU**UAG**GUAAAUAAUGCUUAUGUUUUUGUUUGCUCCUGUUUAGCAGGUCGUUCCUUCAGCAAGAACAACAAAAAUAUGUGUUUUUAUU

**> RNA-1-Δ5’ (encoded by plasmid pEAQ-RNA-1-Δ5’)**

AACA**AUG**GGUCUCCCAGAAUAUGAGGCCGAUAGUGAGGCUUUAUUAAGUCAACUCACUAUCGAAUUCACACCCGGCAUGACAGUUUCUUCAUUGUUGGCACAAGUCACCACUAAUGACUUUCACAGUGCCAUUGAGUUUUUUGCUGCAGAAAAAGCAGUAGACAUUGAGGGCGUUCAUUACAAUGCGUAUAUGCAACAAAUUAGGAAAAACCCUAGUUUAUUACGCAUUUCCGUGGUAGCUUAUGCUUUCCACGUUUCAGACAUGGUAGCUGAGACCAUGUCUUAUGAUGUUUAUGAAUUUCUGUAUAAACAUUAUGCCCUUUUCAUCUCUAAUCUGGUGACCAGAACACUCAGAUUUAAAGAGCUUUUGCUGUUCUGUAAGCAGCAAUUUCUGGAGAAAAUGCAAGCUUCAAUAGUCUGGGCUCCGGAACUUGAGCAAUAUCUUCAAGUUGAAGGGGAUGCUGUGGCUCAAGGAGUUUCACAACUGUUAUACAAGAUGGUCACUUGGGUGCCCACUUUUGUCAGAGGAGCAGUAGACUGGAGCGUUGAUGCGAUUUUGGUCAGUUUCAGGAAACAUUUUGAAAAGAUGGUUCAGGAGUAUGUGCCCAUGGCUCAUCGCGUUUGCAGUUGGCUGAGCCAACUAUGGGAUAAGAUCGUGCAAUGGAUCUCACAAGCAAGUGAGACCAUGGGUUGGUUUCUAGAUGGUUGUCGGGAUUUGAUGACUUGGGGAAUUGCCACUCUCGCAACAUGUAGUGCUCUCUCCCUGGUUGAGAAGCUGUUAGUCGCAAUGGGUUUUCUGGUUGAGCCUUUCGGCUUGAGUGGAAUCUUCUUGCGGACGGGAGUUGUUGCGGCAGCUUGUUAUAACUAUGGGACUAAUUCUAAGGGUUUUGCCGAGAUGAUGGCUUUGUUGUCAUUGGCGGCUAACUGUGUCUCUACAGUUAUAGUUGGUGGCUUUUUCCCUGGUGAAAAGGACAAUGCACAGAGUAGUCCUGUUAUCCUCUUAGAAGGAUUGGCUGGGCAGAUGCAAAACUUUUGUGAGACUACACUUGUCAGUGUUGGGAAAACAUGCACUGCCGUCAAUGCUAUCUCAACAUGUUGUGGGAAUCUGAAAGCACUGGCCGGAAGGAUCUUGGGCAUGCUCAGAGAUUUUAUCUGGAAGACUUUGGGCUUUGAGACCAGAUUUCUAGCAGAUGCAUCUUUGCUUUUUGGCGAGGAUGUUGAUGGAUGGCUCAAAGCAAUCAGUGAUCUGCGAGAUCAAUUUAUUGCCAAAUCAUACUGUUCGCAGGAUGAGAUGAUGCAGAUUUUGGUGUUGCUUGAAAAGGGAAGGCAGAUGCGGAAAAGUGGUCUUUCUAAAGGAGGCAUUUCUCCUGCUAUCAUUAAUCUGAUUCUCAAAGGGAUUAAUGAUCUUGAACAAUUGAACCGCAGCUGUUCAGUGCAAGGAGUAAGAGGAGUUAGGAAAAUGCCAUUUACCAUUUUCUUCCAAGGAAAGUCACGCACUGGUAAGAGUUUGCUGAUGAGUCAGGUUACAAAGGAUUUUCAGGAUCACUAUGGAUUGGGUGGAGAAACUGUGUACAGUAGAAAUCCUUGUGAUCAAUAUUGGAGUGGAUAUCGGCGGCAACCUUUUGUGCUGAUGGAUGAUUUUGCCGCCGUUGUUACUGAGCCGUCUGCUGAGGCUCAGAUGAUCAAUCUGAUUUCUAGUGCUCCAUAUCCUUUGAAUAUGGCUGGACUUGAAGAAAAAGGAAUUUGUUUUGAUUCUCAAUUUGUUUUUGUUUCCACCAACUUCUUGGAAGUAUCUCCUGAAGCCAAAGUUAGGGACGAUGAGGCUUUCAAGAACAGGAGACAUGUGAUUGUUCAGGUUUCAAAUGAUCCUGCCAAAGCAUAUGAUGCUGCAAAUUUUGCUAGCAACCAAAUUUACACCAUUUUGGCAUGGAAGGAUGGUCGAUACAACACCGUGUGCGUUAUUGAGGACUAUGAUGAGCUGGUGGCAUAUUUGUUGACUAGGAGUCAACAGCAUGCUGAAGAGCAGGAGAAGAAUCUUGCUAACAUGAUGAAGAGUGCUACAUUUGAAAGUCAUUUCAAAAGUUUAGUUGAAGUCCUUGAGCUCGGUUCUAUGAUAUCUGCUGGUUUUGAUAUCAUUCGGCCAGAAAAACUUCCUAGUGAAGCUAAGGAGAAGAGAGUCCUUUACAGUAUUCCCUACAAUGGGGAGUAUUGUAAUGCACUCAUUGAUGACAAUUACAAUGUUACUUGCUGGUUUGGUGAGUGUGUUGGUAAUCCUGAGCAGCUCUCUAAGUACAGUGAAAAGAUGCUUUUGGGUGCUUAUGAAUUUCUUCUGUGUUCUGAGAGCUUGAAUGUUGUAAUUCAGGCACAUUUGAAGGAAAUGGUUUGCCCUCACCAUUAUGACAAGGAGCUCAAUUUUAUUGGCAAGAUAGGAGAGACCUACUAUCACAAUCAGAUGGUUUCAAAUAUCGGCUCUAUGCAGAAAUGGCAUCGUGCCAUUCUGUUUGGAAUUGGGGUUCUCUUGGGAAAGGAAAAAGAGAAGACAUGGUACCAAGUUCAGGUUGCCAAUGUUAAACAAGCUCUUUACGACAUGUACACUAAGGAGAUUCGUGAUUGGCCCAUGCCGAUCAAAGUCACCUGUGGAAUUGUCUUGGCAGCUAUUGGGGGUAGUGCCUUUUGGAAAGUGUUUCAACAACUAGUGGGAAGCGGAAAUGGUCCAGUAUUGAUGGGUGUGGCUGCUGGAGCAUUCAGUGCUGAGCCUCAAAGUAGAAAGCCCAAUAGGUUUGAUAUGCAGCAAUACAGGUACAACAAUGUUCCUCUCAAGAGAAGAGUUUGGGCAGACGCACAAAUGUCUUUGGAUCAGAGUAGUGUUGCUAUCAUGUCUAAGUGUAGGGCUAAUCUGGUUUUUGGAGGCACUAAUUUGCAAAUAGUCAUGGUACCAGGAAGACGCUUUUUGGCAUGCAAACAUUUCUUCACCCACAUAAAGACCAAAUUGCGUGUGGAAAUAGUUAUGGAUGGAAGAAGGUACUAUCAUCAAUUUGAUCCUGCAAAUAUUUAUGAUAUACCUGAUUCUGAGUUGGUCUUGUACUCCCAUCCUAGCUUGGAAGACGUUUCCCAUUCUUGCUGGGAUCUGUUCUGUUGGGACCCAGACAAAGAAUUGCCUUCAGUAUUUGGAGCGGAUUUCUUGAGUUGUAAAUACAACAAGUUUGGGGGUUUUUAUGAGGCGCAAUAUGCUGAUAUCAAAGUGCGCACAAAGAAAGAAUGCCUUACCAUACAGAGUGGUAAUUAUGUGAACAAGGUGUCUCGCUAUCUUGAGUAUGAAGCUCCUACUAUCCCUGAGGAUUGUGGAUCUCUUGUGAUAGCACACAUUGGUGGGAAGCACAAGAUUGUGGGUGUUCAUGUUGCUGGUAUUCAAGGUAAGAUAGGAUGUGCUUCCUUAUUGCCACCAUUGGAGCCAAUAGCACAAGCGCAAGGUGCUGAGGAAUACUUUGAUUUUCUUCCAGCUGAAGAGAAUGUAUCUUCUGGAGUGGCUAUGGUAGCAGGACUCAAACAAGGAGUUUACAUACCAUUACCCACAAAAACAGCGCUAGUGGAGACCCCCUCCGAGUGGCAUUUGGACACACCAUGUGACAAAGUUCCUAGCAUUUUAGUUCCCACGGAUCCCCGAAUUCCUGCGCAACAUGAAGGAUAUGAUCCUGCUAAGAGUGGGGUUUCCAAGUAUUCCCAGCCUAUGUCUGCUCUGGACCCUGAGUUACUUGGCGAGGUGGCUAAUGAUGUUCUCGAGCUAUGGCAUGACUGCGCUGUAGAUUGGGACGAUUUUGGUGAAGUGUCUCUGGAGGAAGCUUUGAAUGGAUGUGAAGGAGUGGAAUAUAUGGAAAGGAUUCCAUUAGCAACUUCUGAGGGCUUUCCGCACAUUCUUUCUAGAAAUGGGAAAGAAAAGGGGAAAAGACGGUUUGUUCAGGGAGAUGAUUGUGUUGUCUCACUAAUUCCAGGAACUACUGUAGCCAAAGCUUAUGAGGAGUUGGAAGCAAGUGCACACAGAUUUGUUCCCGCUCUUGUUGGGAUUGAAUGUCCAAAAGAUGAGAAGUUGCCUAUGAGAAAGGUUUUUGAUAAGCCUAAGACCAGGUGUUUUACCAUUUUGCCAAUGGAAUAUAAUUUGGUCGUUCGUAGGAAGUUUCUGAAUUUUGUGCGCUUUAUCAUGGCCAAUCGUCACAGACUCAGUUGUCAAGUGGGUAUUAAUCCAUAUUCAAUGGAAUGGAGUCGCUUAGCAGCAAGGAUGAAAGAGAAAGGCAAUGAUGUCUUGUGUUGUGAUUAUAGCUCAUUCGAUGGCUUGCUUUCUAAGCAAGUGAUGGAUGUCAUUGCUAGCAUGAUCAAUGAACUUUGUGGUGGAGAGGAUCAACUCAAAAAUGCAAGGCGAAACUUGUUAAUGGCGUGUUGCUCUAGGUUGGCUAUUUGCAAGAAUACAGUAUGGAGAGUUGAGUGUGGUAUUCCUUCAGGGUUUCCAAUGACAGUGAUUGUGAAUAGCAUUUUUAAUGAGAUUCUCAUUCGCUAUCAUUACAAGAAACUCAUGCGCGAACAACAAGCUCCUGAACUGAUGGUACAGAGUUUUGAUAAACUCAUAGGGCUGGUGACUUAUGGUGAUGAUAAUCUGAUUUCAGUGAAUGCUGUUGUGACACCCUAUUUUGAUGGGAAGAAAUUGAAGCAAUCUUUGGCUCAGGGUGGUGUGACUAUCACUGAUGGUAAGGACAAAACAAGUUUGGAACUUCCUUUUCGCAGAUUGGAAGAAUGUGAUUUUCUCAAGAGAACUUUUGUUCAGAGGAGCAGUACCAUCUGGGACGCUCCAGAGGAUAAGGCAAGUUUGUGGUCGCAGCUUCAUUAUGUUAAUUGCAACAAUUGUGAGAAAGAAGUUGCUUAUUUGACUAAUGUUGUUAAUGUUCUUCGUGAACUUUAUAUGCAUAGUCCUCGGGAAGCCACAGAAUUUAGGAGGAAGGUCUUAAAGAAGGUCAGUUGGAUCACUAGUGGAGAUUUGCCUACUUUGGCACAAUUGCAAGAGUUCUAUGAGUACCAGCGGCAGCAAGGUGGGGCAGACAACAAUGACACUUGUGACUUGUUAACAAGUGUAGACUUGCUAGGUCCUCCUUUGUCUUUUGAGAAAGAAGCGAUGCACGGAUGCAAAGUGUCUGAAGAAAUCGUCACCAAGAAUUUGGCAUAUUACGAUUUCAAAAGGAAAGGUGAGGAUGAAGUGGUAUUUCUGUUCAAUACGCUCUAUCCUCAGAGUUCAUUGCCUGAUGGGUGUCACUCUGUGACCUGGUCUCAGGGUAGUGGAAGGGGAGGUUUGCCCACACAAAGUUGGAUGAGCUAUAAUAUAAGCAGGAAAGAUUCUAAUAUCAACAAGAUUAUUAGAACUGCUGUUUCUUCGAAGAAACGAGUGAUAUUCUGUGCUCGUGAUAAUAUGGUUCCUGUUAACAUUGUAGCUUUGCUCUGUGCUGUUAGAAACAAGCUGAUGCCCACUGCUGUAUCUAAUGCUACACUUGUCAAGGUGAUGGAAAAUGCCAAAGCUUUCAAGUUUUUACCAGAAGAGUUCAAUUUCGCUUUUUCUGAUGUU**UAG**GUAAAUAAUGCUUAUGUUUUUGUUUGCUCCUGUUUAGCAGGUCGUUCCUUCAGCAAGAACAACAAAAAUAUGUGUUUUUAU

**> RNA-1-5S0 (encoded by plasmid pEAQ-5S0-RNA-1)**

uuuaagagacgcaaccacaacgcucuaacgcaaucaaucuacauuauauuaaacgucucuaaaa**AUG**GGUCUCCCAGAAUAUGAGGCCGAUAGUGAGGCUUUAUUAAGUCAACUCACUAUCGAAUUCACACCCGGCAUGACAGUUUCUUCAUUGUUGGCACAAGUCACCACUAAUGACUUUCACAGUGCCAUUGAGUUUUUUGCUGCAGAAAAAGCAGUAGACAUUGAGGGCGUUCAUUACAAUGCGUAUAUGCAACAAAUUAGGAAAAACCCUAGUUUAUUACGCAUUUCCGUGGUAGCUUAUGCUUUCCACGUUUCAGACAUGGUAGCUGAGACCAUGUCUUAUGAUGUUUAUGAAUUUCUGUAUAAACAUUAUGCCCUUUUCAUCUCUAAUCUGGUGACCAGAACACUCAGAUUUAAAGAGCUUUUGCUGUUCUGUAAGCAGCAAUUUCUGGAGAAAAUGCAAGCUUCAAUAGUCUGGGCUCCGGAACUUGAGCAAUAUCUUCAAGUUGAAGGGGAUGCUGUGGCUCAAGGAGUUUCACAACUGUUAUACAAGAUGGUCACUUGGGUGCCCACUUUUGUCAGAGGAGCAGUAGACUGGAGCGUUGAUGCGAUUUUGGUCAGUUUCAGGAAACAUUUUGAAAAGAUGGUUCAGGAGUAUGUGCCCAUGGCUCAUCGCGUUUGCAGUUGGCUGAGCCAACUAUGGGAUAAGAUCGUGCAAUGGAUCUCACAAGCAAGUGAGACCAUGGGUUGGUUUCUAGAUGGUUGUCGGGAUUUGAUGACUUGGGGAAUUGCCACUCUCGCAACAUGUAGUGCUCUCUCCCUGGUUGAGAAGCUGUUAGUCGCAAUGGGUUUUCUGGUUGAGCCUUUCGGCUUGAGUGGAAUCUUCUUGCGGACGGGAGUUGUUGCGGCAGCUUGUUAUAACUAUGGGACUAAUUCUAAGGGUUUUGCCGAGAUGAUGGCUUUGUUGUCAUUGGCGGCUAACUGUGUCUCUACAGUUAUAGUUGGUGGCUUUUUCCCUGGUGAAAAGGACAAUGCACAGAGUAGUCCUGUUAUCCUCUUAGAAGGAUUGGCUGGGCAGAUGCAAAACUUUUGUGAGACUACACUUGUCAGUGUUGGGAAAACAUGCACUGCCGUCAAUGCUAUCUCAACAUGUUGUGGGAAUCUGAAAGCACUGGCCGGAAGGAUCUUGGGCAUGCUCAGAGAUUUUAUCUGGAAGACUUUGGGCUUUGAGACCAGAUUUCUAGCAGAUGCAUCUUUGCUUUUUGGCGAGGAUGUUGAUGGAUGGCUCAAAGCAAUCAGUGAUCUGCGAGAUCAAUUUAUUGCCAAAUCAUACUGUUCGCAGGAUGAGAUGAUGCAGAUUUUGGUGUUGCUUGAAAAGGGAAGGCAGAUGCGGAAAAGUGGUCUUUCUAAAGGAGGCAUUUCUCCUGCUAUCAUUAAUCUGAUUCUCAAAGGGAUUAAUGAUCUUGAACAAUUGAACCGCAGCUGUUCAGUGCAAGGAGUAAGAGGAGUUAGGAAAAUGCCAUUUACCAUUUUCUUCCAAGGAAAGUCACGCACUGGUAAGAGUUUGCUGAUGAGUCAGGUUACAAAGGAUUUUCAGGAUCACUAUGGAUUGGGUGGAGAAACUGUGUACAGUAGAAAUCCUUGUGAUCAAUAUUGGAGUGGAUAUCGGCGGCAACCUUUUGUGCUGAUGGAUGAUUUUGCCGCCGUUGUUACUGAGCCGUCUGCUGAGGCUCAGAUGAUCAAUCUGAUUUCUAGUGCUCCAUAUCCUUUGAAUAUGGCUGGACUUGAAGAAAAAGGAAUUUGUUUUGAUUCUCAAUUUGUUUUUGUUUCCACCAACUUCUUGGAAGUAUCUCCUGAAGCCAAAGUUAGGGACGAUGAGGCUUUCAAGAACAGGAGACAUGUGAUUGUUCAGGUUUCAAAUGAUCCUGCCAAAGCAUAUGAUGCUGCAAAUUUUGCUAGCAACCAAAUUUACACCAUUUUGGCAUGGAAGGAUGGUCGAUACAACACCGUGUGCGUUAUUGAGGACUAUGAUGAGCUGGUGGCAUAUUUGUUGACUAGGAGUCAACAGCAUGCUGAAGAGCAGGAGAAGAAUCUUGCUAACAUGAUGAAGAGUGCUACAUUUGAAAGUCAUUUCAAAAGUUUAGUUGAAGUCCUUGAGCUCGGUUCUAUGAUAUCUGCUGGUUUUGAUAUCAUUCGGCCAGAAAAACUUCCUAGUGAAGCUAAGGAGAAGAGAGUCCUUUACAGUAUUCCCUACAAUGGGGAGUAUUGUAAUGCACUCAUUGAUGACAAUUACAAUGUUACUUGCUGGUUUGGUGAGUGUGUUGGUAAUCCUGAGCAGCUCUCUAAGUACAGUGAAAAGAUGCUUUUGGGUGCUUAUGAAUUUCUUCUGUGUUCUGAGAGCUUGAAUGUUGUAAUUCAGGCACAUUUGAAGGAAAUGGUUUGCCCUCACCAUUAUGACAAGGAGCUCAAUUUUAUUGGCAAGAUAGGAGAGACCUACUAUCACAAUCAGAUGGUUUCAAAUAUCGGCUCUAUGCAGAAAUGGCAUCGUGCCAUUCUGUUUGGAAUUGGGGUUCUCUUGGGAAAGGAAAAAGAGAAGACAUGGUACCAAGUUCAGGUUGCCAAUGUUAAACAAGCUCUUUACGACAUGUACACUAAGGAGAUUCGUGAUUGGCCCAUGCCGAUCAAAGUCACCUGUGGAAUUGUCUUGGCAGCUAUUGGGGGUAGUGCCUUUUGGAAAGUGUUUCAACAACUAGUGGGAAGCGGAAAUGGUCCAGUAUUGAUGGGUGUGGCUGCUGGAGCAUUCAGUGCUGAGCCUCAAAGUAGAAAGCCCAAUAGGUUUGAUAUGCAGCAAUACAGGUACAACAAUGUUCCUCUCAAGAGAAGAGUUUGGGCAGACGCACAAAUGUCUUUGGAUCAGAGUAGUGUUGCUAUCAUGUCUAAGUGUAGGGCUAAUCUGGUUUUUGGAGGCACUAAUUUGCAAAUAGUCAUGGUACCAGGAAGACGCUUUUUGGCAUGCAAACAUUUCUUCACCCACAUAAAGACCAAAUUGCGUGUGGAAAUAGUUAUGGAUGGAAGAAGGUACUAUCAUCAAUUUGAUCCUGCAAAUAUUUAUGAUAUACCUGAUUCUGAGUUGGUCUUGUACUCCCAUCCUAGCUUGGAAGACGUUUCCCAUUCUUGCUGGGAUCUGUUCUGUUGGGACCCAGACAAAGAAUUGCCUUCAGUAUUUGGAGCGGAUUUCUUGAGUUGUAAAUACAACAAGUUUGGGGGUUUUUAUGAGGCGCAAUAUGCUGAUAUCAAAGUGCGCACAAAGAAAGAAUGCCUUACCAUACAGAGUGGUAAUUAUGUGAACAAGGUGUCUCGCUAUCUUGAGUAUGAAGCUCCUACUAUCCCUGAGGAUUGUGGAUCUCUUGUGAUAGCACACAUUGGUGGGAAGCACAAGAUUGUGGGUGUUCAUGUUGCUGGUAUUCAAGGUAAGAUAGGAUGUGCUUCCUUAUUGCCACCAUUGGAGCCAAUAGCACAAGCGCAAGGUGCUGAGGAAUACUUUGAUUUUCUUCCAGCUGAAGAGAAUGUAUCUUCUGGAGUGGCUAUGGUAGCAGGACUCAAACAAGGAGUUUACAUACCAUUACCCACAAAAACAGCGCUAGUGGAGACCCCCUCCGAGUGGCAUUUGGACACACCAUGUGACAAAGUUCCUAGCAUUUUAGUUCCCACGGAUCCCCGAAUUCCUGCGCAACAUGAAGGAUAUGAUCCUGCUAAGAGUGGGGUUUCCAAGUAUUCCCAGCCUAUGUCUGCUCUGGACCCUGAGUUACUUGGCGAGGUGGCUAAUGAUGUUCUCGAGCUAUGGCAUGACUGCGCUGUAGAUUGGGACGAUUUUGGUGAAGUGUCUCUGGAGGAAGCUUUGAAUGGAUGUGAAGGAGUGGAAUAUAUGGAAAGGAUUCCAUUAGCAACUUCUGAGGGCUUUCCGCACAUUCUUUCUAGAAAUGGGAAAGAAAAGGGGAAAAGACGGUUUGUUCAGGGAGAUGAUUGUGUUGUCUCACUAAUUCCAGGAACUACUGUAGCCAAAGCUUAUGAGGAGUUGGAAGCAAGUGCACACAGAUUUGUUCCCGCUCUUGUUGGGAUUGAAUGUCCAAAAGAUGAGAAGUUGCCUAUGAGAAAGGUUUUUGAUAAGCCUAAGACCAGGUGUUUUACCAUUUUGCCAAUGGAAUAUAAUUUGGUCGUUCGUAGGAAGUUUCUGAAUUUUGUGCGCUUUAUCAUGGCCAAUCGUCACAGACUCAGUUGUCAAGUGGGUAUUAAUCCAUAUUCAAUGGAAUGGAGUCGCUUAGCAGCAAGGAUGAAAGAGAAAGGCAAUGAUGUCUUGUGUUGUGAUUAUAGCUCAUUCGAUGGCUUGCUUUCUAAGCAAGUGAUGGAUGUCAUUGCUAGCAUGAUCAAUGAACUUUGUGGUGGAGAGGAUCAACUCAAAAAUGCAAGGCGAAACUUGUUAAUGGCGUGUUGCUCUAGGUUGGCUAUUUGCAAGAAUACAGUAUGGAGAGUUGAGUGUGGUAUUCCUUCAGGGUUUCCAAUGACAGUGAUUGUGAAUAGCAUUUUUAAUGAGAUUCUCAUUCGCUAUCAUUACAAGAAACUCAUGCGCGAACAACAAGCUCCUGAACUGAUGGUACAGAGUUUUGAUAAACUCAUAGGGCUGGUGACUUAUGGUGAUGAUAAUCUGAUUUCAGUGAAUGCUGUUGUGACACCCUAUUUUGAUGGGAAGAAAUUGAAGCAAUCUUUGGCUCAGGGUGGUGUGACUAUCACUGAUGGUAAGGACAAAACAAGUUUGGAACUUCCUUUUCGCAGAUUGGAAGAAUGUGAUUUUCUCAAGAGAACUUUUGUUCAGAGGAGCAGUACCAUCUGGGACGCUCCAGAGGAUAAGGCAAGUUUGUGGUCGCAGCUUCAUUAUGUUAAUUGCAACAAUUGUGAGAAAGAAGUUGCUUAUUUGACUAAUGUUGUUAAUGUUCUUCGUGAACUUUAUAUGCAUAGUCCUCGGGAAGCCACAGAAUUUAGGAGGAAGGUCUUAAAGAAGGUCAGUUGGAUCACUAGUGGAGAUUUGCCUACUUUGGCACAAUUGCAAGAGUUCUAUGAGUACCAGCGGCAGCAAGGUGGGGCAGACAACAAUGACACUUGUGACUUGUUAACAAGUGUAGACUUGCUAGGUCCUCCUUUGUCUUUUGAGAAAGAAGCGAUGCACGGAUGCAAAGUGUCUGAAGAAAUCGUCACCAAGAAUUUGGCAUAUUACGAUUUCAAAAGGAAAGGUGAGGAUGAAGUGGUAUUUCUGUUCAAUACGCUCUAUCCUCAGAGUUCAUUGCCUGAUGGGUGUCACUCUGUGACCUGGUCUCAGGGUAGUGGAAGGGGAGGUUUGCCCACACAAAGUUGGAUGAGCUAUAAUAUAAGCAGGAAAGAUUCUAAUAUCAACAAGAUUAUUAGAACUGCUGUUUCUUCGAAGAAACGAGUGAUAUUCUGUGCUCGUGAUAAUAUGGUUCCUGUUAACAUUGUAGCUUUGCUCUGUGCUGUUAGAAACAAGCUGAUGCCCACUGCUGUAUCUAAUGCUACACUUGUCAAGGUGAUGGAAAAUGCCAAAGCUUUCAAGUUUUUACCAGAAGAGUUCAAUUUCGCUUUUUCUGAUGUU**UAG**GUAAAUAAUGCUUAUGUUUUUGUUUGCUCCUGUUUAGCAGGUCGUUCCUUCAGCAAGAACAACAAAAAUAUGUGUUUUUAUU

**> RNA-1-3’UTR-Swap (encoded by plasmid pEAQ-RNA-1-3’UTR-Swap)**

UAUUAAAAUCAAUACAGGUUUUGAUAAAAGCGAACGUGGAGAAAUCCAAACCUUUCUUUCUUUCCUCAAUCUCUUCAAUUGCGAACGAAAUCCAAGCUUUGGUUUUGCUGAAACAAAUACACAACGUAUACUGAAUUUGGCAAAUUUCUCUCUCUCUCUCUGUCAUUUUCUUUCUUCUGUCGGGACUUUCUUAGUCUUGACCCAAC**AUG**GGUCUCCCAGAAUAUGAGGCCGAUAGUGAGGCUUUAUUAAGUCAACUCACUAUCGAAUUCACACCCGGCAUGACAGUUUCUUCAUUGUUGGCACAAGUCACCACUAAUGACUUUCACAGUGCCAUUGAGUUUUUUGCUGCAGAAAAAGCAGUAGACAUUGAGGGCGUUCAUUACAAUGCGUAUAUGCAACAAAUUAGGAAAAACCCUAGUUUAUUACGCAUUUCCGUGGUAGCUUAUGCUUUCCACGUUUCAGACAUGGUAGCUGAGACCAUGUCUUAUGAUGUUUAUGAAUUUCUGUAUAAACAUUAUGCCCUUUUCAUCUCUAAUCUGGUGACCAGAACACUCAGAUUUAAAGAGCUUUUGCUGUUCUGUAAGCAGCAAUUUCUGGAGAAAAUGCAAGCUUCAAUAGUCUGGGCUCCGGAACUUGAGCAAUAUCUUCAAGUUGAAGGGGAUGCUGUGGCUCAAGGAGUUUCACAACUGUUAUACAAGAUGGUCACUUGGGUGCCCACUUUUGUCAGAGGAGCAGUAGACUGGAGCGUUGAUGCGAUUUUGGUCAGUUUCAGGAAACAUUUUGAAAAGAUGGUUCAGGAGUAUGUGCCCAUGGCUCAUCGCGUUUGCAGUUGGCUGAGCCAACUAUGGGAUAAGAUCGUGCAAUGGAUCUCACAAGCAAGUGAGACCAUGGGUUGGUUUCUAGAUGGUUGUCGGGAUUUGAUGACUUGGGGAAUUGCCACUCUCGCAACAUGUAGUGCUCUCUCCCUGGUUGAGAAGCUGUUAGUCGCAAUGGGUUUUCUGGUUGAGCCUUUCGGCUUGAGUGGAAUCUUCUUGCGGACGGGAGUUGUUGCGGCAGCUUGUUAUAACUAUGGGACUAAUUCUAAGGGUUUUGCCGAGAUGAUGGCUUUGUUGUCAUUGGCGGCUAACUGUGUCUCUACAGUUAUAGUUGGUGGCUUUUUCCCUGGUGAAAAGGACAAUGCACAGAGUAGUCCUGUUAUCCUCUUAGAAGGAUUGGCUGGGCAGAUGCAAAACUUUUGUGAGACUACACUUGUCAGUGUUGGGAAAACAUGCACUGCCGUCAAUGCUAUCUCAACAUGUUGUGGGAAUCUGAAAGCACUGGCCGGAAGGAUCUUGGGCAUGCUCAGAGAUUUUAUCUGGAAGACUUUGGGCUUUGAGACCAGAUUUCUAGCAGAUGCAUCUUUGCUUUUUGGCGAGGAUGUUGAUGGAUGGCUCAAAGCAAUCAGUGAUCUGCGAGAUCAAUUUAUUGCCAAAUCAUACUGUUCGCAGGAUGAGAUGAUGCAGAUUUUGGUGUUGCUUGAAAAGGGAAGGCAGAUGCGGAAAAGUGGUCUUUCUAAAGGAGGCAUUUCUCCUGCUAUCAUUAAUCUGAUUCUCAAAGGGAUUAAUGAUCUUGAACAAUUGAACCGCAGCUGUUCAGUGCAAGGAGUAAGAGGAGUUAGGAAAAUGCCAUUUACCAUUUUCUUCCAAGGAAAGUCACGCACUGGUAAGAGUUUGCUGAUGAGUCAGGUUACAAAGGAUUUUCAGGAUCACUAUGGAUUGGGUGGAGAAACUGUGUACAGUAGAAAUCCUUGUGAUCAAUAUUGGAGUGGAUAUCGGCGGCAACCUUUUGUGCUGAUGGAUGAUUUUGCCGCCGUUGUUACUGAGCCGUCUGCUGAGGCUCAGAUGAUCAAUCUGAUUUCUAGUGCUCCAUAUCCUUUGAAUAUGGCUGGACUUGAAGAAAAAGGAAUUUGUUUUGAUUCUCAAUUUGUUUUUGUUUCCACCAACUUCUUGGAAGUAUCUCCUGAAGCCAAAGUUAGGGACGAUGAGGCUUUCAAGAACAGGAGACAUGUGAUUGUUCAGGUUUCAAAUGAUCCUGCCAAAGCAUAUGAUGCUGCAAAUUUUGCUAGCAACCAAAUUUACACCAUUUUGGCAUGGAAGGAUGGUCGAUACAACACCGUGUGCGUUAUUGAGGACUAUGAUGAGCUGGUGGCAUAUUUGUUGACUAGGAGUCAACAGCAUGCUGAAGAGCAGGAGAAGAAUCUUGCUAACAUGAUGAAGAGUGCUACAUUUGAAAGUCAUUUCAAAAGUUUAGUUGAAGUCCUUGAGCUCGGUUCUAUGAUAUCUGCUGGUUUUGAUAUCAUUCGGCCAGAAAAACUUCCUAGUGAAGCUAAGGAGAAGAGAGUCCUUUACAGUAUUCCCUACAAUGGGGAGUAUUGUAAUGCACUCAUUGAUGACAAUUACAAUGUUACUUGCUGGUUUGGUGAGUGUGUUGGUAAUCCUGAGCAGCUCUCUAAGUACAGUGAAAAGAUGCUUUUGGGUGCUUAUGAAUUUCUUCUGUGUUCUGAGAGCUUGAAUGUUGUAAUUCAGGCACAUUUGAAGGAAAUGGUUUGCCCUCACCAUUAUGACAAGGAGCUCAAUUUUAUUGGCAAGAUAGGAGAGACCUACUAUCACAAUCAGAUGGUUUCAAAUAUCGGCUCUAUGCAGAAAUGGCAUCGUGCCAUUCUGUUUGGAAUUGGGGUUCUCUUGGGAAAGGAAAAAGAGAAGACAUGGUACCAAGUUCAGGUUGCCAAUGUUAAACAAGCUCUUUACGACAUGUACACUAAGGAGAUUCGUGAUUGGCCCAUGCCGAUCAAAGUCACCUGUGGAAUUGUCUUGGCAGCUAUUGGGGGUAGUGCCUUUUGGAAAGUGUUUCAACAACUAGUGGGAAGCGGAAAUGGUCCAGUAUUGAUGGGUGUGGCUGCUGGAGCAUUCAGUGCUGAGCCUCAAAGUAGAAAGCCCAAUAGGUUUGAUAUGCAGCAAUACAGGUACAACAAUGUUCCUCUCAAGAGAAGAGUUUGGGCAGACGCACAAAUGUCUUUGGAUCAGAGUAGUGUUGCUAUCAUGUCUAAGUGUAGGGCUAAUCUGGUUUUUGGAGGCACUAAUUUGCAAAUAGUCAUGGUACCAGGAAGACGCUUUUUGGCAUGCAAACAUUUCUUCACCCACAUAAAGACCAAAUUGCGUGUGGAAAUAGUUAUGGAUGGAAGAAGGUACUAUCAUCAAUUUGAUCCUGCAAAUAUUUAUGAUAUACCUGAUUCUGAGUUGGUCUUGUACUCCCAUCCUAGCUUGGAAGACGUUUCCCAUUCUUGCUGGGAUCUGUUCUGUUGGGACCCAGACAAAGAAUUGCCUUCAGUAUUUGGAGCGGAUUUCUUGAGUUGUAAAUACAACAAGUUUGGGGGUUUUUAUGAGGCGCAAUAUGCUGAUAUCAAAGUGCGCACAAAGAAAGAAUGCCUUACCAUACAGAGUGGUAAUUAUGUGAACAAGGUGUCUCGCUAUCUUGAGUAUGAAGCUCCUACUAUCCCUGAGGAUUGUGGAUCUCUUGUGAUAGCACACAUUGGUGGGAAGCACAAGAUUGUGGGUGUUCAUGUUGCUGGUAUUCAAGGUAAGAUAGGAUGUGCUUCCUUAUUGCCACCAUUGGAGCCAAUAGCACAAGCGCAAGGUGCUGAGGAAUACUUUGAUUUUCUUCCAGCUGAAGAGAAUGUAUCUUCUGGAGUGGCUAUGGUAGCAGGACUCAAACAAGGAGUUUACAUACCAUUACCCACAAAAACAGCGCUAGUGGAGACCCCCUCCGAGUGGCAUUUGGACACACCAUGUGACAAAGUUCCUAGCAUUUUAGUUCCCACGGAUCCCCGAAUUCCUGCGCAACAUGAAGGAUAUGAUCCUGCUAAGAGUGGGGUUUCCAAGUAUUCCCAGCCUAUGUCUGCUCUGGACCCUGAGUUACUUGGCGAGGUGGCUAAUGAUGUUCUCGAGCUAUGGCAUGACUGCGCUGUAGAUUGGGACGAUUUUGGUGAAGUGUCUCUGGAGGAAGCUUUGAAUGGAUGUGAAGGAGUGGAAUAUAUGGAAAGGAUUCCAUUAGCAACUUCUGAGGGCUUUCCGCACAUUCUUUCUAGAAAUGGGAAAGAAAAGGGGAAAAGACGGUUUGUUCAGGGAGAUGAUUGUGUUGUCUCACUAAUUCCAGGAACUACUGUAGCCAAAGCUUAUGAGGAGUUGGAAGCAAGUGCACACAGAUUUGUUCCCGCUCUUGUUGGGAUUGAAUGUCCAAAAGAUGAGAAGUUGCCUAUGAGAAAGGUUUUUGAUAAGCCUAAGACCAGGUGUUUUACCAUUUUGCCAAUGGAAUAUAAUUUGGUCGUUCGUAGGAAGUUUCUGAAUUUUGUGCGCUUUAUCAUGGCCAAUCGUCACAGACUCAGUUGUCAAGUGGGUAUUAAUCCAUAUUCAAUGGAAUGGAGUCGCUUAGCAGCAAGGAUGAAAGAGAAAGGCAAUGAUGUCUUGUGUUGUGAUUAUAGCUCAUUCGAUGGCUUGCUUUCUAAGCAAGUGAUGGAUGUCAUUGCUAGCAUGAUCAAUGAACUUUGUGGUGGAGAGGAUCAACUCAAAAAUGCAAGGCGAAACUUGUUAAUGGCGUGUUGCUCUAGGUUGGCUAUUUGCAAGAAUACAGUAUGGAGAGUUGAGUGUGGUAUUCCUUCAGGGUUUCCAAUGACAGUGAUUGUGAAUAGCAUUUUUAAUGAGAUUCUCAUUCGCUAUCAUUACAAGAAACUCAUGCGCGAACAACAAGCUCCUGAACUGAUGGUACAGAGUUUUGAUAAACUCAUAGGGCUGGUGACUUAUGGUGAUGAUAAUCUGAUUUCAGUGAAUGCUGUUGUGACACCCUAUUUUGAUGGGAAGAAAUUGAAGCAAUCUUUGGCUCAGGGUGGUGUGACUAUCACUGAUGGUAAGGACAAAACAAGUUUGGAACUUCCUUUUCGCAGAUUGGAAGAAUGUGAUUUUCUCAAGAGAACUUUUGUUCAGAGGAGCAGUACCAUCUGGGACGCUCCAGAGGAUAAGGCAAGUUUGUGGUCGCAGCUUCAUUAUGUUAAUUGCAACAAUUGUGAGAAAGAAGUUGCUUAUUUGACUAAUGUUGUUAAUGUUCUUCGUGAACUUUAUAUGCAUAGUCCUCGGGAAGCCACAGAAUUUAGGAGGAAGGUCUUAAAGAAGGUCAGUUGGAUCACUAGUGGAGAUUUGCCUACUUUGGCACAAUUGCAAGAGUUCUAUGAGUACCAGCGGCAGCAAGGUGGGGCAGACAACAAUGACACUUGUGACUUGUUAACAAGUGUAGACUUGCUAGGUCCUCCUUUGUCUUUUGAGAAAGAAGCGAUGCACGGAUGCAAAGUGUCUGAAGAAAUCGUCACCAAGAAUUUGGCAUAUUACGAUUUCAAAAGGAAAGGUGAGGAUGAAGUGGUAUUUCUGUUCAAUACGCUCUAUCCUCAGAGUUCAUUGCCUGAUGGGUGUCACUCUGUGACCUGGUCUCAGGGUAGUGGAAGGGGAGGUUUGCCCACACAAAGUUGGAUGAGCUAUAAUAUAAGCAGGAAAGAUUCUAAUAUCAACAAGAUUAUUAGAACUGCUGUUUCUUCGAAGAAACGAGUGAUAUUCUGUGCUCGUGAUAAUAUGGUUCCUGUUAACAUUGUAGCUUUGCUCUGUGCUGUUAGAAACAAGCUGAUGCCCACUGCUGUAUCUAAUGCUACACUUGUCAAGGUGAUGGAAAAUGCCAAAGCUUUCAAGUUUUUACCAGAAGAGUUCAAUUUCGCUUUUUCUGAUGUU**UAG**cgcucugguuucauuaaauuuucuuuaguuugaauuuacuguuauucggugugcauuucuauguuuggugagcgguuuucugugcucagaguguguuuauuuuauguaauuuaauuucuuugugagcuccuguuuagcaggucgucccuucagcaaggacacaaaaagauuuuaauuuuauu

**> RNA-1-RCMV-B (encoded by plasmid pEAQ-RNA-1-RCMV-B)**

UAUUAAAAUCAAUACAGGUUUUGAUAAAAGCGAACGUGGAGAAAUCCAAACCUUUCUUUCUUUCCUCAAUCUCUUCAAUUGCGAACGAAAUCCAAGCUUUGGUUUUGCUGAAACAAAUACACAACGUAUACUGAAUUUGGCAAAUUUCUCUCUCUCUCUCUGUCAUUUUCUUUCUUCUGUCGGGACUUUCUUAGUCUUGACCCAAC**AUG**GGUCUCCCAGAAUAUGAGGCCGAUAGUGAGGCUUUAUUAAGUCAACUCACUAUCGAAUUCACACCCGGCAUGACAGUUUCUUCAUUGUUGGCACAAGUCACCACUAAUGACUUUCACAGUGCCAUUGAGUUUUUUGCUGCAGAAAAAGCAGUAGACAUUGAGGGCGUUCAUUACAAUGCGUAUAUGCAACAAAUUAGGAAAAACCCUAGUUUAUUACGCAUUUCCGUGGUAGCUUAUGCUUUCCACGUUUCAGACAUGGUAGCUGAGACCAUGUCUUAUGAUGUUUAUGAAUUUCUGUAUAAACAUUAUGCCCUUUUCAUCUCUAAUCUGGUGACCAGAACACUCAGAUUUAAAGAGCUUUUGCUGUUCUGUAAGCAGCAAUUUCUGGAGAAAAUGCAAGCUUCAAUAGUCUGGGCUCCGGAACUUGAGCAAUAUCUUCAAGUUGAAGGGGAUGCUGUGGCUCAAGGAGUUUCACAACUGUUAUACAAGAUGGUCACUUGGGUGCCCACUUUUGUCAGAGGAGCAGUAGACUGGAGCGUUGAUGCGAUUUUGGUCAGUUUCAGGAAACAUUUUGAAAAGAUGGUUCAGGAGUAUGUGCCCAUGGCUCAUCGCGUUUGCAGUUGGCUGAGCCAACUAUGGGAUAAGAUCGUGCAAUGGAUCUCACAAGCAAGUGAGACCAUGGGUUGGUUUCUAGAUGGUUGUCGGGAUUUGAUGACUUGGGGAAUUGCCACUCUCGCAACAUGUAGUGCUCUCUCCCUGGUUGAGAAGCUGUUAGUCGCAAUGGGUUUUCUGGUUGAGCCUUUCGGCUUGAGUGGAAUCUUCUUGCGGACGGGAGUUGUUGCGGCAGCUUGUUAUAACUAUGGGACUAAUUCUAAGGGUUUUGCCGAGAUGAUGGCUUUGUUGUCAUUGGCGGCUAACUGUGUCUCUACAGUUAUAGUUGGUGGCUUUUUCCCUGGUGAAAAGGACAAUGCACAGAGUAGUCCUGUUAUCCUCUUAGAAGGAUUGGCUGGGCAGAUGCAAAACUUUUGUGAGACUACACUUGUCAGUGUUGGGAAAACAUGCACUGCCGUCAAUGCUAUCUCAACAUGUUGUGGGAAUCUGAAAGCACUGGCCGGAAGGAUCUUGGGCAUGCUCAGAGAUUUUAUCUGGAAGACUUUGGGCUUUGAGACCAGAUUUCUAGCAGAUGCAUCUUUGCUUUUUGGCGAGGAUGUUGAUGGAUGGCUCAAAGCAAUCAGUGAUCUGCGAGAUCAAUUUAUUGCCAAAUCAUACUGUUCGCAGGAUGAGAUGAUGCAGAUUUUGGUGUUGCUUGAAAAGGGAAGGCAGAUGCGGAAAAGUGGUCUUUCUAAAGGAGGCAUUUCUCCUGCUAUCAUUAAUCUGAUUCUCAAAGGGAUUAAUGAUCUUGAACAAUUGAACCGCAGCUGUUCAGUGCAAGGAGUAAGAGGAGUUAGGAAAAUGCCAUUUACCAUUUUCUUCCAAGGAAAGUCACGCACUGGUAAGAGUUUGCUGAUGAGUCAGGUUACAAAGGAUUUUCAGGAUCACUAUGGAUUGGGUGGAGAAACUGUGUACAGUAGAAAUCCUUGUGAUCAAUAUUGGAGUGGAUAUCGGCGGCAACCUUUUGUGCUGAUGGAUGAUUUUGCCGCCGUUGUUACUGAGCCGUCUGCUGAGGCUCAGAUGAUCAAUCUGAUUUCUAGUGCUCCAUAUCCUUUGAAUAUGGCUGGACUUGAAGAAAAAGGAAUUUGUUUUGAUUCUCAAUUUGUUUUUGUUUCCACCAACUUCUUGGAAGUAUCUCCUGAAGCCAAAGUUAGGGACGAUGAGGCUUUCAAGAACAGGAGACAUGUGAUUGUUCAGGUUUCAAAUGAUCCUGCCAAAGCAUAUGAUGCUGCAAAUUUUGCUAGCAACCAAAUUUACACCAUUUUGGCAUGGAAGGAUGGUCGAUACAACACCGUGUGCGUUAUUGAGGACUAUGAUGAGCUGGUGGCAUAUUUGUUGACUAGGAGUCAACAGCAUGCUGAAGAGCAGGAGAAGAAUCUUGCUAACAUGAUGAAGAGUGCUACAUUUGAAAGUCAUUUCAAAAGUUUAGUUGAAGUCCUUGAGCUCGGUUCUAUGAUAUCUGCUGGUUUUGAUAUCAUUCGGCCAGAAAAACUUCCUAGUGAAGCUAAGGAGAAGAGAGUCCUUUACAGUAUUCCCUACAAUGGGGAGUAUUGUAAUGCACUCAUUGAUGACAAUUACAAUGUUACUUGCUGGUUUGGUGAGUGUGUUGGUAAUCCUGAGCAGCUCUCUAAGUACAGUGAAAAGAUGCUUUUGGGUGCUUAUGAAUUUCUUCUGUGUUCUGAGAGCUUGAAUGUUGUAAUUCAGGCACAUUUGAAGGAAAUGGUUUGCCCUCACCAUUAUGACAAGGAGCUCAAUUUUAUUGGCAAGAUAGGAGAGACCUACUAUCACAAUCAGAUGGUUUCAAAUAUCGGCUCUAUGCAGAAAUGGCAUCGUGCCAUUCUGUUUGGAAUUGGGGUUCUCUUGGGAAAGGAAAAAGAGAAGACAUGGUACCAAGUUCAGGUUGCCAAUGUUAAACAAGCUCUUUACGACAUGUACACUAAGGAGAUUCGUGAUUGGCCCAUGCCGAUCAAAGUCACCUGUGGAAUUGUCUUGGCAGCUAUUGGGGGUAGUGCCUUUUGGAAAGUGUUUCAACAACUAGUGGGAAGCGGAAAUGGUCCAGUAUUGAUGGGUGUGGCUGCUGGAGCAUUCAGUGCUGAGCCUCAAAGUAGAAAGCCCAAUAGGUUUGAUAUGCAGCAAUACAGGUACAACAAUGUUCCUCUCAAGAGAAGAGUUUGGGCAGACGCACAAAUGUCUUUGGAUCAGAGUAGUGUUGCUAUCAUGUCUAAGUGUAGGGCUAAUCUGGUUUUUGGAGGCACUAAUUUGCAAAUAGUCAUGGUACCAGGAAGACGCUUUUUGGCAUGCAAACAUUUCUUCACCCACAUAAAGACCAAAUUGCGUGUGGAAAUAGUUAUGGAUGGAAGAAGGUACUAUCAUCAAUUUGAUCCUGCAAAUAUUUAUGAUAUACCUGAUUCUGAGUUGGUCUUGUACUCCCAUCCUAGCUUGGAAGACGUUUCCCAUUCUUGCUGGGAUCUGUUCUGUUGGGACCCAGACAAAGAAUUGCCUUCAGUAUUUGGAGCGGAUUUCUUGAGUUGUAAAUACAACAAGUUUGGGGGUUUUUAUGAGGCGCAAUAUGCUGAUAUCAAAGUGCGCACAAAGAAAGAAUGCCUUACCAUACAGAGUGGUAAUUAUGUGAACAAGGUGUCUCGCUAUCUUGAGUAUGAAGCUCCUACUAUCCCUGAGGAUUGUGGAUCUCUUGUGAUAGCACACAUUGGUGGGAAGCACAAGAUUGUGGGUGUUCAUGUUGCUGGUAUUCAAGGUAAGAUAGGAUGUGCUUCCUUAUUGCCACCAUUGGAGCCAAUAGCACAAGCGCAAGGUGCUGAGGAAUACUUUGAUUUUCUUCCAGCUGAAGAGAAUGUAUCUUCUGGAGUGGCUAUGGUAGCAGGACUCAAACAAGGAGUUUACAUACCAUUACCCACAAAAACAGCGCUAGUGGAGACCCCCUCCGAGUGGCAUUUGGACACACCAUGUGACAAAGUUCCUAGCAUUUUAGUUCCCACGGAUCCCCGAAUUCCUGCGCAACAUGAAGGAUAUGAUCCUGCUAAGAGUGGGGUUUCCAAGUAUUCCCAGCCUAUGUCUGCUCUGGACCCUGAGUUACUUGGCGAGGUGGCUAAUGAUGUUCUCGAGCUAUGGCAUGACUGCGCUGUAGAUUGGGACGAUUUUGGUGAAGUGUCUCUGGAGGAAGCUUUGAAUGGAUGUGAAGGAGUGGAAUAUAUGGAAAGGAUUCCAUUAGCAACUUCUGAGGGCUUUCCGCACAUUCUUUCUAGAAAUGGGAAAGAAAAGGGGAAAAGACGGUUUGUUCAGGGAGAUGAUUGUGUUGUCUCACUAAUUCCAGGAACUACUGUAGCCAAAGCUUAUGAGGAGUUGGAAGCAAGUGCACACAGAUUUGUUCCCGCUCUUGUUGGGAUUGAAUGUCCAAAAGAUGAGAAGUUGCCUAUGAGAAAGGUUUUUGAUAAGCCUAAGACCAGGUGUUUUACCAUUUUGCCAAUGGAAUAUAAUUUGGUCGUUCGUAGGAAGUUUCUGAAUUUUGUGCGCUUUAUCAUGGCCAAUCGUCACAGACUCAGUUGUCAAGUGGGUAUUAAUCCAUAUUCAAUGGAAUGGAGUCGCUUAGCAGCAAGGAUGAAAGAGAAAGGCAAUGAUGUCUUGUGUUGUGAUUAUAGCUCAUUCGAUGGCUUGCUUUCUAAGCAAGUGAUGGAUGUCAUUGCUAGCAUGAUCAAUGAACUUUGUGGUGGAGAGGAUCAACUCAAAAAUGCAAGGCGAAACUUGUUAAUGGCGUGUUGCUCUAGGUUGGCUAUUUGCAAGAAUACAGUAUGGAGAGUUGAGUGUGGUAUUCCUUCAGGGUUUCCAAUGACAGUGAUUGUGAAUAGCAUUUUUAAUGAGAUUCUCAUUCGCUAUCAUUACAAGAAACUCAUGCGCGAACAACAAGCUCCUGAACUGAUGGUACAGAGUUUUGAUAAACUCAUAGGGCUGGUGACUUAUGGUGAUGAUAAUCUGAUUUCAGUGAAUGCUGUUGUGACACCCUAUUUUGAUGGGAAGAAAUUGAAGCAAUCUUUGGCUCAGGGUGGUGUGACUAUCACUGAUGGUAAGGACAAAACAAGUUUGGAACUUCCUUUUCGCAGAUUGGAAGAAUGUGAUUUUCUCAAGAGAACUUUUGUUCAGAGGAGCAGUACCAUCUGGGACGCUCCAGAGGAUAAGGCAAGUUUGUGGUCGCAGCUUCAUUAUGUUAAUUGCAACAAUUGUGAGAAAGAAGUUGCUUAUUUGACUAAUGUUGUUAAUGUUCUUCGUGAACUUUAUAUGCAUAGUCCUCGGGAAGCCACAGAAUUUAGGAGGAAGGUCUUAAAGAAGGUCAGUUGGAUCACUAGUGGAGAUUUGCCUACUUUGGCACAAUUGCAAGAGUUCUAUGAGUACCAGCGGCAGCAAGGUGGGGCAGACAACAAUGACACUUGUGACUUGUUAACAAGUGUAGACUUGCUAGGUCCUCCUUUGUCUUUUGAGAAAGAAGCGAUGCACGGAUGCAAAGUGUCUGAAGAAAUCGUCACCAAGAAUUUGGCAUAUUACGAUUUCAAAAGGAAAGGUGAGGAUGAAGUGGUAUUUCUGUUCAAUACGCUCUAUCCUCAGAGUUCAUUGCCUGAUGGGUGUCACUCUGUGACCUGGUCUCAGGGUAGUGGAAGGGGAGGUUUGCCCACACAAAGUUGGAUGAGCUAUAAUAUAAGCAGGAAAGAUUCUAAUAUCAACAAGAUUAUUAGAACUGCUGUUUCUUCGAAGAAACGAGUGAUAUUCUGUGCUCGUGAUAAUAUGGUUCCUGUUAACAUUGUAGCUUUGCUCUGUGCUGUUAGAAACAAGCUGAUGCCCACUGCUGUAUCUAAUGCUACACUUGUCAAGGUGAUGGAAAAUGCCAAAGCUUUCAAGUUUUUACCAGAAGAGUUCAAUUUCGCUUUUUCUGAUGUU**UAG**uguaauuuggaaauucguguuuucugugugucgugaguuuucguuuguuauuucuuuuggguguuuucuuccaauagaauaaauggaauuuauuccauuaguauaauaucuuucuuuaucuuauuuuaauauaguacuguugugguaugugauaaaguuuguguuuauu

**> pHRE-RNA-1 (encoded by plasmid pHRE-RNA-1-ORF)**

uuuaagagacgcaaccacaacgcucuaacgcaaucaaucuacauuauauuaaacgucucuaaaa**AUG**GGUCUCCCAGAAUAUGAGGCCGAUAGUGAGGCUUUAUUAAGUCAACUCACUAUCGAAUUCACACCCGGCAUGACAGUUUCUUCAUUGUUGGCACAAGUCACCACUAAUGACUUUCACAGUGCCAUUGAGUUUUUUGCUGCAGAAAAAGCAGUAGACAUUGAGGGCGUUCAUUACAAUGCGUAUAUGCAACAAAUUAGGAAAAACCCUAGUUUAUUACGCAUUUCCGUGGUAGCUUAUGCUUUCCACGUUUCAGACAUGGUAGCUGAGACCAUGUCUUAUGAUGUUUAUGAAUUUCUGUAUAAACAUUAUGCCCUUUUCAUCUCUAAUCUGGUGACCAGAACACUCAGAUUUAAAGAGCUUUUGCUGUUCUGUAAGCAGCAAUUUCUGGAGAAAAUGCAAGCUUCAAUAGUCUGGGCUCCGGAACUUGAGCAAUAUCUUCAAGUUGAAGGGGAUGCUGUGGCUCAAGGAGUUUCACAACUGUUAUACAAGAUGGUCACUUGGGUGCCCACUUUUGUCAGAGGAGCAGUAGACUGGAGCGUUGAUGCGAUUUUGGUCAGUUUCAGGAAACAUUUUGAAAAGAUGGUUCAGGAGUAUGUGCCCAUGGCUCAUCGCGUUUGCAGUUGGCUGAGCCAACUAUGGGAUAAGAUCGUGCAAUGGAUCUCACAAGCAAGUGAGACCAUGGGUUGGUUUCUAGAUGGUUGUCGGGAUUUGAUGACUUGGGGAAUUGCCACUCUCGCAACAUGUAGUGCUCUCUCCCUGGUUGAGAAGCUGUUAGUCGCAAUGGGUUUUCUGGUUGAGCCUUUCGGCUUGAGUGGAAUCUUCUUGCGGACGGGAGUUGUUGCGGCAGCUUGUUAUAACUAUGGGACUAAUUCUAAGGGUUUUGCCGAGAUGAUGGCUUUGUUGUCAUUGGCGGCUAACUGUGUCUCUACAGUUAUAGUUGGUGGCUUUUUCCCUGGUGAAAAGGACAAUGCACAGAGUAGUCCUGUUAUCCUCUUAGAAGGAUUGGCUGGGCAGAUGCAAAACUUUUGUGAGACUACACUUGUCAGUGUUGGGAAAACAUGCACUGCCGUCAAUGCUAUCUCAACAUGUUGUGGGAAUCUGAAAGCACUGGCCGGAAGGAUCUUGGGCAUGCUCAGAGAUUUUAUCUGGAAGACUUUGGGCUUUGAGACCAGAUUUCUAGCAGAUGCAUCUUUGCUUUUUGGCGAGGAUGUUGAUGGAUGGCUCAAAGCAAUCAGUGAUCUGCGAGAUCAAUUUAUUGCCAAAUCAUACUGUUCGCAGGAUGAGAUGAUGCAGAUUUUGGUGUUGCUUGAAAAGGGAAGGCAGAUGCGGAAAAGUGGUCUUUCUAAAGGAGGCAUUUCUCCUGCUAUCAUUAAUCUGAUUCUCAAAGGGAUUAAUGAUCUUGAACAAUUGAACCGCAGCUGUUCAGUGCAAGGAGUAAGAGGAGUUAGGAAAAUGCCAUUUACCAUUUUCUUCCAAGGAAAGUCACGCACUGGUAAGAGUUUGCUGAUGAGUCAGGUUACAAAGGAUUUUCAGGAUCACUAUGGAUUGGGUGGAGAAACUGUGUACAGUAGAAAUCCUUGUGAUCAAUAUUGGAGUGGAUAUCGGCGGCAACCUUUUGUGCUGAUGGAUGAUUUUGCCGCCGUUGUUACUGAGCCGUCUGCUGAGGCUCAGAUGAUCAAUCUGAUUUCUAGUGCUCCAUAUCCUUUGAAUAUGGCUGGACUUGAAGAAAAAGGAAUUUGUUUUGAUUCUCAAUUUGUUUUUGUUUCCACCAACUUCUUGGAAGUAUCUCCUGAAGCCAAAGUUAGGGACGAUGAGGCUUUCAAGAACAGGAGACAUGUGAUUGUUCAGGUUUCAAAUGAUCCUGCCAAAGCAUAUGAUGCUGCAAAUUUUGCUAGCAACCAAAUUUACACCAUUUUGGCAUGGAAGGAUGGUCGAUACAACACCGUGUGCGUUAUUGAGGACUAUGAUGAGCUGGUGGCAUAUUUGUUGACUAGGAGUCAACAGCAUGCUGAAGAGCAGGAGAAGAAUCUUGCUAACAUGAUGAAGAGUGCUACAUUUGAAAGUCAUUUCAAAAGUUUAGUUGAAGUCCUUGAGCUCGGUUCUAUGAUAUCUGCUGGUUUUGAUAUCAUUCGGCCAGAAAAACUUCCUAGUGAAGCUAAGGAGAAGAGAGUCCUUUACAGUAUUCCCUACAAUGGGGAGUAUUGUAAUGCACUCAUUGAUGACAAUUACAAUGUUACUUGCUGGUUUGGUGAGUGUGUUGGUAAUCCUGAGCAGCUCUCUAAGUACAGUGAAAAGAUGCUUUUGGGUGCUUAUGAAUUUCUUCUGUGUUCUGAGAGCUUGAAUGUUGUAAUUCAGGCACAUUUGAAGGAAAUGGUUUGCCCUCACCAUUAUGACAAGGAGCUCAAUUUUAUUGGCAAGAUAGGAGAGACCUACUAUCACAAUCAGAUGGUUUCAAAUAUCGGCUCUAUGCAGAAAUGGCAUCGUGCCAUUCUGUUUGGAAUUGGGGUUCUCUUGGGAAAGGAAAAAGAGAAGACAUGGUACCAAGUUCAGGUUGCCAAUGUUAAACAAGCUCUUUACGACAUGUACACUAAGGAGAUUCGUGAUUGGCCCAUGCCGAUCAAAGUCACCUGUGGAAUUGUCUUGGCAGCUAUUGGGGGUAGUGCCUUUUGGAAAGUGUUUCAACAACUAGUGGGAAGCGGAAAUGGUCCAGUAUUGAUGGGUGUGGCUGCUGGAGCAUUCAGUGCUGAGCCUCAAAGUAGAAAGCCCAAUAGGUUUGAUAUGCAGCAAUACAGGUACAACAAUGUUCCUCUCAAGAGAAGAGUUUGGGCAGACGCACAAAUGUCUUUGGAUCAGAGUAGUGUUGCUAUCAUGUCUAAGUGUAGGGCUAAUCUGGUUUUUGGAGGCACUAAUUUGCAAAUAGUCAUGGUACCAGGAAGACGCUUUUUGGCAUGCAAACAUUUCUUCACCCACAUAAAGACCAAAUUGCGUGUGGAAAUAGUUAUGGAUGGAAGAAGGUACUAUCAUCAAUUUGAUCCUGCAAAUAUUUAUGAUAUACCUGAUUCUGAGUUGGUCUUGUACUCCCAUCCUAGCUUGGAAGACGUUUCCCAUUCUUGCUGGGAUCUGUUCUGUUGGGACCCAGACAAAGAAUUGCCUUCAGUAUUUGGAGCGGAUUUCUUGAGUUGUAAAUACAACAAGUUUGGGGGUUUUUAUGAGGCGCAAUAUGCUGAUAUCAAAGUGCGCACAAAGAAAGAAUGCCUUACCAUACAGAGUGGUAAUUAUGUGAACAAGGUGUCUCGCUAUCUUGAGUAUGAAGCUCCUACUAUCCCUGAGGAUUGUGGAUCUCUUGUGAUAGCACACAUUGGUGGGAAGCACAAGAUUGUGGGUGUUCAUGUUGCUGGUAUUCAAGGUAAGAUAGGAUGUGCUUCCUUAUUGCCACCAUUGGAGCCAAUAGCACAAGCGCAAGGUGCUGAGGAAUACUUUGAUUUUCUUCCAGCUGAAGAGAAUGUAUCUUCUGGAGUGGCUAUGGUAGCAGGACUCAAACAAGGAGUUUACAUACCAUUACCCACAAAAACAGCGCUAGUGGAGACCCCCUCCGAGUGGCAUUUGGACACACCAUGUGACAAAGUUCCUAGCAUUUUAGUUCCCACGGAUCCCCGAAUUCCUGCGCAACAUGAAGGAUAUGAUCCUGCUAAGAGUGGGGUUUCCAAGUAUUCCCAGCCUAUGUCUGCUCUGGACCCUGAGUUACUUGGCGAGGUGGCUAAUGAUGUUCUCGAGCUAUGGCAUGACUGCGCUGUAGAUUGGGACGAUUUUGGUGAAGUGUCUCUGGAGGAAGCUUUGAAUGGAUGUGAAGGAGUGGAAUAUAUGGAAAGGAUUCCAUUAGCAACUUCUGAGGGCUUUCCGCACAUUCUUUCUAGAAAUGGGAAAGAAAAGGGGAAAAGACGGUUUGUUCAGGGAGAUGAUUGUGUUGUCUCACUAAUUCCAGGAACUACUGUAGCCAAAGCUUAUGAGGAGUUGGAAGCAAGUGCACACAGAUUUGUUCCCGCUCUUGUUGGGAUUGAAUGUCCAAAAGAUGAGAAGUUGCCUAUGAGAAAGGUUUUUGAUAAGCCUAAGACCAGGUGUUUUACCAUUUUGCCAAUGGAAUAUAAUUUGGUCGUUCGUAGGAAGUUUCUGAAUUUUGUGCGCUUUAUCAUGGCCAAUCGUCACAGACUCAGUUGUCAAGUGGGUAUUAAUCCAUAUUCAAUGGAAUGGAGUCGCUUAGCAGCAAGGAUGAAAGAGAAAGGCAAUGAUGUCUUGUGUUGUGAUUAUAGCUCAUUCGAUGGCUUGCUUUCUAAGCAAGUGAUGGAUGUCAUUGCUAGCAUGAUCAAUGAACUUUGUGGUGGAGAGGAUCAACUCAAAAAUGCAAGGCGAAACUUGUUAAUGGCGUGUUGCUCUAGGUUGGCUAUUUGCAAGAAUACAGUAUGGAGAGUUGAGUGUGGUAUUCCUUCAGGGUUUCCAAUGACAGUGAUUGUGAAUAGCAUUUUUAAUGAGAUUCUCAUUCGCUAUCAUUACAAGAAACUCAUGCGCGAACAACAAGCUCCUGAACUGAUGGUACAGAGUUUUGAUAAACUCAUAGGGCUGGUGACUUAUGGUGAUGAUAAUCUGAUUUCAGUGAAUGCUGUUGUGACACCCUAUUUUGAUGGGAAGAAAUUGAAGCAAUCUUUGGCUCAGGGUGGUGUGACUAUCACUGAUGGUAAGGACAAAACAAGUUUGGAACUUCCUUUUCGCAGAUUGGAAGAAUGUGAUUUUCUCAAGAGAACUUUUGUUCAGAGGAGCAGUACCAUCUGGGACGCUCCAGAGGAUAAGGCAAGUUUGUGGUCGCAGCUUCAUUAUGUUAAUUGCAACAAUUGUGAGAAAGAAGUUGCUUAUUUGACUAAUGUUGUUAAUGUUCUUCGUGAACUUUAUAUGCAUAGUCCUCGGGAAGCCACAGAAUUUAGGAGGAAGGUCUUAAAGAAGGUCAGUUGGAUCACUAGUGGAGAUUUGCCUACUUUGGCACAAUUGCAAGAGUUCUAUGAGUACCAGCGGCAGCAAGGUGGGGCAGACAACAAUGACACUUGUGACUUGUUAACAAGUGUAGACUUGCUAGGUCCUCCUUUGUCUUUUGAGAAAGAAGCGAUGCACGGAUGCAAAGUGUCUGAAGAAAUCGUCACCAAGAAUUUGGCAUAUUACGAUUUCAAAAGGAAAGGUGAGGAUGAAGUGGUAUUUCUGUUCAAUACGCUCUAUCCUCAGAGUUCAUUGCCUGAUGGGUGUCACUCUGUGACCUGGUCUCAGGGUAGUGGAAGGGGAGGUUUGCCCACACAAAGUUGGAUGAGCUAUAAUAUAAGCAGGAAAGAUUCUAAUAUCAACAAGAUUAUUAGAACUGCUGUUUCUUCGAAGAAACGAGUGAUAUUCUGUGCUCGUGAUAAUAUGGUUCCUGUUAACAUUGUAGCUUUGCUCUGUGCUGUUAGAAACAAGCUGAUGCCCACUGCUGUAUCUAAUGCUACACUUGUCAAGGUGAUGGAAAAUGCCAAAGCUUUCAAGUUUUUACCAGAAGAGUUCAAUUUCGCUUUUUCUGAUGUU**UAG**cgccgaagagcgcaucggaucuaauaauaaacuuaggcaauaaauuucgacaucauaauaaagccacguaucuacaucaacacaagauuaguauuuucaaaacuguuuucagcagaacacauauuuucauuuuuaacguaauuuucauuuagcguuaguacagucaccuucacagaacguuuggagaagugagggcucuucc

**> pHREAC-RNA-1 (encoded by plasmid pHREAC-RNA-1-ORF)**

uuuaagagacgcaaccacaacgcucuaacgcaaucaaucuacauuauauuaaacgucucuaaaa**AUG**GGUCUCCCAGAAUAUGAGGCCGAUAGUGAGGCUUUAUUAAGUCAACUCACUAUCGAAUUCACACCCGGCAUGACAGUUUCUUCAUUGUUGGCACAAGUCACCACUAAUGACUUUCACAGUGCCAUUGAGUUUUUUGCUGCAGAAAAAGCAGUAGACAUUGAGGGCGUUCAUUACAAUGCGUAUAUGCAACAAAUUAGGAAAAACCCUAGUUUAUUACGCAUUUCCGUGGUAGCUUAUGCUUUCCACGUUUCAGACAUGGUAGCUGAGACCAUGUCUUAUGAUGUUUAUGAAUUUCUGUAUAAACAUUAUGCCCUUUUCAUCUCUAAUCUGGUGACCAGAACACUCAGAUUUAAAGAGCUUUUGCUGUUCUGUAAGCAGCAAUUUCUGGAGAAAAUGCAAGCUUCAAUAGUCUGGGCUCCGGAACUUGAGCAAUAUCUUCAAGUUGAAGGGGAUGCUGUGGCUCAAGGAGUUUCACAACUGUUAUACAAGAUGGUCACUUGGGUGCCCACUUUUGUCAGAGGAGCAGUAGACUGGAGCGUUGAUGCGAUUUUGGUCAGUUUCAGGAAACAUUUUGAAAAGAUGGUUCAGGAGUAUGUGCCCAUGGCUCAUCGCGUUUGCAGUUGGCUGAGCCAACUAUGGGAUAAGAUCGUGCAAUGGAUCUCACAAGCAAGUGAGACCAUGGGUUGGUUUCUAGAUGGUUGUCGGGAUUUGAUGACUUGGGGAAUUGCCACUCUCGCAACAUGUAGUGCUCUCUCCCUGGUUGAGAAGCUGUUAGUCGCAAUGGGUUUUCUGGUUGAGCCUUUCGGCUUGAGUGGAAUCUUCUUGCGGACGGGAGUUGUUGCGGCAGCUUGUUAUAACUAUGGGACUAAUUCUAAGGGUUUUGCCGAGAUGAUGGCUUUGUUGUCAUUGGCGGCUAACUGUGUCUCUACAGUUAUAGUUGGUGGCUUUUUCCCUGGUGAAAAGGACAAUGCACAGAGUAGUCCUGUUAUCCUCUUAGAAGGAUUGGCUGGGCAGAUGCAAAACUUUUGUGAGACUACACUUGUCAGUGUUGGGAAAACAUGCACUGCCGUCAAUGCUAUCUCAACAUGUUGUGGGAAUCUGAAAGCACUGGCCGGAAGGAUCUUGGGCAUGCUCAGAGAUUUUAUCUGGAAGACUUUGGGCUUUGAGACCAGAUUUCUAGCAGAUGCAUCUUUGCUUUUUGGCGAGGAUGUUGAUGGAUGGCUCAAAGCAAUCAGUGAUCUGCGAGAUCAAUUUAUUGCCAAAUCAUACUGUUCGCAGGAUGAGAUGAUGCAGAUUUUGGUGUUGCUUGAAAAGGGAAGGCAGAUGCGGAAAAGUGGUCUUUCUAAAGGAGGCAUUUCUCCUGCUAUCAUUAAUCUGAUUCUCAAAGGGAUUAAUGAUCUUGAACAAUUGAACCGCAGCUGUUCAGUGCAAGGAGUAAGAGGAGUUAGGAAAAUGCCAUUUACCAUUUUCUUCCAAGGAAAGUCACGCACUGGUAAGAGUUUGCUGAUGAGUCAGGUUACAAAGGAUUUUCAGGAUCACUAUGGAUUGGGUGGAGAAACUGUGUACAGUAGAAAUCCUUGUGAUCAAUAUUGGAGUGGAUAUCGGCGGCAACCUUUUGUGCUGAUGGAUGAUUUUGCCGCCGUUGUUACUGAGCCGUCUGCUGAGGCUCAGAUGAUCAAUCUGAUUUCUAGUGCUCCAUAUCCUUUGAAUAUGGCUGGACUUGAAGAAAAAGGAAUUUGUUUUGAUUCUCAAUUUGUUUUUGUUUCCACCAACUUCUUGGAAGUAUCUCCUGAAGCCAAAGUUAGGGACGAUGAGGCUUUCAAGAACAGGAGACAUGUGAUUGUUCAGGUUUCAAAUGAUCCUGCCAAAGCAUAUGAUGCUGCAAAUUUUGCUAGCAACCAAAUUUACACCAUUUUGGCAUGGAAGGAUGGUCGAUACAACACCGUGUGCGUUAUUGAGGACUAUGAUGAGCUGGUGGCAUAUUUGUUGACUAGGAGUCAACAGCAUGCUGAAGAGCAGGAGAAGAAUCUUGCUAACAUGAUGAAGAGUGCUACAUUUGAAAGUCAUUUCAAAAGUUUAGUUGAAGUCCUUGAGCUCGGUUCUAUGAUAUCUGCUGGUUUUGAUAUCAUUCGGCCAGAAAAACUUCCUAGUGAAGCUAAGGAGAAGAGAGUCCUUUACAGUAUUCCCUACAAUGGGGAGUAUUGUAAUGCACUCAUUGAUGACAAUUACAAUGUUACUUGCUGGUUUGGUGAGUGUGUUGGUAAUCCUGAGCAGCUCUCUAAGUACAGUGAAAAGAUGCUUUUGGGUGCUUAUGAAUUUCUUCUGUGUUCUGAGAGCUUGAAUGUUGUAAUUCAGGCACAUUUGAAGGAAAUGGUUUGCCCUCACCAUUAUGACAAGGAGCUCAAUUUUAUUGGCAAGAUAGGAGAGACCUACUAUCACAAUCAGAUGGUUUCAAAUAUCGGCUCUAUGCAGAAAUGGCAUCGUGCCAUUCUGUUUGGAAUUGGGGUUCUCUUGGGAAAGGAAAAAGAGAAGACAUGGUACCAAGUUCAGGUUGCCAAUGUUAAACAAGCUCUUUACGACAUGUACACUAAGGAGAUUCGUGAUUGGCCCAUGCCGAUCAAAGUCACCUGUGGAAUUGUCUUGGCAGCUAUUGGGGGUAGUGCCUUUUGGAAAGUGUUUCAACAACUAGUGGGAAGCGGAAAUGGUCCAGUAUUGAUGGGUGUGGCUGCUGGAGCAUUCAGUGCUGAGCCUCAAAGUAGAAAGCCCAAUAGGUUUGAUAUGCAGCAAUACAGGUACAACAAUGUUCCUCUCAAGAGAAGAGUUUGGGCAGACGCACAAAUGUCUUUGGAUCAGAGUAGUGUUGCUAUCAUGUCUAAGUGUAGGGCUAAUCUGGUUUUUGGAGGCACUAAUUUGCAAAUAGUCAUGGUACCAGGAAGACGCUUUUUGGCAUGCAAACAUUUCUUCACCCACAUAAAGACCAAAUUGCGUGUGGAAAUAGUUAUGGAUGGAAGAAGGUACUAUCAUCAAUUUGAUCCUGCAAAUAUUUAUGAUAUACCUGAUUCUGAGUUGGUCUUGUACUCCCAUCCUAGCUUGGAAGACGUUUCCCAUUCUUGCUGGGAUCUGUUCUGUUGGGACCCAGACAAAGAAUUGCCUUCAGUAUUUGGAGCGGAUUUCUUGAGUUGUAAAUACAACAAGUUUGGGGGUUUUUAUGAGGCGCAAUAUGCUGAUAUCAAAGUGCGCACAAAGAAAGAAUGCCUUACCAUACAGAGUGGUAAUUAUGUGAACAAGGUGUCUCGCUAUCUUGAGUAUGAAGCUCCUACUAUCCCUGAGGAUUGUGGAUCUCUUGUGAUAGCACACAUUGGUGGGAAGCACAAGAUUGUGGGUGUUCAUGUUGCUGGUAUUCAAGGUAAGAUAGGAUGUGCUUCCUUAUUGCCACCAUUGGAGCCAAUAGCACAAGCGCAAGGUGCUGAGGAAUACUUUGAUUUUCUUCCAGCUGAAGAGAAUGUAUCUUCUGGAGUGGCUAUGGUAGCAGGACUCAAACAAGGAGUUUACAUACCAUUACCCACAAAAACAGCGCUAGUGGAGACCCCCUCCGAGUGGCAUUUGGACACACCAUGUGACAAAGUUCCUAGCAUUUUAGUUCCCACGGAUCCCCGAAUUCCUGCGCAACAUGAAGGAUAUGAUCCUGCUAAGAGUGGGGUUUCCAAGUAUUCCCAGCCUAUGUCUGCUCUGGACCCUGAGUUACUUGGCGAGGUGGCUAAUGAUGUUCUCGAGCUAUGGCAUGACUGCGCUGUAGAUUGGGACGAUUUUGGUGAAGUGUCUCUGGAGGAAGCUUUGAAUGGAUGUGAAGGAGUGGAAUAUAUGGAAAGGAUUCCAUUAGCAACUUCUGAGGGCUUUCCGCACAUUCUUUCUAGAAAUGGGAAAGAAAAGGGGAAAAGACGGUUUGUUCAGGGAGAUGAUUGUGUUGUCUCACUAAUUCCAGGAACUACUGUAGCCAAAGCUUAUGAGGAGUUGGAAGCAAGUGCACACAGAUUUGUUCCCGCUCUUGUUGGGAUUGAAUGUCCAAAAGAUGAGAAGUUGCCUAUGAGAAAGGUUUUUGAUAAGCCUAAGACCAGGUGUUUUACCAUUUUGCCAAUGGAAUAUAAUUUGGUCGUUCGUAGGAAGUUUCUGAAUUUUGUGCGCUUUAUCAUGGCCAAUCGUCACAGACUCAGUUGUCAAGUGGGUAUUAAUCCAUAUUCAAUGGAAUGGAGUCGCUUAGCAGCAAGGAUGAAAGAGAAAGGCAAUGAUGUCUUGUGUUGUGAUUAUAGCUCAUUCGAUGGCUUGCUUUCUAAGCAAGUGAUGGAUGUCAUUGCUAGCAUGAUCAAUGAACUUUGUGGUGGAGAGGAUCAACUCAAAAAUGCAAGGCGAAACUUGUUAAUGGCGUGUUGCUCUAGGUUGGCUAUUUGCAAGAAUACAGUAUGGAGAGUUGAGUGUGGUAUUCCUUCAGGGUUUCCAAUGACAGUGAUUGUGAAUAGCAUUUUUAAUGAGAUUCUCAUUCGCUAUCAUUACAAGAAACUCAUGCGCGAACAACAAGCUCCUGAACUGAUGGUACAGAGUUUUGAUAAACUCAUAGGGCUGGUGACUUAUGGUGAUGAUAAUCUGAUUUCAGUGAAUGCUGUUGUGACACCCUAUUUUGAUGGGAAGAAAUUGAAGCAAUCUUUGGCUCAGGGUGGUGUGACUAUCACUGAUGGUAAGGACAAAACAAGUUUGGAACUUCCUUUUCGCAGAUUGGAAGAAUGUGAUUUUCUCAAGAGAACUUUUGUUCAGAGGAGCAGUACCAUCUGGGACGCUCCAGAGGAUAAGGCAAGUUUGUGGUCGCAGCUUCAUUAUGUUAAUUGCAACAAUUGUGAGAAAGAAGUUGCUUAUUUGACUAAUGUUGUUAAUGUUCUUCGUGAACUUUAUAUGCAUAGUCCUCGGGAAGCCACAGAAUUUAGGAGGAAGGUCUUAAAGAAGGUCAGUUGGAUCACUAGUGGAGAUUUGCCUACUUUGGCACAAUUGCAAGAGUUCUAUGAGUACCAGCGGCAGCAAGGUGGGGCAGACAACAAUGACACUUGUGACUUGUUAACAAGUGUAGACUUGCUAGGUCCUCCUUUGUCUUUUGAGAAAGAAGCGAUGCACGGAUGCAAAGUGUCUGAAGAAAUCGUCACCAAGAAUUUGGCAUAUUACGAUUUCAAAAGGAAAGGUGAGGAUGAAGUGGUAUUUCUGUUCAAUACGCUCUAUCCUCAGAGUUCAUUGCCUGAUGGGUGUCACUCUGUGACCUGGUCUCAGGGUAGUGGAAGGGGAGGUUUGCCCACACAAAGUUGGAUGAGCUAUAAUAUAAGCAGGAAAGAUUCUAAUAUCAACAAGAUUAUUAGAACUGCUGUUUCUUCGAAGAAACGAGUGAUAUUCUGUGCUCGUGAUAAUAUGGUUCCUGUUAACAUUGUAGCUUUGCUCUGUGCUGUUAGAAACAAGCUGAUGCCCACUGCUGUAUCUAAUGCUACACUUGUCAAGGUGAUGGAAAAUGCCAAAGCUUUCAAGUUUUUACCAGAAGAGUUCAAUUUCGCUUUUUCUGAUGUU**UAG**cgcucugguuucauuaaauuuucuuuaguuugaauuuacuguuauucggugugcauuucuauguuuggugagcgguuuucugugcucagaguguguuuauuuuauguaauuuaauuucuuugugagcuccuguuuagcaggucgucccuucagcaaggacacaaaaagauuuuaauuuuauu
